# P-TRAP: a Panicle Trait Phenotyping tool

**DOI:** 10.1186/1471-2229-13-122

**Published:** 2013-08-29

**Authors:** Faroq AL-Tam, Helene Adam, António dos Anjos, Mathias Lorieux, Pierre Larmande, Alain Ghesquière, Stefan Jouannic, Hamid Reza Shahbazkia

**Affiliations:** 1 DEEI-FCT Universidade do Algarve, 8005-139 Faro, Portugal; 2 IRD, UMR DIADE, Genome and Development of Rice group, 911 Avenue Agropolis, 34394 Montpellier, France; 3 CIAT, Agrobiodiversity and Biotechnology Project, Cali, Colombia; 4 DCT, ISMAT - Instituto Superior Manuel Teixeira Gomes, 8500-508 Portimão, Portugal; 5 CCMAR-CIMAR Laboratório Associado Universidade do Algarve, 8005-139 Faro, Portugal

**Keywords:** Phenotyping, 2D images, Panicle, Seed, Structure, Rice

## Abstract

**Background:**

In crops, inflorescence complexity and the shape and size of the seed are among the most important characters that influence yield. For example, rice panicles vary considerably in the number and order of branches, elongation of the axis, and the shape and size of the seed. Manual low-throughput phenotyping methods are time consuming, and the results are unreliable. However, high-throughput image analysis of the qualitative and quantitative traits of rice panicles is essential for understanding the diversity of the panicle as well as for breeding programs.

**Results:**

This paper presents P-TRAP software (Panicle TRAit Phenotyping), a free open source application for high-throughput measurements of panicle architecture and seed-related traits. The software is written in Java and can be used with different platforms (the user-friendly Graphical User Interface (GUI) uses Netbeans Platform 7.3). The application offers three main tools: a tool for the analysis of panicle structure, a spikelet/grain counting tool, and a tool for the analysis of seed shape. The three tools can be used independently or simultaneously for analysis of the same image. Results are then reported in the Extensible Markup Language (XML) and Comma Separated Values (CSV) file formats. Images of rice panicles were used to evaluate the efficiency and robustness of the software. Compared to data obtained by manual processing, P-TRAP produced reliable results in a much shorter time. In addition, manual processing is not repeatable because dry panicles are vulnerable to damage. The software is very useful, practical and collects much more data than human operators.

**Conclusions:**

P-TRAP is a new open source software that automatically recognizes the structure of a panicle and the seeds on the panicle in numeric images. The software processes and quantifies several traits related to panicle structure, detects and counts the grains, and measures their shape parameters. In short, P-TRAP offers both efficient results and a user-friendly environment for experiments. The experimental results showed very good accuracy compared to field operator, expert verification and well-known academic methods.

## Background

**Figure 1:**
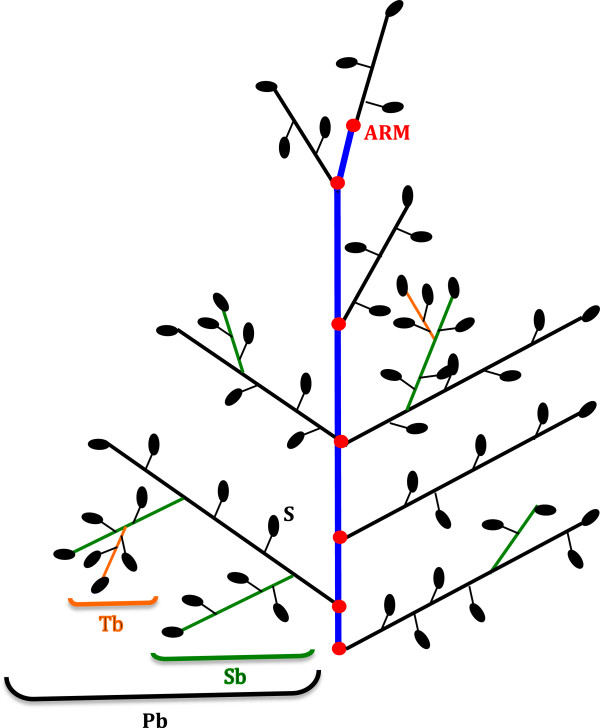
**Structure of a rice panicle.** Schematic representation of a rice panicle comprising a main central axis (blue line) named rachis, to which primary branches (Pb) are attached (black lines); the primary branches bear secondary branches (Sb, green lines), which in turn bear tertiary branches (Tb, orange lines). Spikelets (Sp) are attached to the branches by a peduncle. Nodes are represented by red dots (ARM, Aborted Rachis Meristem). In the P-TRAP output results, the following terms are used instead of botanical terminology: Primary Axis (PA) for the blue line, the secondary axes (SA) for the black lines; the tertiary axes (TA) for the green lines and the quaternary axes (QA) for the orange lines.

The architecture of the rice inflorescence (or panicle) is of major importance for rice breeding as it directly affects in the number of grains per panicle and hence final rice yield. The rice panicle is a complex branched structure consisting of a main axis (rachis) bearing lateral branches named primary branches (Pb) that bear so-called secondary branches (Sb), from which higher order branches may be observed (Figure 1). Primary, secondary and higher order branches bear spikelets consisting of glumes (bract-like organs) and florets. In rice, a spikelet contains a single fertile floret and a pair of sterile lemmas (also called ‘empty glumes’), subtended by a pair of highly reduced glumes called rudimentary glumes [1]. The number of spikelets (and consequently the number of grains) per panicle is therefore related to the branching complexity (number and order of branches). Panicle branching is a highly complex process that is influenced by genetic, hormonal and environmental factors (see [2] for a review of the genetic and molecular bases of rice yield). Several genes related to meristem formation or fate, hormone biosynthesis or response, that contribute to panicle branching complexity, have been identified in the cultivated Asian rice species *Oryza sativa* from quantitative trait loci (QTL) mapping populations and mutant analysis [2,3]. However, QTL mapping of panicle branching complexity indicates that this trait is under the control of many genes, that remain to be identified [2]. Moreover, rice species display a wide range of morphological traits (including panicle complexity) as well as their ecological habitat and their tolerance to abiotic and biotic stresses. The genus *Oryza* consists of about 23 species including only two cultivated species,*O. sativa* and *O. glaberrima*, which originate from Asia and Africa, respectively [4].

There is a wide range of rice panicle architecture among varieties concerning the number and order of branches, and axis elongation. Natural inter-specific and intra-specific variations in morphological traits represent a largely untapped highly valuable resource for genetic improvement by breeding. For efficient selection of beneficial alleles for breeding, natural variation needs to be well characterized at the phenotypic and molecular genetic level. In addition, the study of natural variation is also important to understand the evolution of morphological traits and the molecular genetic mechanisms underlying them. To exploit the diversity of rice panicle resources, panicle morphological traits need to be identified and quantified. Plant phenotyping involves screening large collections of accessions to facilitate the discovery of new interesting traits, and analyzing known phenotypic data to identify the genes involved in their diversity, to be able to use these genes in plant breeding. To collect these data, the usual procedure consists in laborious manual measurements on predefined traits such as panicle length, the number of branches, the order of branches, the number of grains, and grain size. Depending on the degree of complexity of the panicle, manual phenotypic analysis is time consuming and it is impossible to evaluate and quantify all traits (such as branch and spikelet positions in the panicle) to obtain an accurate overview of panicle architecture. Moreover, manual phenotyping is often destructive for the plant making it impossible to use the same panicle to measure other traits. Given the importance of gene discovery and crop improvement, there is thus an urgent need to automate such tedious and time- consuming tasks. The development of an easy high-throughput panicle phenotyping method should aim to standardize the measurement and extraction of panicle traits. In recent years, plant phenotyping research has led to the development of software for plant screening facilities. Recent image processing solutions, such as TraitMill and HTPheno, offer general analysis for the measurement of plant height, volume and colorimetry [5,6].

Other software provides 2-D image-based semi-automated processing for leaf phenotyping (Phenopsis or LAMINA) and root data monitoring (GROWSCREEN) [7-9]. Specific rice image-based solutions have been developed for phenotyping and involve the measurement of parameters such as grain size (length, width, and thickness), panicle length, and the number of tillers [10,11]. However, these methods could not be adapted to rice panicle structure phenotyping and require expensive equipment. Ikeda *et al*[12] developed a software named PASTAR (Panicle Structure Analyzer for Rice) and PASTA Viewer, to automatically extract values for length, number of branches, and number of grains from scanned panicle images. However, this software is under license, thus limiting access by the scientific community. Recently, a program named Smartgrain was developed to quantify seed shape and size. However, this software does not process the grain attached to panicles but only individual grains [13]. In this context, it was important to develop an easy-to-use freely available open-source software based on 2-D image processing for the analysis of rice panicle structure.

Here, we propose a Java-based stand-alone application named P-TRAP (for Panicle TRAit Phenotyping) to easily quantify 2-D panicle traits. The labor-intensive processing is automated but post-processing options allow users to improve the quality of the analysis using their expert knowledge. The proposed pipeline has different tools: a tool to analyze panicle structure, and a spikelet/grain counting and shape analysis tool. The software allows automatic detection of the structure of the panicle from a spread panicle image consisting of different morphological traits that are not easily accessible through manual phenotyping. The spikelet/grain counting option detects the grains on the panicle, counts them and quantifies different shape parameters. The novelty of P-TRAP is the simultaneous analysis of panicle structure and grain counting/shape on the same image. These shape parameters can also be measured from images of spread seeds. The interface allows the two analyses to be performed at the same time (or separately) and extracts the different traits in different output formats (CSV and XML) to facilitate data analysis and access to OpenAlea platform facilities [14]. In this study, we used this program to analyze the panicle structure of various accessions of *O. sativa*,*O. glaberrima* and *O. barthii* and to compare the results with manual measurements to check the robustness of the software.

## Implementation

P-TRAP is written in Java with a user-friendly GUI. The GUI is built on top of the Netbeans Platform (version 7.3), which provides a modular underlay for the system’s architecture. The software provides different features for users to conduct their experiments and edit and collect the final results. It offers an editor for the input image, the panicle structure and the grains. The user interaction is mostly performed by using the mouse or keyboard shortcuts. In addition, developers can easily add new features to the application, as it is very modular.

### Panicle trait calculation pipeline

#### Source images

**Figure 2:**
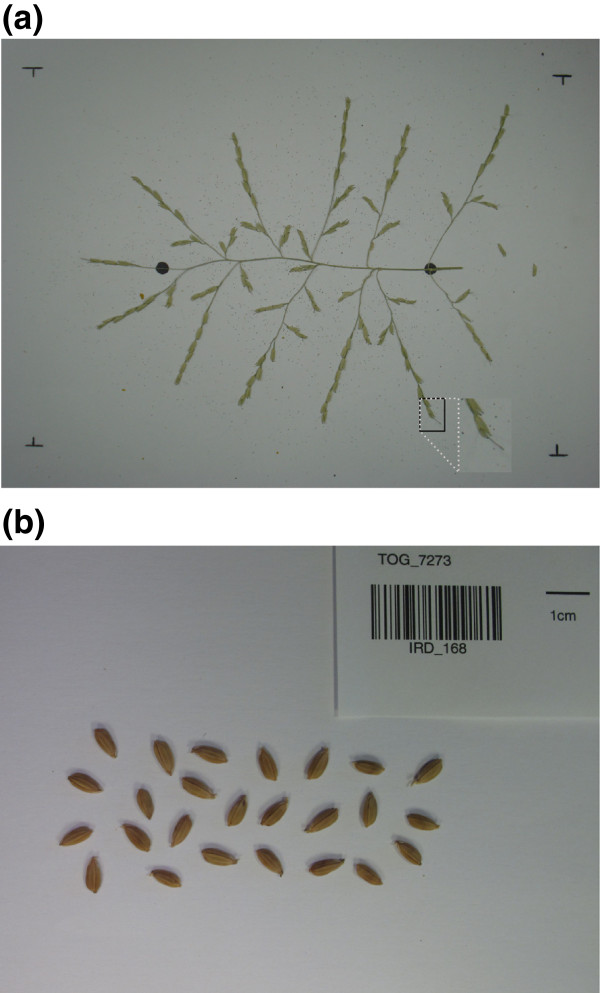
**Panicle and seed image preparation.** **(a)** The panicle is spread out on a white background and held in place by metal pins, the two black marks are the positions of the start and end of the panicle rachis. This type of image is used for panicle structure, spikelet/grain counting and seed trait analyses. **(b)** Seeds spread out for the analysis of seed traits.

The input is an RGB image of a spread panicle, fixed at the center of a white background. Metal pins are used to fix the panicle onto the shooting scene (Figure 2a).

In P-TRAP, the user first has to create a project. The source images can be then imported for processing using the GUI. The project can contain one or several images. They can be processed individually or as a batch to support different workflow scales. Basic pre-processing steps can be applied on the images. Cropping and scaling processes are available and can be performed interactively using the GUI.

#### Panicle structure detection

The quantification of the panicle traits is based on the detection of the structure of the panicle followed by a conversion of the skeleton into a mathematical graph (Additional file 1). The pipeline for converting the image of the panicle to a graph can be described as follows: input image *I* is converted to grayscale and then a Gaussian blur filter with a kernel of size *kernelSize* is used to smooth the image. The smoothed image is locally thresholded by using the mean-c local thresholding approach [15], resulting in a binary image. The blurring filter is used to obtain a smooth binary image, and leads to a skeletal image containing fewer undesirable small spikes [16]. Due to variation in the brightness of the image, small holes may remain in the binary image. Unless these holes are filled, corresponding cycles may appear in the skeleton, which may cause several problems during the skeleton analysis task (Additional file 1). To solve this problem, small holes with an area ≤ *minParticle* are filled in to yield a “solid" binary image, *I*_solid_.

The *I*_solid_ image is skeletonized by the Zhang-Suen’s (ZS) thinning method [17]. A major drawback of this method is that the final skeleton may produce staircases, in which case the Holt’s staircase removal method [18] is applied. A fast lookup-based implementation of ZS method can be found in [19]. To locate the panicle skeleton in the image, all the components in the skeletal image are searched. The biggest is returned as the panicle skeleton. The skeleton is returned as a list of points (*skeletonList*) that indicate the positions of the pixels of the skeletons in the image. The *xy-origin* of the image is at the top left of the image. This list is then converted to a graph *G* which is then cleaned and refined (*G*_refined_). Cleaning is based on removing terminal edges whose length is less than a threshold *minSpike* (default value = 40 pixels, modifiable by the user). An edge is terminal if one of the vertices it connects has one and only one neighbor.

#### Panicle structure quantification

The calculation of the panicle structure traits is based on the mathematical graph produced from the panicle detection task. Quantification includes two main steps: vertices classification and graph quantification.

##### Vertex classification

**Figure 3:**
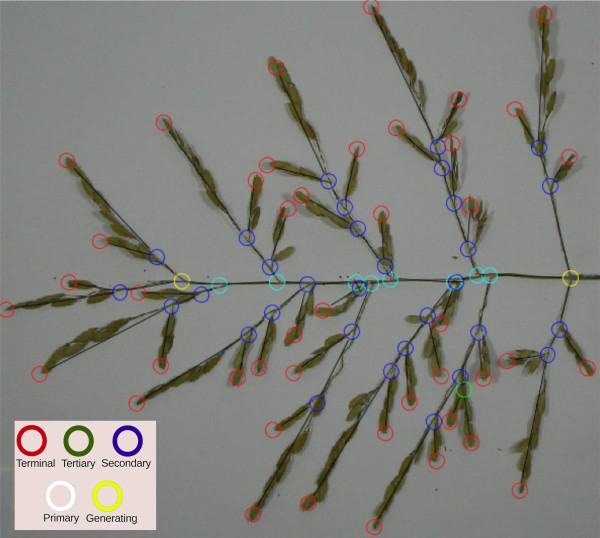
**A panicle graph superimposed on a panicle image.** The circles represent the junctions and the termination of the branches. The use of colors makes it easy for users to distinguish between different types of branches (see the inset for definitions of the colors of the circles).

**Figure 4:**
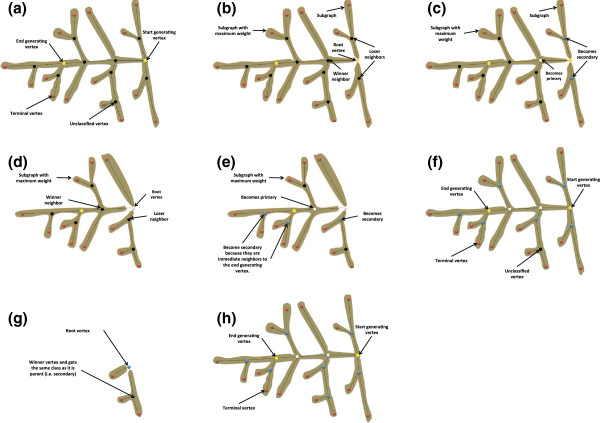
**Graph decomposition and vertex classification.** **(a)** original graph, **(b)** decomposing the graph at the start generating vertex (root), **(c)** classifying the neighbors of the start generating vertex, **(d)** decomposing the maximum weighed sub-graph at its root vertex, **(e)** classifying the vertices of the neighboring sub-graph root, **(f)** unclassified vertex in a small sub-graph, **(g)** decomposing and classifying the unclassified vertex in (f), and **(h)** in a fully classified graph.

Different classes are used to distinguish the type of graph vertices, Figure 3. The classification of vertices is explained in Figure 4. The user identifies the start and end generating vertices (yellow circles in Figure 3) of the panicle structure by using the application’s GUI, and then, each vertex of the graph is assigned to a class. The software classifies all other vertices either as terminal (red circles) or unclassified. The unclassified vertices are classified by using a breadth-first decomposition approach. Vertex classification is based on the weight of the graph. We define the weight of the graph as the product of the number of vertices and the lengths of their edges (links). The length is calculated using the Euclidean distance metric.

In the beginning, the primary vertices (white circles) are identified by decomposing the graph at the start-generating vertex (main root of the graph) into a set of subgraphs. Therefore, each neighbor of the main root is a root of a subgraph. Among the roots of the subgraphs, the one that belongs to the “heaviest” sub-graph is chosen as the“winner” vertex and then classified as primary (Figures 4b, c). The other roots are classified as secondary (*i.e.* one level lower). Similarly, the heaviest sub-graph is decomposed at its root into sub-graphs, and the new winner is classified as primary, and so on, until the end generating point is reached (Figures 4d-f).

The remaining unclassified vertices are classified in the same way as the primary ones. At each secondary vertex (that has an unclassified neighbor), its parent sub-graph is decomposed and the winner vertex is classified as secondary. The other losing vertices are classified as tertiary and so on (Figure 4g). The classification finishes when all the vertices in the graph are classified (Figure 4h). The graph terminology is defined as follows: Primary Axis (PA) is the main axis of the panicle (*i.e.* the panicle rachis), Secondary Axis (SA) is a branch attached directly to the PA (*i.e.* corresponds to a primary branch of the panicle), Tertiary Axis (TA) is an axis attached to a secondary axis (*i.e.* corresponds to a secondary branch of the panicle); Quaternary Axis (QA) is an axis that is attached to a tertiary axis (*i.e.* corresponds to a tertiary branch of the panicle).

##### Graph quantification

**Figure 5:**
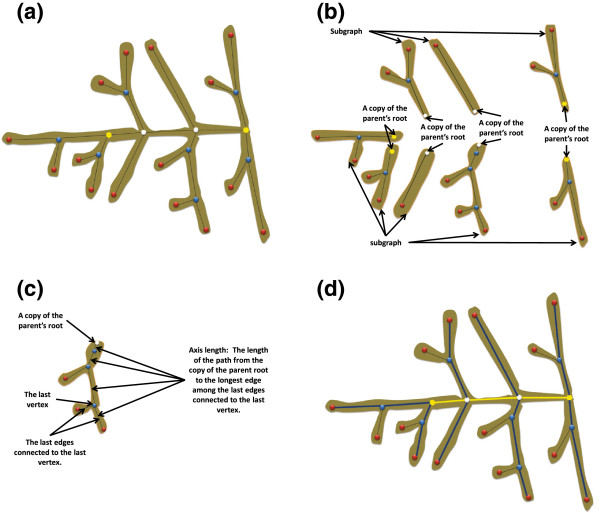
**Quantification of classified graphs.** **(a)** a classified graph, **(b)** decomposing the classified graph and adding a copy of the parent root to the sub-graphs, **(c)** calculating the length of an axis, and **(d)** yellow line: the rachis length (*PA_length*), blue lines: the lengths of the SA in the graph.

Once each vertex of the graph is classified, the panicle’s traits can be quantified. The quantification task is described in Figure 5. This task is based on the same breadth-first graph decomposition approach described earlier. A set of smaller sub-graphs is generated by the decomposition of the classified graph at its root. Each sub-graph has a copy of the root vertex where the parent graph is decomposed, a set of edges, and a set of vertices with level classes lower than that of the root. In this context, if we decompose the main graph at each primary vertex into a set of sub-graphs, each will have a primary class vertex and a secondary axis. The length of this axis is the sum of the lengths of the edges passing through the primary vertex, the secondary vertices, and the terminal vertex that is the neighbor of the last secondary vertex and has the longest edge among the other terminal neighbors (Figure 5c). Similarly, we can find the lengths of the tertiary axes in a sub-graph by decomposing it at each secondary vertex into a set of smaller sub-graphs and calculating the length of the main path in each sub-graph. This approach is used to quantify the structural traits of the panicle from the generated graph. These traits are listed in Table 1.

**Table 1:** Structural and grain related traits of a rice panicle

**Panicle structure traits**		**Spikelet (Grain) traits**	
**Trait**	**Short name**	**Trait**	**Short name**
Primary Axis - length	*PA_length*	Spikelets - number	*Sp_nb*
Primary Axis - diameter	*PA_diameter*	Spikelets - length	*Sp_length*
Secondary Axis - position	*SA_po*	Spikelets - width	*Sp_width*
Secondary Axes - number	*SA_nb*	Spikelets - area	*Sp_area*
Secondary Axis - length	*SA_length*	Spikelets - perimeter	*Sp_perimeter*
Number of Nodes	*Node_nb*	Spikelets - circularity	*Sp_circularity*
Secondary Axes Intervals Length	*SA_int*	Spikelets - compactness	*Sp_compactness*
Tertiary Axes - number	*TA_nb*	Spikelets - ellipticity	*Sp_ellipticity*
Tertiary Axes - length	*TA_length*	Aspect - ratio	*Sp_AR*
Tertiary Axis - position	*TA_po*		
Tertiary Axes Intervals Length	*TA_int*		
Quaternary Axes - number	*QA_nb*		
Quaternary Axes - length	*QA_length*		
Quaternary Axes - position	*QA_po*		
Quaternary Axes Intervals Length	*QA_int*		

Two different types of traits regarding to the panicle components. These are structural and grain related traits.

Finally, the panicle diameter or primary axis diameter (*PA_diameter*) is found by calculating the Euclidean distance map (EDM) of the *I*_solid_ binary image using an efficient algorithm described in [20]. In EDM images, each pixel has a value that defines the radius of the maximum ball (the maximum distance from this pixel to the image background). A circle with a small radius centered at the start generating point is defined as a search area. The *PA_diameter* value is then estimated as twice the square root of the maximum pixel’s value in this predefined search area.

#### Detection and quantification of grains

In the rice panicle, grains are clustered in branches, may vary in size and may overlap. These characteristics can prevent detection of the seeds on the images. For this reason, we used a granulometric approach [21]*-*[23] to find the “perfect” grain size and the other particles are then compared to this model. The same approach is used to detect seeds on the spread out panicle as well as spread out seeds. RGB images are converted to binary images in the same way as described in the section on panicle structure detection. Granulometry determines the perfect size of the mathematical morphology opening disk by estimating the range to which the correct disk size belongs and by iteratively increasing the size of the opening disk by a predefined step parameter and calculating the differences between the original and the opened images. In this work, two levels of morphological opening are performed. Formally, let *I*_binary_ be the binary image of the panicle obtained by low-passing the grayscale version of the grains’ image and applying the mean-c local thresholding method. Furthermore, let *d*_min_ ≤ *d*_*m*_ ≤ *d*_max_ be the disk size of a user-defined range. The morphologically opened version of *I*_binary_ by the structure element *d*_*m*_ can be defined in terms of particles as:

(1) $$I_{\text{binary}}\circ d_{m}=P={\left\{p_{i}\right\}}_{i=1}^{i=n_{p}}$$

where *P* is the set of particles obtained by opening *I*_binary_ by *d*_*m*_, and *n*_*P*_ is their number. To get the optimal disk size, and hence the grain size, an objective function is defined as:

(2) $$\Theta (I_{\text{binary}}\circ d_{m})=\frac{n_{P}}{\sigma (I_{\text{binary}}\circ d_{m})}\;\forall d_{m}\in \;[d_{\text{min}},d_{\text{max}}]$$

where *σ*(*I*_binary_ ∘ *d*_*m*_) is the standard deviation (STD) of the particle area.

By applying a brute-force algorithm for all disks in the range [*d*_min_,*d*_max_] with step parameter of 1, the optimal disk is the one with maximum Θ in this range. In (2) if *n*_*P*_ < *n*_min_, where *n*_min_ is a small integer, Θ is not considered. Θ is maximized, when *n*_*P*_ is big and *σ* is small, which implies an adequate disk size and consequently an appropriate grain size. Once the adequate disk size is determined, the perfect grain size is just the median of the particles in the binary image opened at this disk size. The median is chosen because it has a good gross-error toleration ratio and 50% breakdown point [24]. At this point, the first mathematical opening level is finished, with the perfect grain size $\hat{p}$ and the optimal disk size ${\hat{d}}_{1}$ identified.

At the second level, the size of the disk is smaller than in the first level ${\hat{d}}_{1}$. This ensures that the opening process removes only the thin parts of the panicle and leaves the grain particles in the branches intact. At this level, larger particles are detected in each branch by applying a morphological opening with a disk of size ${\hat{d}}_{2}=\frac{{\hat{d}}_{1}}{2}+C$, where *C* is a small constant (*C* = 3 in this work). Additionally, the concave points of each particle are calculated by examining the concavity of the particle contours as described in [25]. In this method, a circle of radius *r* with perimeter *l* is centered at each point of the contour of the particle. Let Ω(*p*_*i*_) be the set of contour points of the particle *p*_*i*_. The concavity of a contour point *ω*_*j*_ ∈ Ω(*p*_*i*_), with *j* ≤ |Ω(*p*_*i*_)|, is measured as:

(3) $$\text{concavity}(\omega_{j})=\frac{{\text{arc}}_{\text{in}(p_{i})}(\omega_{j})}{l}$$

where *p*_*i*_ is the particle and ${\text{arc}}_{\text{in}(p_{i})}$ is the length of the arc inside *p*_*i*_. In this work, a contour point is termed concave if its concavity ≥ 0.6.

#### Grain quantification

**Figure 6:**
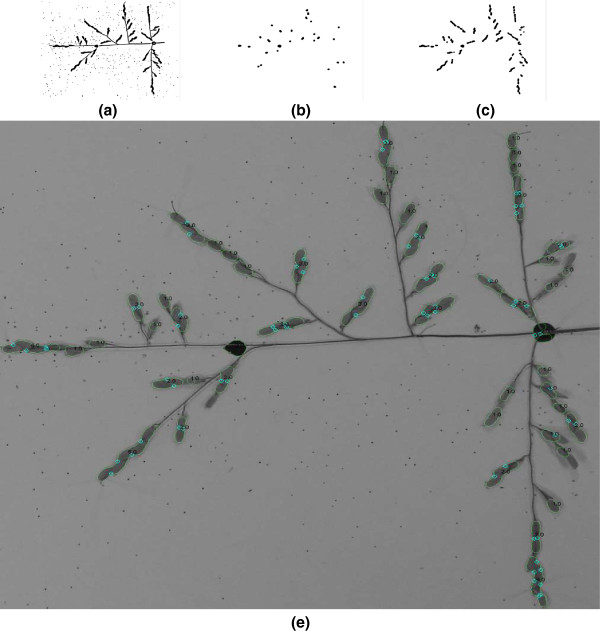
**Grain counting pipeline.** **(a)** a binary image, **(b)** the image after applying the 1^st^ mathematical opening with disk size ${\hat{d}}_{1}$, **(c)** the image after the 2^nd^ mathematical opening with disk size ${\hat{d}}_{2}$, and **(d)** results: small white circles represent the concave points.

Table 1 lists different grain traits. This section explains how they are calculated. Given the perfect grain’s area ( $\text{area}(\hat{p})$), and the area (area(*p*_*i*_)) and the number of concave points |concave(*p*_*i*_)| of each particle *p*_*i*_, the final number of grains in each particle is calculated as:

(4) $$\text{grains}(p_{i})=\alpha (\frac{\text{area}(p_{i})}{\text{area}(\hat{p})})+(1-\alpha )(\frac{\left|\text{concave}(p_{i})\right|}{2}+1)$$

where *α* ∈ [0,1] is a user defined parameter. In practice, the particle area is more accurate than the number of concave points to estimate the number of grains in the particle. For this reason, *α* is set to *α* = 0.7. The grains counting method, based on a start-to-end grain detection pipeline, is illustrated in Figure 6.

##### Calculations of grain traits

The previously described method of grain detection is designed to detect both spread out and clustered grains. However, spread out grains without the panicle can be detected without all the computation involved in the proposed method. In this context, we used a simpler pipeline (similar to the one used in [13]) just for the detection of spread out grains. Basically, given a binary image *I*_binary_, a mathematical opening with a small-predefined disk kernel can be used to remove the juts from the seeds and to smooth the contour of the grain. The grain traits listed in Table 1 are found as the following: 

• *Length*: the length of the longest line between any two points in the contour.

(5) $$\text{length}(p_{i})=\text{max}\;\Delta (\omega_{j},\omega_{k}),\;\forall \omega_{j},\omega_{k}\in \Omega (p_{i})\;\text{and}\;j\neq k$$

  Where Δ(.,.) is the Euclidean distance metric.

• *Width*: For any two points in the contour, the width is the length of the longest line perpendicular to the length’s line.

• *Area*: The number of pixels of the grain in the binary image:

(6) $$\text{area}(p_{i})=|p_{i}|$$

• *Compactness*: The relation between the area of the grain and its contour (perimeter) [26]*,*[27]. A normalized accurate compactness measure can be defined as [28]:

(7) $$\text{compactness}(p_{i})=\frac{1}{2\pi }\times \frac{\mu_{0,0}}{\mu_{2,0}+\mu_{0,2}}$$

 where *μ*_.,._ is the central moment of the specified order.

• *Ellipticity*: Measures the ellipticity of the grains [27].

(8) $$\text{ellipticity}(p_{i})=\left\{\begin{array}{rcl} 16\pi^{2}\frac{\mu_{2,0}\mu_{0,2}-\mu_{1,1}^{2}}{\mu_{0,0}^{4}} & \text{if}\frac{\mu_{2,0}\mu_{0,2}-\mu_{1,1}^{2}}{\mu_{0,0}^{4}} & \leq \frac{1}{16\pi^{2}}\\ \frac{1}{16\pi^{2}\frac{\mu_{2,0}\mu_{0,2}-\mu_{1,1}^{2}}{\mu_{0,0}^{4}}} & \text{otherwise} & \end{array}\right)$$

• *AR*: The aspect ratio is the relation between the major (length) and minor (width) axes of the grain.

(9) $$\text{AR}(p_{i})=\frac{\text{length}(p_{i})}{\text{width}(p_{i})}$$

### P-TRAP architecture and GUI

**Figure 7:**
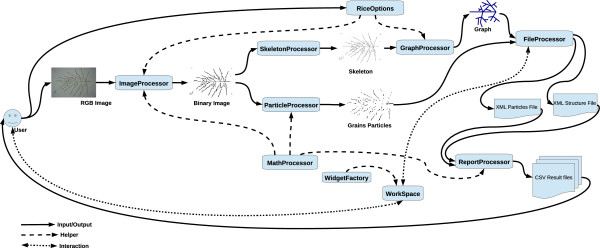
**P-TRAP architecture and the processing pipeline.** The input images and the software options are provided by the user. These images are then binarized and passed to the graph and particle processing modules to identify the structure and the grains on the panicle. The resulting graphs and particles are stored in separate XML files for visualization and additional editing. These editors are part of the *Workspace* module, which translates the XML files into editable widgets supported by the *WidgetFactory* GUI helper module. The user can easily edit these visual widgets and send the changes back to the XML files for storage. All interactions between the user and the system are performed using the *Workspace* module. The final reports are based on the contents of the XML files. The contents of these reports are stored in CSV files with different levels of detail.

**Figure 8:**
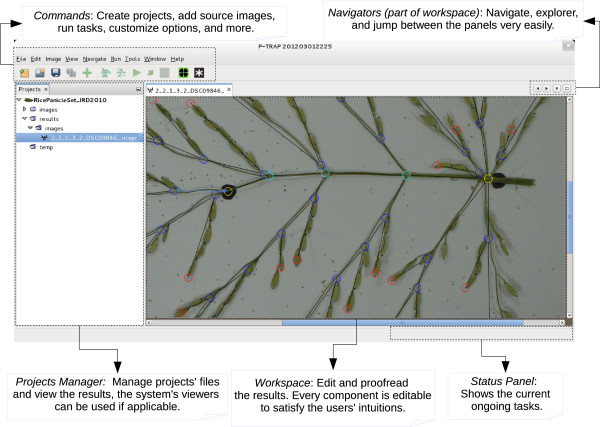
**Main GUI window areas.** *Project Manager*: the user can manipulate the project folders and their files; *Commands*: the user can run a specific process on the selected project; *Workspace*: the user can visualize and edit the selected widgets.

The system is composed of the 11 main modules listed in Table 2. Figure 7 illustrates the processing pipeline interaction between the different modules and the user interface. The main GUI window has a set of areas: *Project Manager*, *Commands*, and *WorkSpace* (Figure 8). In the *Project Manager*, the user can find the project folders, which include the source and processed images and the results sub-folders. The *Commands* area is composed of a menu and toolbar, which increases accessibility and makes it easy to find a specific command. In the *WorkSpace*, many different floating windows can be displayed at the same time. In this area, the user can review and edit the structure results in the *Structure Editor*. The same buttons are used to perform the same tasks in all windows. For instance, the user can view and edit the results of the grains in the grain editor and use the save button (Floppy icon) to save the changes (Additional file 2). The same button can be used to save the corrected result of the structure in the structure editor or the cropped image in the image editor. In addition, each editor is supplied with a context menu, keyboard-driven and mouse-driven commands. The user can correct a vertex in the structure editor by moving, deleting or connecting it. Furthermore, the class of a vertex can be changed and the application will try to adapt to the change or display an error hint if detected, Additional file 2.

**Table 2:** System main modules

**Module**	**Description**	**Type**
ImageProcessor	Core image processing tasks	Core
MathProcessor	Basic mathematical tasks required by other modules	Core
SkeletonProcessor	Skeleton-related tasks	Core
GraphProcessor	Performs the graph tasks	Core
ParitcelProcessor	Particles processing and quantification	Core
RiceProjectType	Manages the rice project folders and files	GUI
RiceOptions	Manages the application options and the algorithm parameters	GUI
WidgetFactory	Responsible for creating user friendly widgets for elegant user interactions	GUI
WorkSpace	The main module for connecting the user commands and the core modules	Link
ReportProcessor	Generates the reports	Core
FileProcessor	Manages the file system	Core

The main P-TRAP system modules differ depending on the task performed and on user visibility. The three main modules are *core*: performs an internal task, *GUI*: manages and produces visual components, and *link*: links two or more modules or the user’s commands and the core system modules.

### P-TRAP output files

The data collected from the processed images are exported in two different formats: XML and CSV. The XML format is used to store the panicle structure and the grains particles, and can be exported for other applications such as OpenAlea after conversion to the MTG (Multiscale Tree Graph) format [14]. Each analysis run produces two files: *.ricepr* and *.ricegr* for the structure and the grains, respectively. More information on the structure of the output files is available in Additional file 2. A CSV file is also generated to allow direct visualization of the results and easy transfer to spreadsheet software (*e.g.* Microsoft Excel). The results of the quantification of the panicle and grains are stored in files with two different levels of details. These CSV files are: 

• *MainTraits.csv*: contains the main general data about the panicle.

• *GrainsTraits.csv*: contains the average values of all the data on the grain’s traits.

• *AllTraits.csv*: contains detailed data on the traits of each branch.

In addition to *GrainsTraits.csv*, each image has a result file that describes each grain trait individually in the *Particles folder*.

## Results and discussion

To evaluate the accuracy of P-TRAP, 26 different images of panicles from *O. sativa*, *O. glaberrima* and *O. barthii* were tested in both structure and grain counting tasks. Images were captured using a digital camera (Sony DSC-W55) and saved in the JPEG format (Joint Photographic Experts Group). Image size was 2592 × 1944 with 72 dpi (see Additional file 3 for the 26 images tested in this work).

For grain detection and quantification, either RGB images of spread out panicles or images of spread out seeds without panicles can be used (Figure 2). Specific images of spread ou seeds have been captured using a digital camera (Canon PowerShot G12) with a size of 3648 × 2048 at 180 dpi in the JPEG format (see Additional file 4 for the images that were tested in this work).

The structure finding used by P-TRAP was evaluated and tuned by an expert using the obtained graph, and the options from the GUI, and was compared with results obtained by a field-operator created results (FO). The grain counting method was compared to two academic methods, a Lab Counting (LC) and the FO. The parameter values (in pixels) used for the tests were *minSpike* = 40, *kernelSize* = 3 × 3 and *minParticle* = 1000 for image to graph conversion. For grains counting, *d*_min_ and *d*_max_ were set to 5 and 14 pixels, respectively.

### Panicle structure

For evaluation the panicle structure, only the main manually measurable traits (length of the primary axes, number of nodes, secondary axes, and the number and length of the tertiary axes) were used for the comparison with the values obtained by P-TRAP. Table 3 summarizes first the differences between the results obtained by P-TRAP and manual measurements by comparing data before and after expert evaluation and, second, the differences between corrected P-TRAP results (*i.e.* P-TRAP data after expert evaluation) and the results obtained by the FO (Field Operator).

**Table 3:** Results of panicle structure quantification

	**P-TRAP*v* Corrected P-TRAP (%)**			**Corrected P-TRAP *v* FO (%)**		
**Trait**	**Mean**	**STD**	**Sign**	**Mean**	**STD**	**Sign**
*PA_length*	0.37%	0.87%	-	3.68%	3.68%	+
*Nodes_nb*	0.25%	1.32%	-	12.14%	12.95%	-
*SA_nb*	2.91%	1.47%	-	2.06%	7.56%	+
*SA_length*	1.05%	0.44%	+	5.46%	4.43%	+
*TA_nb*	6.09%	3.20%	+	10.60%	14.01%	+
*Sp_nb*	5.41%	3.81%	-	3.69%	4.60%	-

The percentage deviation in the processing of 26 images between 1) the P-TRAP automatic results (P-TRAP raw data) and the corrected data after expert evaluation and 2) between the corrected P-TRAP data and field operator results (FO). The factors assessed (Traits) were Rachis length (*PA_length*), Number of nodes (*Nodes_nb*), Number of secondary Axes (*SA_nb*), Average length of Secondary Axes (*SA_length*), Number of tertiary axes (*TA_nb*) and the Number of grains (*Sp_nb*). The results were calculated for the mean of differences, standard deviation (STD), and the deviation sign (Sign).

Considering all the measured traits, the average deviation between the P-TRAP automatic results and the corrected ones after expert evaluation was 2.68%, with deviations ranging from 0.25% to 6.09%(Table 3). Overall, these values indicate that P-TRAP provides robust detection and quantification of panicle structure traits with only a little post-processing required by the user. The average deviation between the corrected P-TRAP results and the FO was 6.27% (deviations ranged from 2.06% to 12.14%). The higher deviations were caused by the nodes and the number of tertiary axes (Nodes nb and TA nb respectively in Table 3). The high deviation observed in the values of *TA_nb* after expert evaluation compared to FO might be due to the fact that panicles are sometimes not properly spread out and branches overlapped (as illustrated in Additional file 5). This problem can easily be corrected by spreading the panicle out better. Furthermore, the panicle images are fixed to the background by metal pins. In some cases, the pins falsify the elongation of the branches. The difference in the number of nodes observed between corrected P-TRAP values and FO might be related to a difference in the evaluation of this feature between the software and the field operator. In the software, each secondary axis is born by an individual node. However, in some accessions, secondary axes are born by the same node and the field operator considered these as a single node.

Compared to the FO, raw P-TRAP results are more than 90% correct, which is acceptable for this difficult problem. These comparisons indicated that P-TRAP provides reliable quantification of the panicle traits as long as the panicle is properly spread out against the background.

### Number of grains and grain traits

For the grain counting evaluation, results of the P-TRAP, FO, LC and academic methods are listed in Table 4. These academic approaches are the watershed transform (WS) [29] and the Center Supported Segmentation (CSS) [30] methods (Table 4). As it is not appropriate to apply these methods directly to the original binary images (Figure 6a), they were applied to the images obtained from the second level mathematical opening, where the thin parts of the panicle are removed and only the grain clusters remain (Figure 6c).

**Table 4:** Grain counting results

**Dev.**	**P-TRAP** ***v* FO**	**P-TRAP** ***v* LC**	**WS** ***v* FO**	**WS** ***v* LC**	**CSS** ***v* FO**	**CSS** ***v* LC**
*Mean*	7.44%	6.84%	11.11%	10.84%	10.76%	10.32%
*STD*	6.21%	4.37%	8.87%	7.60%	9.13%	7.71%
*Sign*	+	+	+	+	-	-

The percentage of deviation from 26 images processing has been compared between the P-TRAP automatic results (P-TRAP raw data), the field operator (FO), Lab Counting (LC), and two well-known academic segmentation approaches: Watershed (WS) and Center Supported Segmentation (CSS). The results were calculated for the mean of differences, standard deviation (STD), and the deviation sign (Sign).

An average deviation of 7.44% (deviations ranged from 0.86% to 30.88% depending on the image with a standard deviation of 6.21% and a positive deviation sign) was observed between raw P-TRAP results and FO. The results also had a deviation of 6.84% from LC (deviations ranged from 1.80% to 26.1% with a standard deviation of 4.37% and a positive deviation sign). In contrast, the WS approach had an average deviation of 11.11% (deviations ranged from 1.85% to 39.29% with a standard deviation of 8.87% and a positive deviation). Regarding LC, WS had an average deviation of 10.84% (deviations ranged from 1.53% to 36.84% with a standard deviation of 7.60% and a negative deviation sign). The CSS method had an average deviation of 10.76% (deviations ranged from 0% to 32.35% with a standard deviation of 9.13%, and a negative deviation sign). The comparison of CSS and LC had an average deviation of 10.32% (deviations ranged from 0.48% to 26.09% with a standard deviation of 7.71%, and a positive deviation sign). P-TRAP outweighs all other methods and produces the lowest deviation and standard deviation. Which ensures the stability and accuracy for when tested to different panicles with different type of grains. The WS method is widely known to be efficient in segmenting overlapped circular shapes [30], but under-segments elliptical shapes when the overlap ratio is high [31]. Furthermore, the watershed over-segmentation problem can be clearly observed when the contour is noisy (Additional file 6). Although the CSS method is slightly better than the WS approach, in this context, it was difficult to set up an overlapping threshold for the grains that copes with the variation in the grains. The parameters used for this method were *samplingFactor* = 3, *saddleHeight* = 2, *overlappingFactor* = 0.7.

In overall, the P-TRAP method gave a good estimation of the number of grains on the images tested. It was efficient in finding the “optimal” disk size for mathematical opening. The difference between P-TRAP and FO may be a consequence of the overlapping of the grains which makes it difficult to estimate the exact number of grains (Additional file 5). Nevertheless, in contrast to other methods or applications, the used method has the advantage of detecting and counting grains directly on the panicle images. In addition, P-TRAP has different options that can be adapted to work with color and grayscale images (Additional file 2).

Finally, grain traits (Table 1) were measured using the same set of images of spread out panicles in addition to 21 images of spread out seeds (see Additional file 4). Additional file 6 presents two different examples of the detection of grain traits performed by P-TRAP from the two types of images. Averaged values of seed traits in output files result from individual seeds (*i.e.* seed clusters from spread out panicles or spread out seeds were not considered for analysis). The ability of P-TRAP to detect and quantify seeds directly on spread out panicles makes it possible to analyze seed shape traits in relation to their position in the panicle. In this context, and in comparison to the only available closed-source application (Smartgrain), both P-TRAP and Smartgrain have pros and cons. Smartgrain has two methods of segmentation, color and grayscale. The color segmentation method needs the user to define the grain and background colors. The grayscale segmentation method has a problem. It is so sensitive to variations in lightness (Additional file 6). These shortcomings can be obstacles if the source images are grayscale and have a small illumnation variation. In contrast, detection of grain traits is one of the three main tasks P-TRAP offers. Concerning segmentation, P-TRAP uses a local adaptive thresholding method (mean-c) for grayscale images. For colorful images, P-TRAP also provides an option which, like Smartgrain, asks the user to select the grain and background colors. However, as mentioned above, the main advantage of P-TRAP is that it can detect grain traits on the branches while Smartgrain does not have this feature.

### P-TRAP robustness and extensibility

Some of the requirements for P-TRAP were user-friendliness, multiple platform support, extensibility, and compatibility with other plant inflorescences with similar structure. P-TRAP also uses some general methods used for image processing. The challenge in detecting structure was to convert the panicle to a graph, and to quantify it. It has been shown that the thinning step is very important in obtaining the structure of the objects in binary images. Many applications depend on the skeletonization process to minimize the amount of data to be processed, *e.g.* Quench function [21], to extract accurate features for image matching [32], to perform image warping [33], or to analyze plant root structure [34].

The skeleton was efficient in revealing the structure of the panicle, but not enough to accurately quantify the panicle. Therefore, the panicle skeleton was converted to a mathematical graph, which was more flexible. Graphs and contours are very efficient to deal with skeletons [35]*-*[37]. In many cases, the skeleton contains small insignificant branches and cleaning has to be applied to clean the skeleton and preserve its structure at the same time. Different spike pruning approaches are available, such as the distance transform [38], the number and distribution of the maximal disk [39], branch length [40] and so on. In this work, the skeleton was initially converted to a graph and the graph was then cleaned by removing all spikes that were shorter than a threshold *minSpike* (an editable parameter in the P-TRAP options). In addition, the single-grained branches in the panicle were not significant and had to be removed. Skeleton processing, graph processing, and the quantification methods are implemented in independent modules so any improvement or extension to any of these processes can be made very easily. In addition, these modules can be reused in other projects. Concerning the detection and quantification of the grains, the challenge was to directly detect the grains on a panicle with overlapping grains and variations in size. The detection approach, particle analysis, and the central moments are implemented as modularly as possible to allow for future extensions and re-usability.

## Conclusions

P-TRAP, a freely available application for processing plant panicles is described here. This tool will be very useful for exploiting the rice diversity resources and for categorizing rice in different groups, based on inflorescence phenotyping. The tool can be used for analysis of architecture (relationship between different morphological traits), for analysis of genetics (both forward and reverse approaches) and for breeding programs. Moreover the ability of P-TRAP to detect and quantify seeds directly on spread out panicles makes it possible to analyze seed shape traits in relation to their position on the panicle (*i.e.* the apico-basal axis, primary branches *vs.* other branches).

The rice inflorescence varies widely among accessions and species in terms of branching structure and seed shape. The development of software able to automatically extract quantitative values of panicle structure and seed traits will facilitate the phenotyping of these morphological traits. A complete framework for analyzing rice panicle images is proposed in this paper. The application provides several editors for the input image, the detected structure, and the grains. The structure quantification method was compared to a manually created ground truth and the results showed an accuracy of about 90%. Grain detection and the counting method were compared to two academic methods as well as to ground truth and P-TRAP outperformed the other methods. However, the application, especially the method for detecting the skeleton of the panicle and converting it to a graph has one main shortcoming. It may not correctly detect overlapped branches, and in some cases, this may require some manual post processing to correct the structure. However, this problem can be minimized by carefully spreading out the panicle on the background. On the other hand, P-TRAP can efficiently deal with different rice panicles regardless of their size or complexity. Finally, the P-TRAP processing pipeline is implemented in a highly modular environment and developers can easily improve the application. A further important feature of P-TRAP is that the data are stored in XML files, which can be used in other applications such as OpenAlea, a platform dedicated to plant architecture.

In addition to P-TRAP’s fully featured GUI, some other features are: 

• Free open source application

• Platform-independent

• Written on top of a well-known modular platform (Netbeans Platform)

• User-friendly interface

• Allows the users to save the processed image.

The application comes with different installers that are available at the application’s website. The source code and a sample project can be found in Additional files 7 and 8, respectively. For details of the GUI features, the reader should refer to the user manual in Additional file 2.

## Availability and requirements

**Project name:** P-TRAP

**Project home page:**http://bioinfo.mpl.ird.fr/index.php?option=com_content&view=article&id=102&Itemid=2.Several video tutorials can be found at this URL.

**Operating system(s):** Platform independent

**Programming language:** Java

**Other requirements:** JRE ≥1.6 to run the application. To compile the source code, the Netbeans Platform ≥ V.7.3 IDE, Java Matrix Package (JAMA) ≥ V.1.0.2 and Java Advanced Imaging (JAI) ≥ V.1.3 libraries are needed.

**License:** GPL V3

**Any restrictions to use by non-academics:** As specified by GPL V3 license.

## Abbreviations

CSV: Comma separated values; XML: EXtensible markup language; MTG: Multiscale tree graph; CSS: Center supported segmentation; FO: Field operator result; P-TRAP: Panicle tRAits phenotyping; PASTAR: Panicle structure analyzer for rice; PA: Primary axe; Pb: Primary branch; SA: Secondary axe; Sb: Secondary branch; Sp: Spikelet; TA: Tertiary axe; Tb: Tertiary branch; QA: Quaternary branch; JAMA: Java matrix package; JAI: Java advanced imaging.

## Competing interests

The authors declare that they have no competing interests.

## Authors’ contributions

All authors contributed to the development of P-TRAP HA, HRS, and SJ managed and organized the project. HA, SJ, AG, and ML provided the biological background, the testing samples and tested the results during development. FA, and HA, designed and implemented the algorithms and tested the software. PL contributed to local installation, maintenance and evaluation of P-TRAP software. All the authors read and approved the final manuscript.

## Supplementary Material

Additional file 1 **Skeleton conversion to mathematical graph.** The technical description of the algorithm for converting the skeleton into a graph. Click here for file

Additional file 2 **User manual of P-TRAP.** The description of the software and a set of examples of how the user can install and use the application. Click here for file

Additional file 3 **26 images of spread out panicles.** A set of images of spread out panicles used to test the application for the detection of the structure, counting the grains or spikelets and for the detection of grain traits. Click here for file

Additional file 4 **21 images of spread out seeds.** A set of images of spread out grains used to test the application for the detection of grain traits. Click here for file

Additional file 5 **Example of overlapping grains.** Samples with extremely overlapped grains. Click here for file

Additional file 6 **Sample images processed by P-TRAP and by other approaches.** Several different images processed by P-TRAP and by other approaches. Click here for file

Additional file 7 **P-TRAP source code.** The Netbeans project that contains the source code of the application. Click here for file

Additional file 8 **Test data for the P-TRAP software.** A complete P-TRAP project, can be used in the application for tests. Click here for file
