# Supplementary material for: P-TRAP: a Panicle Trait Phenotyping tool
Source: BMC Plant Biol. 2013 Aug 29;13:122. doi: 10.1186/1471-2229-13-122 (PMC3848748; doi:10.1186/1471-2229-13-122)
Supplement: Additional file 1 — Skeleton conversion to mathematical graph. The technical description of the algorithm for converting the skeleton into a graph. [file 1471-2229-13-122-S1.zip › Additional file 1/image2graph Algorithm.pdf]

P-TRAP: a Rice Panicle Traits Phenotyping Tool Based on 2D Image

I. Panicle Image to Graph

The algorithm of converting the rice panicle image to a graph is shown in Algorithm I.1. In this algorithm, the image is converted to grayscale in order to be locally thresholded by using mean-c local threshold approach [4]. Before applying the local threshold, a Gaussian blur filter with kernel of size k is used to smooth the image [8], so the skeletal image contains less undesirable small spikes. However, in these images, median filter performs better than the blurring filter, but it cuts the branches wherever the branch is thin. The blurred image is then locally thresholded to obtain a binary image.

Due to lightness variation in the image, small holes may remain in the binary image. A naive solution of this problem is to fill the small ones to yield a "sold" binary image. This is done by flood filling all holes binary image and then removing the holes that have size greater than m. Without filling these small holes, a small cycles are likely to appear in the skeleton which may cause several problems during skeleton analysis, see Figure 1.

The thinning algorithm used is Zhang-Suen algorithm [12]. It is widely used and proved to work well in case of lines' skeletonization. A major drawback of this method is that the final skeleton may produce some staircases, therefore the Holt's staircase removal method [5]is applied. To locate the panicle skeleton in the image, all component in the skeletal image are found. The biggest component is returned as the panicle skeleton. This skeleton is returned as a list of points that indicate to the positions of the skeleton pixels in the image. This is denoted as GetMaxConnectedCompnent(.) function in the algorithm.

Algorithm I.1: IMAGETOGRAPH(I, l, k, m)

$I_{\text{grayscale}} \leftarrow \text{Grayscale}(I)$
$I_{\text{blurred}} \leftarrow \text{GaussianBlur}(I_{\text{grayscale}}, k)$
$I_{\text{binary}} \leftarrow \text{LocalThresholding}(I_{\text{blurred}})$
$I_{\text{sold}} \leftarrow \text{SmallHolesFilling}(I_{\text{binary}}, m)$
$I_{\text{skeleton}} \leftarrow \text{ZhangSuen}(I_{\text{sold}})$
$I_{\text{cskeleton}} \leftarrow \text{Holtstair}(I_{\text{skeleton}})$
$C \leftarrow \text{GetMaxConnectedCompnent}(I_{\text{cskeleton}})$
$G \leftarrow \text{BuildGraph}(C, C[0], C[0])$
$G_{\text{refined}} \leftarrow \text{RefineGraph}(G, l)$
return (G_{refined})

So far, most of the work is widely found in many applications that depend on the thinning algorithm, either to minimize the amount of data to be processed, see Quench function concept in [10] or to extract accurate features for image matching [9] or even for image warping [11]. This work describes how the skeleton can be converted to a graph and how the rice panicle structure can be found in the graph. Nevertheless, this is not new and can be found in the literature [3], [7], [6], the way that

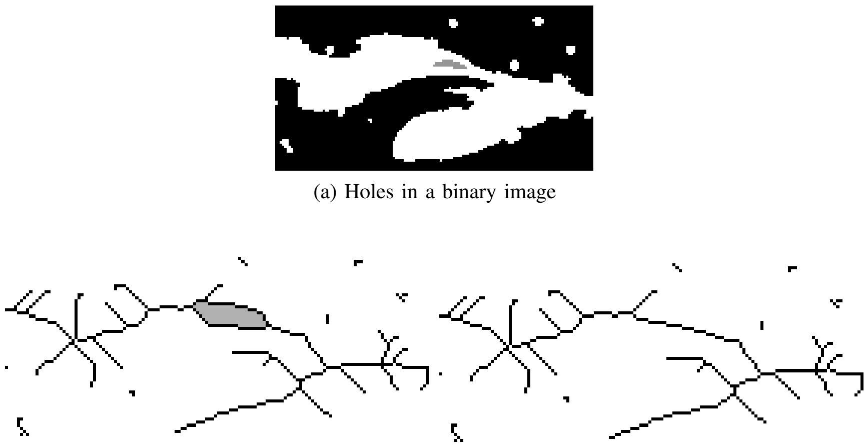

(a) Holes in a binary image

(b) Cycles in the skeleton of the image

(c) Skeleton after filling the holes

Figure 1: Holes in the binary image and corresponding cycles in the skeleton.

we clean the graph is simple and is efficient to produce a clear and an easy to process graph that describes the rice panicle accurately. However, different algorithms of spike pruning are available, but removing spikes from the graph is advantageous comparing to runs or matrices representation of the skeleton. The skeleton pruning can be done by using distance transform [1], maximal disks' numbers and distribution [2], branch length and so on. In this work, the pruning is applied on the graph, that takes the advantage of the graph representation in the one hand, and on the other hand to clean the skeleton. In fact the use of a threshold to remove the small branches are not suggested [2], but in this particular case it is efficient to clean the graph.

In Algorithm I.2 the graph is build out of the *skeleton* list recursively. In the beginning, the skeleton is traced starting by any point. In each iteration, two points are traced: current and last. If a current's neighbor is not visited yet (a global $Visited$ list is used to keep track of the visited points) and is a junction (has ≥ 3 neighbors) or an end point (has exactly 1 neighbor) an edge is created in the graph global variable G. The repeated edges are ignored to insure uniqueness of the edges in the graph. This scheme is recursively repeated until all points are visited.

Algorithm I.2: BUILDGRAPH($skeleton, current, last$)

$neighbors \leftarrow$ Get3by3Neighbors($current$)
for all $p \in neighbors$
do {
  if $Visited$.contains(p_i) $=$ **false**
  then {
    $Visited$.add(p)
    if (isJunction(p) **or** isEndpoint(p))
    then {
      if (G.hasEdge($p, last$) $=$ **false**)
        then G.addEdge($p, last$)
      $last \leftarrow p$
    }
    BuildGraph($skeleton, p, last$)
  }
}
return (G)

The direct conversion from skeleton to graph produces a noisy graph. To clean up the graph, short spikes have to be removed. In order to do that, a length threshold l is used to define the length of the maximum length of a spike. The algorithm of refining the graph is shown in Algorithm I.3. This algorithm has two functions: RemoveTransitions(.) and RemoveSpikes(.).

Algorithm I.3: REFINEGRAPH(G, l)

$removed \leftarrow 1$
while $removed > 0$
do {
  G.RemoveSpikes(l)
  $removed \leftarrow G$.RemoveTransitions()
}
return (G)

Once spikes that have length $\leq l$ are removed by RemoveSpikes(.) function, transition vertices are then removed by RemoveTransitions(.) function. A transition vertex is a vertex that has exactly 2 neighbors.

REFERENCES

[1] D. Attali, G. di Baja, and E. Thiel. Pruning discrete and semicontinuous skeletons. In C. Braccini, L. DeFloriani, and G. Vernazza, editors, *Image Analysis and Processing*, volume 974 of *Lecture Notes in Computer Science*, pages 488–493. Springer Berlin - Heidelberg, 1995. 10.1007/3-540-60298-4-303.

[2] D. Attali, S. G. di Baja, and E. Thiel. Skeleton simplification through non significant branch removal. *Image Processing and Communication*, 3(3-4):63–72, 1997.

[3] X. Bai, L. Latecki, and W.-Y. Liu. Skeleton pruning by contour partitioning with discrete curve evolution. *Pattern Analysis and Machine Intelligence, IEEE Transactions on*, 29(3):449 –462, march 2007.

[4] W. A. . W. E. Fisher R., Perkins S. *Hypermedia Image Processing Reference*. J. Wiley & Sons Publishing, 1996.

[5] C. M. Holt, A. Stewart, M. Clint, and R. H. Perrott. An improved parallel thinning algorithm. *Commun. ACM*, 30(2):156–160, Feb. 1987.

[6] U. Montanari. Continuous skeletons from digitized images. *J. ACM*, 16(4):534–549, Oct. 1969.

[7] R. Ogniewicz and O. Kübler. Hierarchic voronoi skeletons. *Pattern Recognition*, 28(3):343 – 359, 1995.

[8] S. M. Pizer, W. R. Oliver, and S. H. Bloomberg. Hierarchical shape description via the multiresolution symmetric axis transform. *Pattern Analysis and Machine Intelligence, IEEE Transactions on*, PAMI-9(4):505 –511, july 1987.

[9] H. Sundar, D. Silver, N. Gagvani, and S. Dickinson. Skeleton based shape matching and retrieval. In *Shape Modeling International, 2003*, pages 130 – 139, may 2003.

[10] L. Vincent. Granulometries and opening trees. *Fundam. Inf.*, 41(1-2):57–90, Jan. 2000.

[11] G. Wolberg. Skeleton-based image warping. *The Visual Computer*, 5:95–108, 1989. 10.1007/BF01901485.

[12] T. Y. Zhang and C. Y. Suen. A fast parallel algorithm for thinning digital patterns. *Commun. ACM*, 27(3):236–239, Mar. 1984.
