# Supplementary material for: P-TRAP: a Panicle Trait Phenotyping tool
Source: BMC Plant Biol. 2013 Aug 29;13:122. doi: 10.1186/1471-2229-13-122 (PMC3848748; doi:10.1186/1471-2229-13-122)
Supplement: Additional file 2 — User manual of P-TRAP. The description of the software and a set of examples of how the user can install and use the application. [file 1471-2229-13-122-S2.pdf]

P-TRAP: Panicle Traits Phenotyping Tool

User Manual

Faroq AL-Tam[1]: faroq.al.tam@gmail.com, Helene Adam[2]: adam.helene@gmail.com,
António dos Anjos[4,5]: antoniodosanjos@gmail.com, Mathias Lorieux[3]: mathias.lorieux@ird.fr,
,Pierre Larmande [2]:Pierre.Larmande@ird.fr, Alain Ghesquière[2]: alain.ghesquiere@ird.fr,
Stephane Jouannic[2]: stephane.jouannic@ird.fr and Hamid Reza Shahbazkia [1]: shahbazkia@gmail.com

1- DEEI-FCT Universidade do Algarve, 8005-139 Faro, Portugal
2- IRD, UMR DIADE, Genome and Development of Rice group, 911 avenue agroplois, 34000 Montpellier, France
3- CIAT, Agrobiodiversity and Biotechnology Project, Cali, Colombia
4- DCT, ISMAT - Instituto Superior Manuel Teixeira Gomes 8500-508 Portimão, Portugal
5- CCMAR-CIMAR Laboratório Associado Universidade do Algarve 8005-139 Faro, Portugal
contact: *ptrap.ird@gmail.com*

Application website:
http://bioinfo.mpl.ird.fr/index.php?option=com_content&view=article&id=67&Itemid=78

2013

Contents

Contents 3

List of Figures 5

1 Installation and Main Components 7

1 Introduction 7
1.1 System Requirements 7
1.2 Installation 8
1.2.1 Linux 8
1.2.2 Windows 8
1.3 The Main Window 8
1.3.1 Commands 10
1.3.2 Project Manager 10
1.3.3 Workspace 10
2 Files 13
2.1 Images 13
2.2 Structure and Grains Files 15

2 Working with P-TRAP 17

1 Introduction 17
2 Tasks 17
2.1 Getting Started 17
2.2 Adding Images to P-TRAP 19

2.3 Working with Images 20
2.4 View the Source Images 20
2.5 Crop an Image 22
2.6 Scaling Images 22
2.7 Working with the Panicle Structure 23
2.7.1 The Structure of a Single Image 23
2.7.2 The Structure of Multiple Images 23
2.7.3 View and Edit the Structure 24
2.8 Working with the Grains 25
2.8.1 Detecting the Grains in a Single Image 25
2.8.2 Detecting the Grains in Multiple Images 28
2.8.3 Color-Segmentaion for grains detection 28
2.8.4 View/Edit the Grains Files 28
2.9 Collecting the Reports 29
2.10 Options 31
2.10.1 Defining the Scale 32
2.10.2 Image's Background 32
3 Troubleshooting 33

A XML Files 35
1 Structure XML File 35

List of Figures

Installation and Main Components **7**

1 The Linux installation window of P-TRAP 8

2 The Windows installation window of P-TRAP 9

3 The main window of P-TRAP. 9

4 The toolbar of the main window. 10

5 P-TRAP project folders. 11

6 The images editor. 11

7 The panicle structure editor. 12

8 The grains editor. 13

9 The grains editor can be also used for editing separated grains. 14

10 The files that P-TRAP uses/produces. 14

Working with P-TRAP **17**

1 The *New Project* dialog: Select the project type 18

2 The *New Project* dialog: Name and location of the project 18

3 A new project named "RicePanicleSet_IRD2010" is created 19

4 The *Open* dialog: Add images to the project 19

5 New images are imported to the project. 20

6 View/edit image in the Image Editor. 21

7 Zoom in/out in the scene in the Image Editor 21

8 Image editor menu. 22

9 Scale images in a selected project 22
10 Structure detection notifications 23
11 A structure file generated after the image processed 24
12 Circles context menu 25
12 Defining the start and end generating circles 27
13 An error hint generated by the application to allow the user locate the error easily. . 27
14 A grains file generated from processing a panicle image. 28
15 The anchors of the grains. 29
16 Grains number correction. 30
17 The panicle options dialog 31
18 Defining the scale in image. 33

Chapter 1

Installation and Main Components

1 Introduction

P-TRAP is an application for analyzing plant panicle images. It is a cross-platform application developed in Java and designed on top of Netbeans Platform 7.1. It allows the user to process the images in a project-based approach. The images to be processed are added to a project and when analyzed, the intermediary results are saved in separate files. The final results can be collected in master and details files. Additionally, The panicle structure and the grains are stored in XML files which can be exported to be used by other applications. Besides this manual, several video tutorials can be found in the application website.

1.1 System Requirements

1. System: Platform-independent

2. Processor: Core 2 Due or higher

3. Memory: 2 GB or more

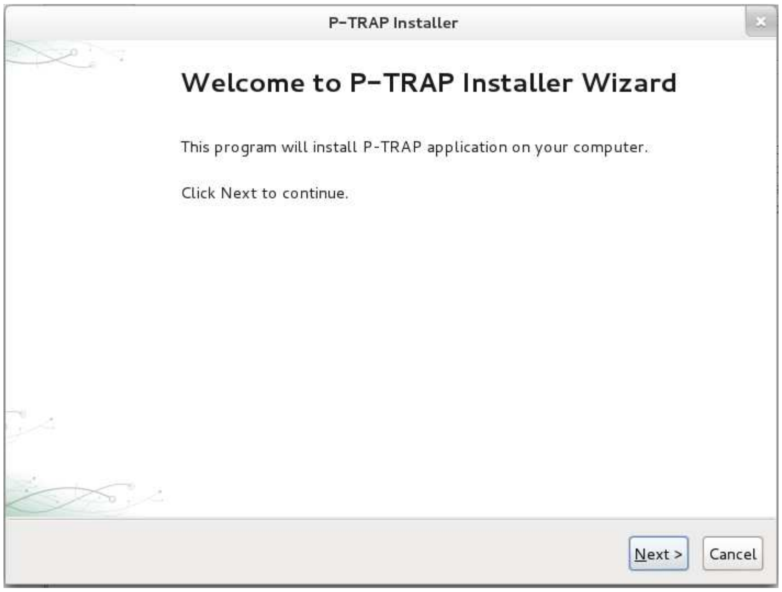

Figure 1: The Linux installation window of P-TRAP

1.2 Installation

1.2.1 Linux

Open the terminal, go to the folder where the *grain-linux.sh* is located and type: *sh grain-linux.sh* The installer window will then appear, Figure 1. Once you press *Next* button, the installer will ask you where to put the application and its shortcuts.

1.2.2 Windows

In Windows operating system, locate the file *grain-windows.exe* and open it. The installer, Figure 2, will open, click *Next* button and follow the on-screen simple instructions.

1.3 The Main Window

The main window of P-TRAP is shown in Figure 3. This window has three main areas as explained in this figure.

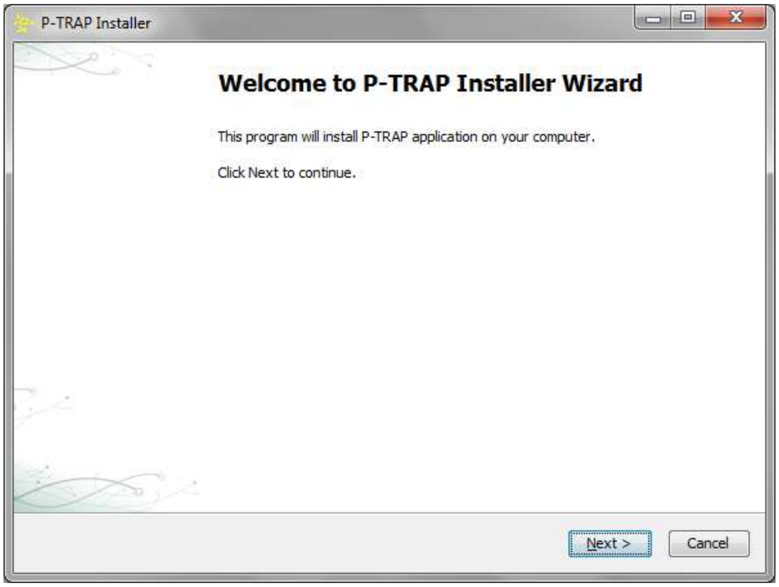

Figure 2: The Windows installation window of P-TRAP

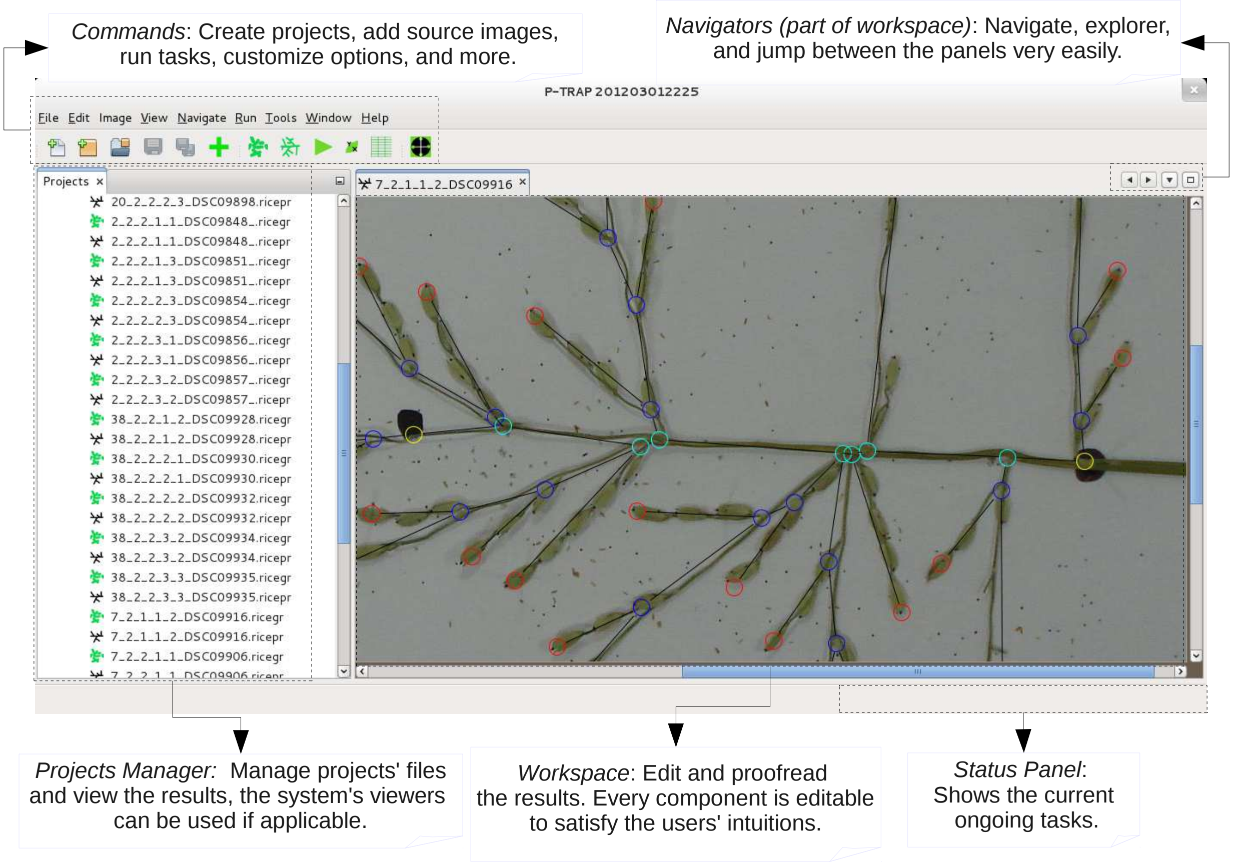

Figure 3: The main window of P-TRAP.

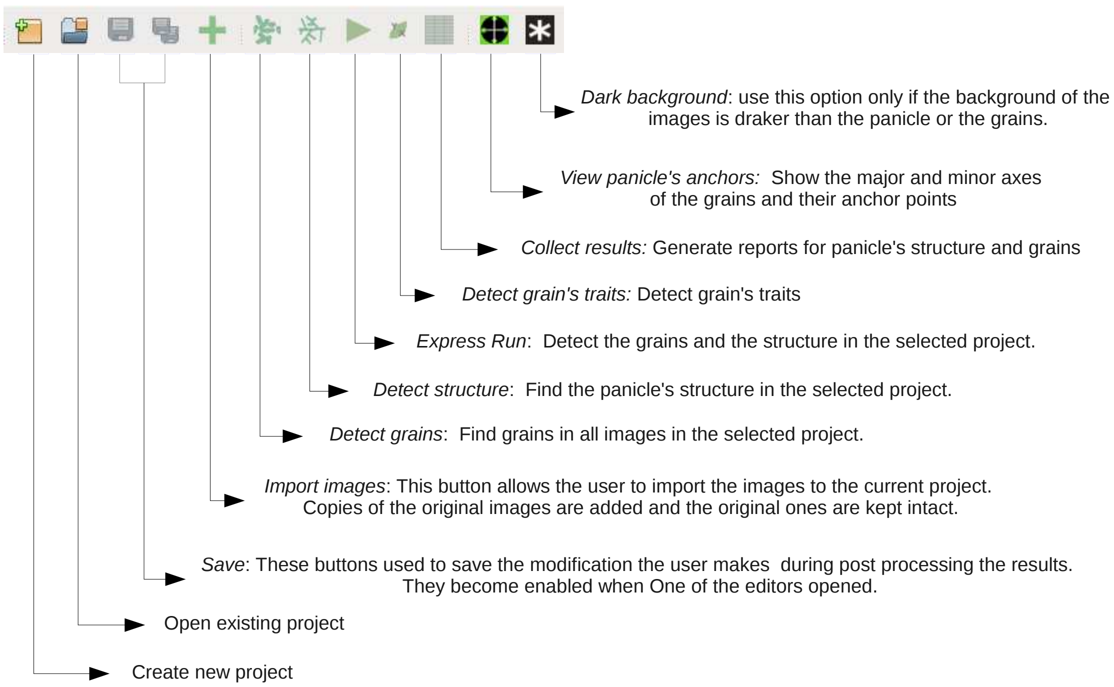

Figure 4: The toolbar of the main window.

1.3.1 Commands

In the *commands* area, the tool bar has several buttons with different functionalities explained in Figure 4

1.3.2 Project Manager

In this area, Figure 5, all files and folders in the project can be dealt with very easily. A context menu (right-clicked menu), Figure 5b, can be used to manage the files.

1.3.3 Workspace

In Workspace, the user can view and edit: images, structure and grains' results. In this area, specialized editors for the images, structure and grains are available. These editors share the same commands for zooming and moving the scene[1]. The *ImageEditor*, Figure 6, allows the user to select the region-of-interest and crop the image, Figure 6.

[1] In the editor, the scene is the entire area where the user can work

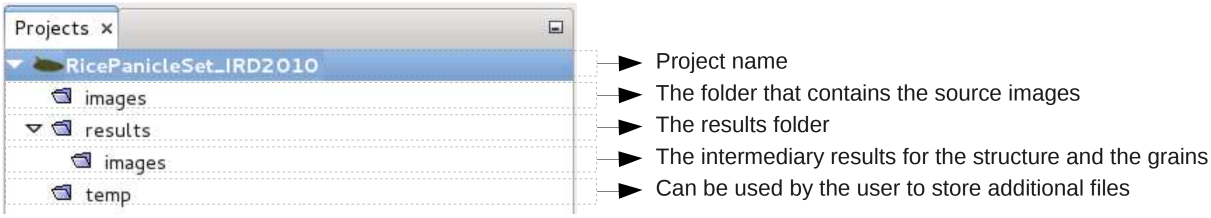

(a) Project components

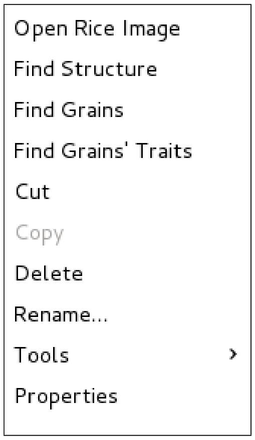

(b) Project components menu

Figure 5: P-TRAP project folders.

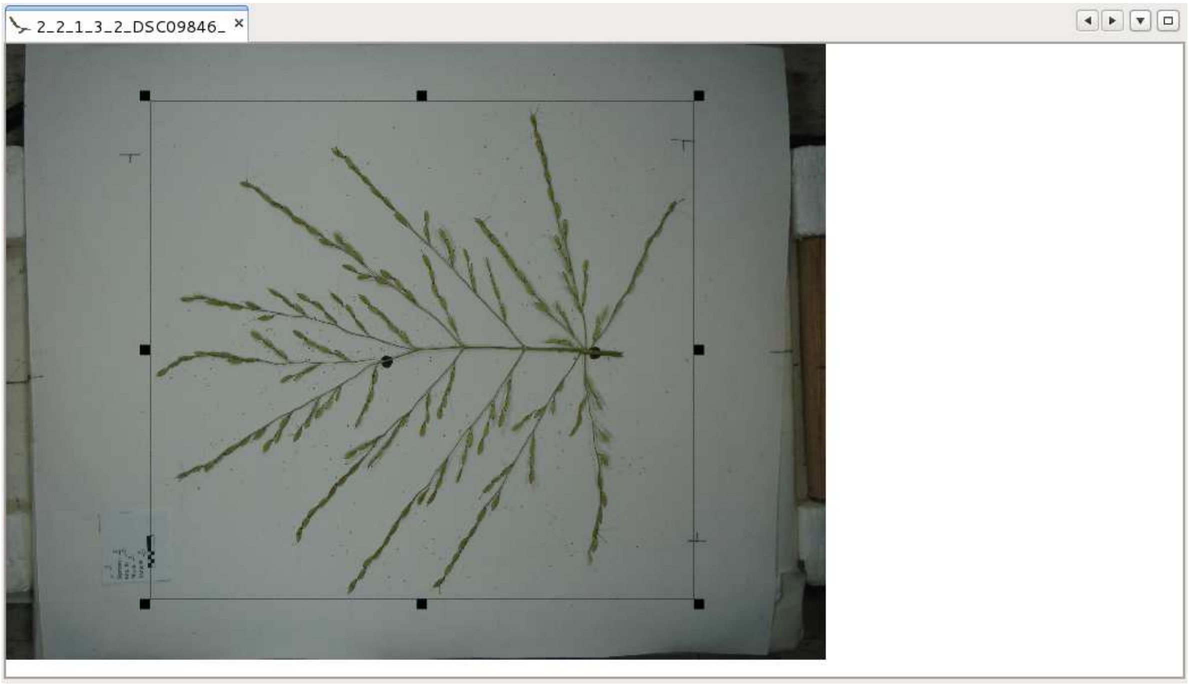

Figure 6: The images editor.

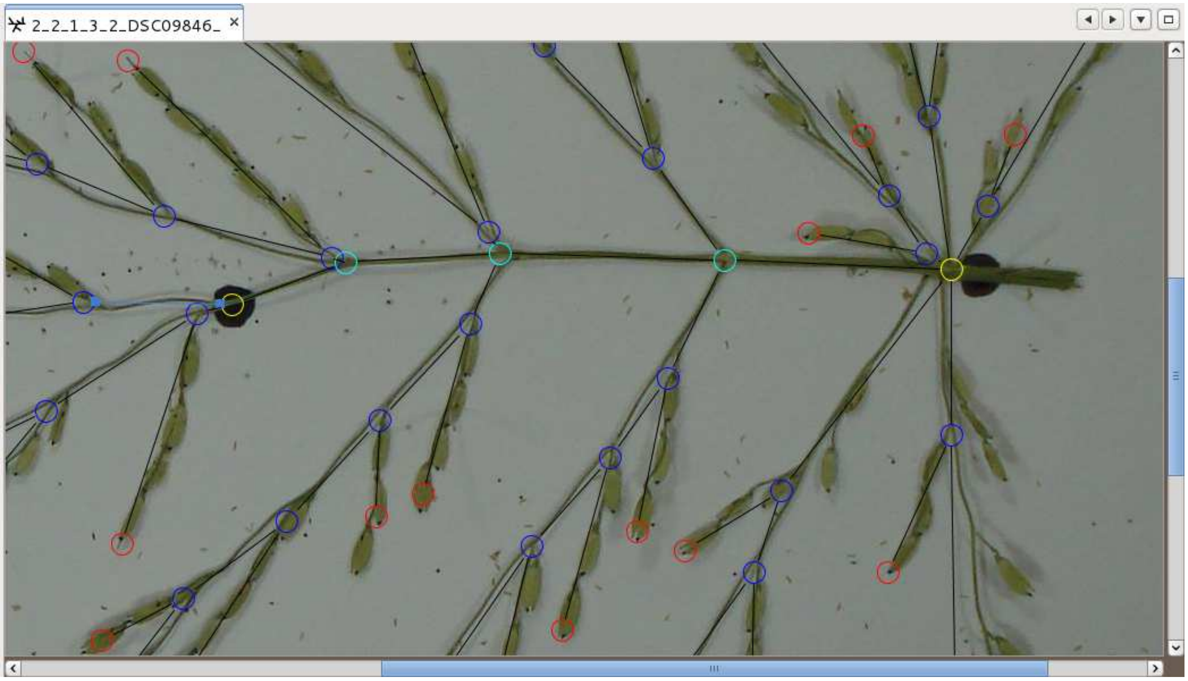

(a) Structure editor

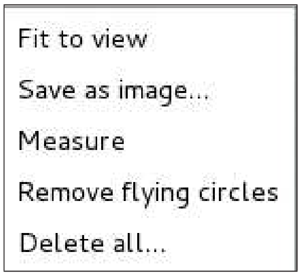

(b) Structure editor menu

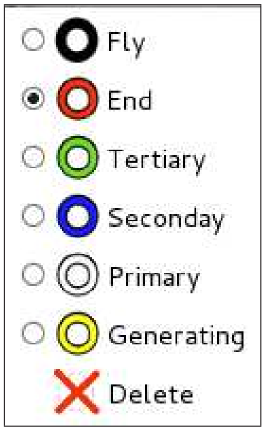

(c) Structure components menu

Figure 7: The panicle structure editor.

In order to crop the image, first select the area where the panicle is, then click *save* . button. By using the handles of the cropping frame the user can easily choose the exact area to be crop out.

This editor is used for both editing the grains detected on the branches of the panicle or the grains used for detecting grains' traits, Figure 9.

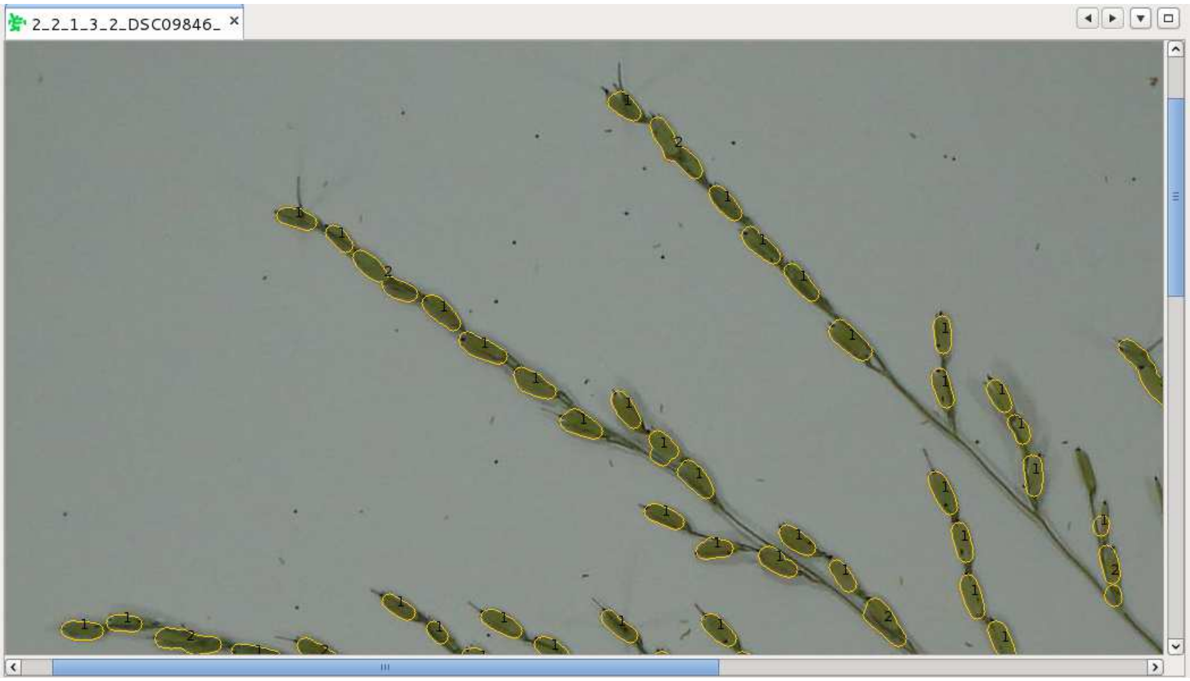

(a) Grain editor

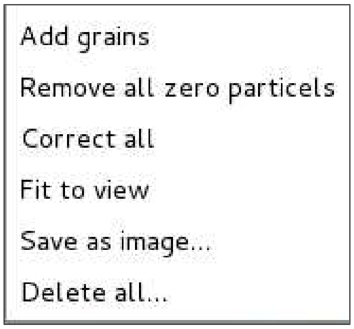

(b) Grain editor menu

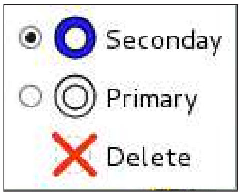

(c) Grains menu

Figure 8: The grains editor.

2 Files

In this section, the files that the application works with and generates are described. These files are described and shown in Figure 10.

2.1 Images

The application works on most of the well-known image formates. It supports both the colored and grayscale images. For better performance, it is better to scale the images as we will see in

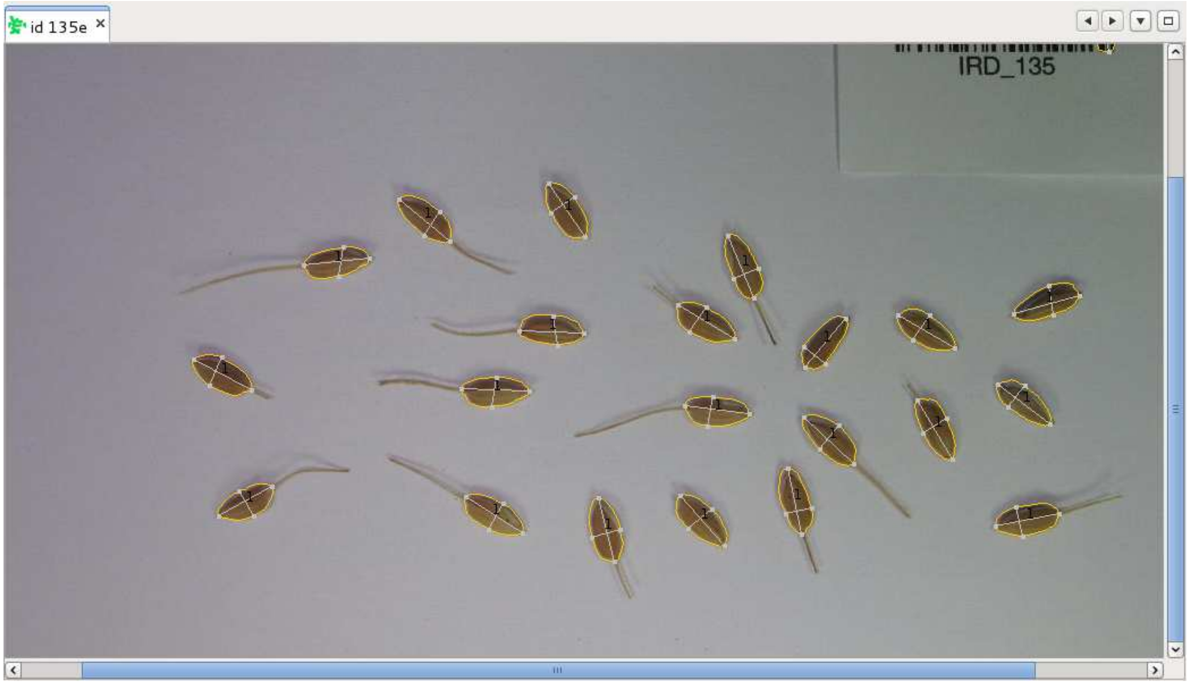

Figure 9: The grains editor can be also used for editing separated grains.

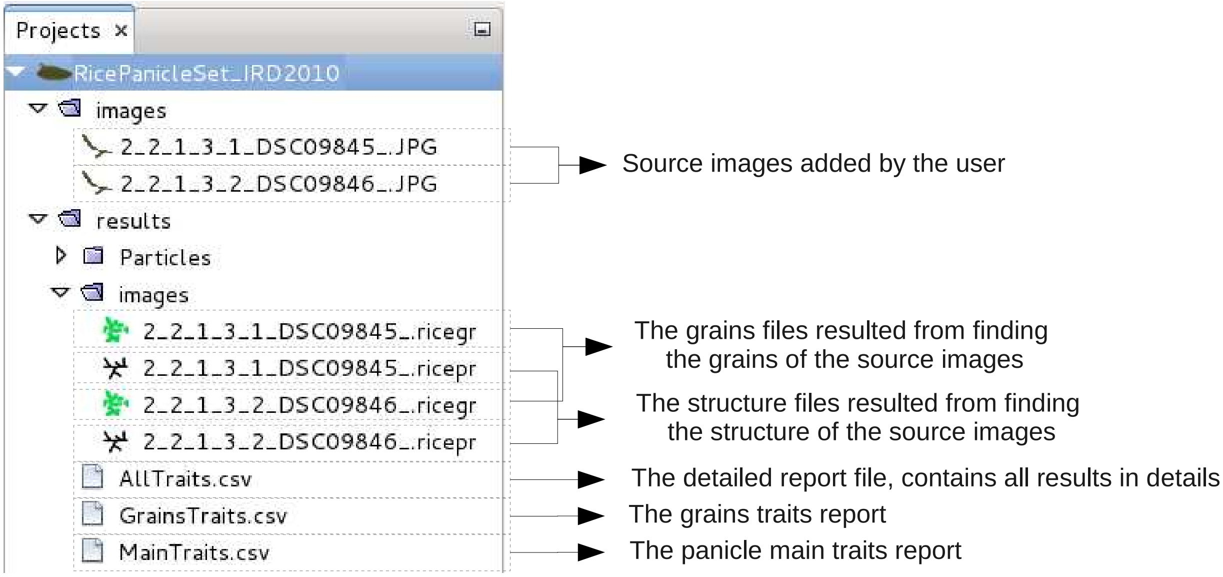

Figure 10: The files that P-TRAP uses/produces.

Section 2.6.

2.2 Structure and Grains Files

The structure and the grains of the panicle are stored in XML files. These files can be used by other applications. If you are interested to use them in your application please see Appendix A. In fact, the P-TRAP's editors described earlier uses these files to visualize the results and allow the user to visually post-process them. These files are shown in Figure 10.

Chapter 2

Working with P-TRAP

1 Introduction

This chapter provides a step by step illustration for using P-TRAP. It is divided to a set of examples. In each one a specific task is addressed and explained. The work with P-TRAP is very simple. The idea is to create a project that contains several images with the same scale and background intensity (*i.e* dark or light background). Then process the project with options suitable for its images.

2 Tasks

2.1 Getting Started

In this section, the creation of a new project is explained. To create a project do the following:-

1. Open the application
2. Click on the *New Project* 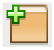 button in the tool bar
3. In the *New Project* wizard, Figure 1, choose *Rice Processing Project* and click *Next* button.
4. Specify a name and where the project will be stored, Figure 2.

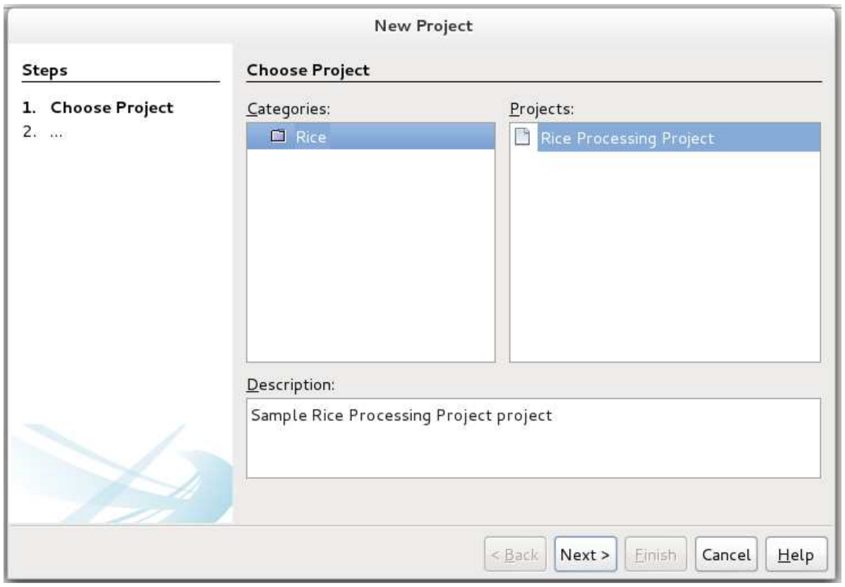

Figure 1: The *New Project* dialog: Select the project type

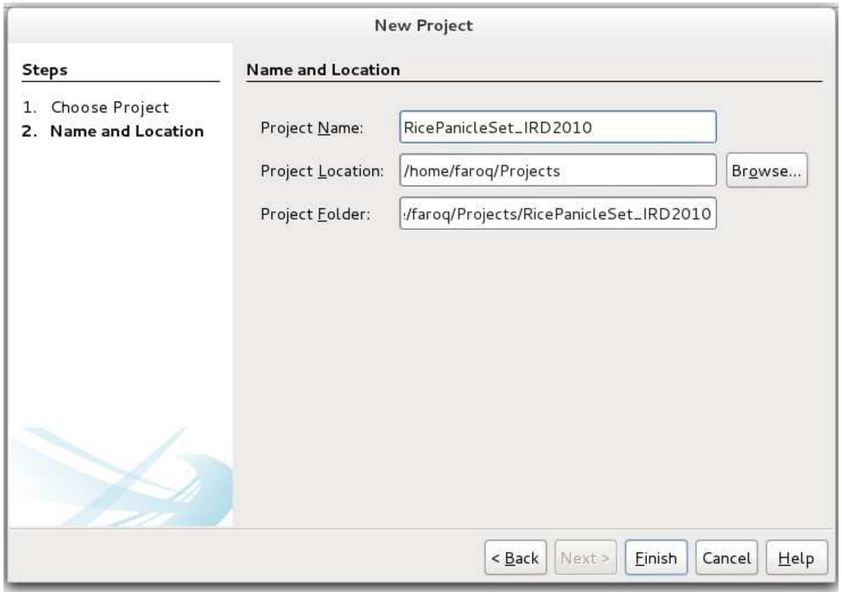

Figure 2: The *New Project* dialog: Name and location of the project

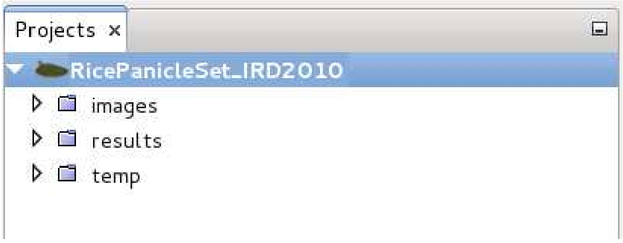

Figure 3: A new project named "RicePanicleSet_IRD2010" is created

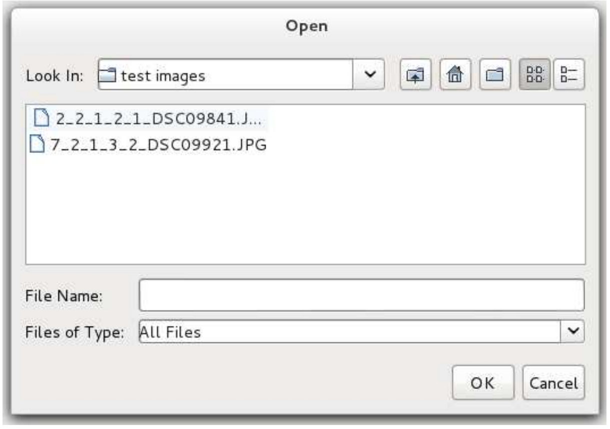

Figure 4: The *Open* dialog: Add images to the project

5. Click *Finish*.

A new project will be then created as shown in Figure 3.

2.2 Adding Images to P-TRAP

In this step one or more images will be imported to the project. To do that, follow these steps:-

1. Click on the *Import Images* button in the toolbar
2. In the *Open* dialog, Figure 4, locate your images files and click *OK*. You can choose single or multiple files to add to the project. Hold *ctrl* or *shift* keys during clicking on the files to select/deselect multiple file. To select all files, press *ctrl+A* key combination.
3. Click *OK*, the image will be added to the *images* folder in the project, Figure 5.
4. Click finish.

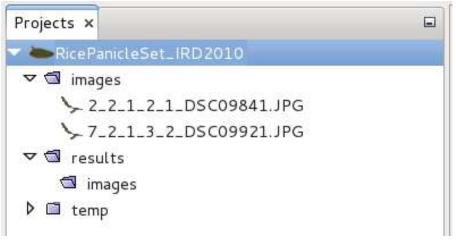

Figure 5: New images are imported to the project.

It is important to note, at this point P-TRAP does not uses the original images. Instead, it imports a copy of the original ones. So the original images are kept intact.

2.3 Working with Images

This step has some optional tasks if your images do not have huge size (we consider more than 1024×1024 as a big image) and do not have labels or additional hand-made marks.

2.4 View the Source Images

To view an image you can do one of the following:-

1. Double click on the image file in the project, or

2. Right click on the image, a context pop-up menu appears, choose *Open Rice Image*, Figure 5b.

3. The *Image Editor* will then open the image and let you tweak it, Figure 6.

4. Hold the *ctrl+mouse wheel* to zoom in/out the scene, Figure 7. Please note that, this key/mouse combination can be used in all editors in P-TRAP to perform the zoom in/out task.

Besides, this editor has a popup menu to select the color of the grain and background. This is be used in grain detection task later on this chapter.

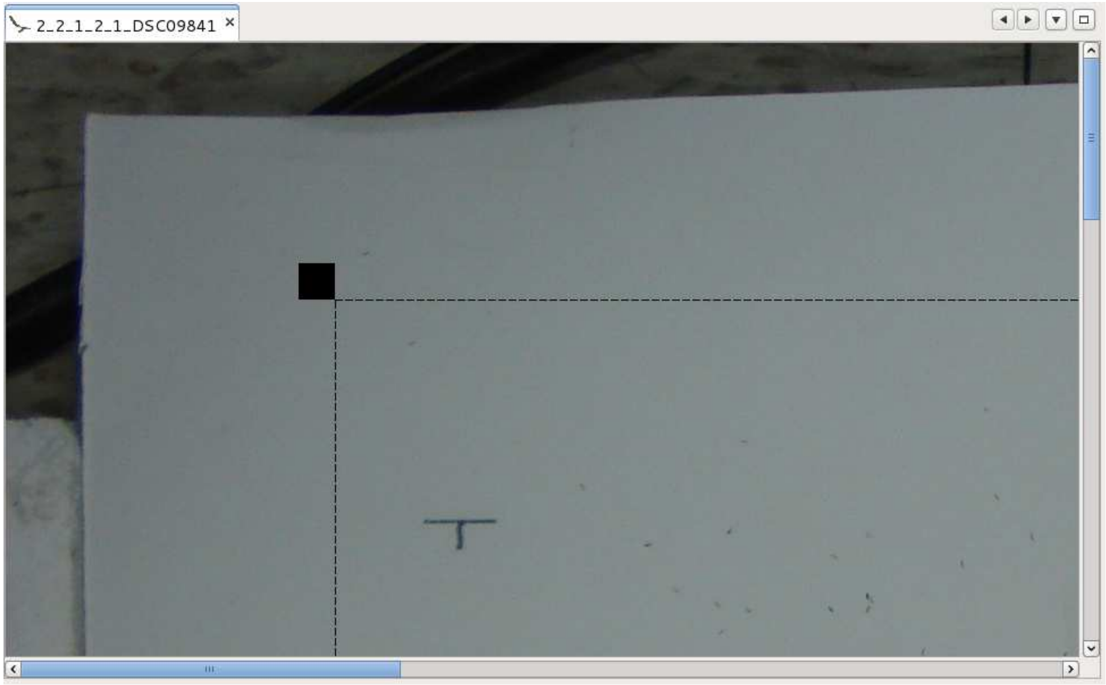

Figure 6: View/edit image in the Image Editor.

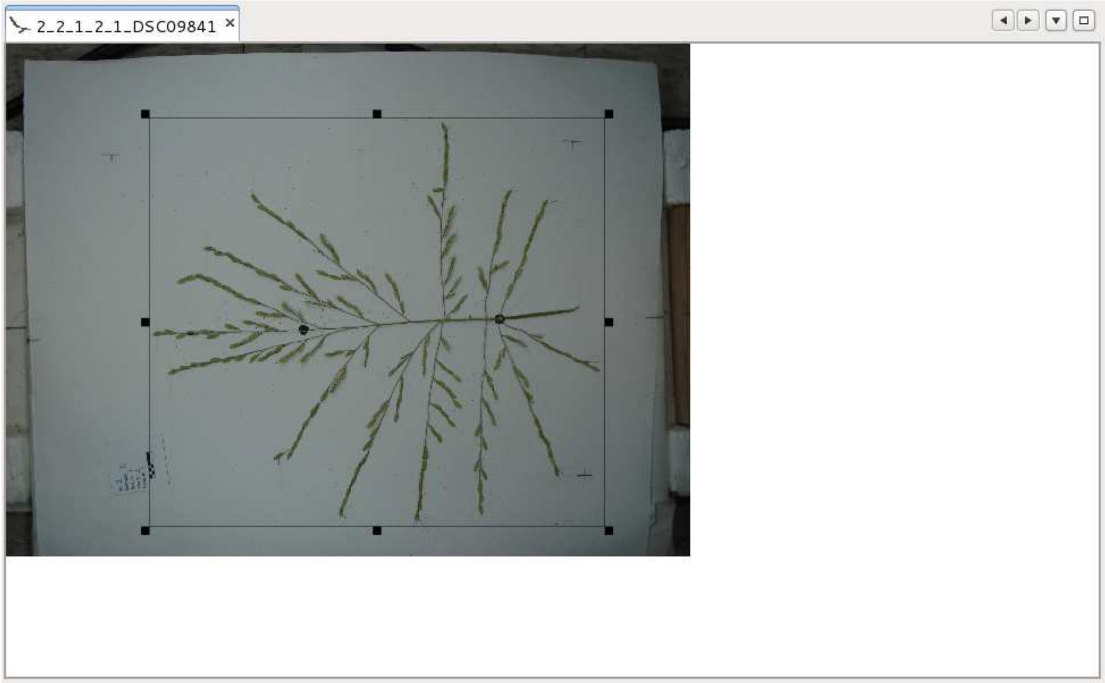

Figure 7: Zoom in/out in the scene in the Image Editor

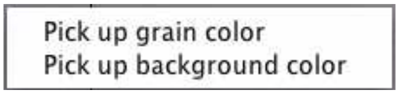

Figure 8: Image editor menu.

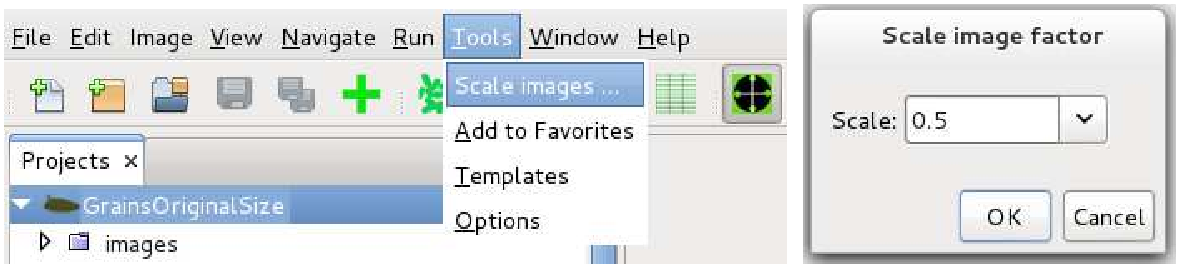

(a) Scale images command (b) Scale images factor dialog

Figure 9: Scale images in a selected project

2.5 Crop an Image

1. Select the cropping area on the image by using the movable and resizable frame, see Figure 7.
2. Click 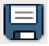.

2.6 Scaling Images

This step allows you to scale your images if they have huge or very small sizes, otherwise it is not necessary. To scale images do the following:

1. Select the project name.
2. Go to the *Tools* menu and select *Scale images*, Figure 9a.
3. In the scaling dialog, choose the scale factor depending on the scale you want to work with and click *OK*, Figure 9b.

Usually, 1024×1024 images are enough for good processing. However, huge-sized images have to be scaled down for better processing, especially for the grain detection task. Therefore, it is strongly recomended to scaledown the images if the grain detection detects only partial parts of the grains.

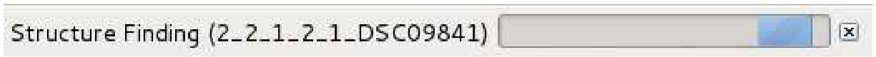

(a) Processing progress

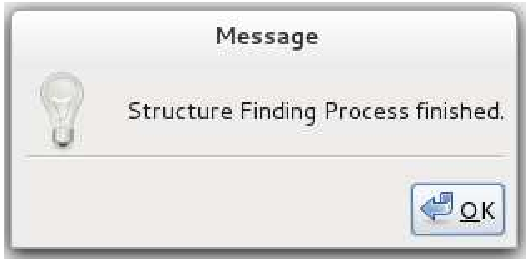

(b) Structure processing notification message

Figure 10: Structure detection notifications

2.7 Working with the Panicle Structure

This section describes how to find the structure both for a single image and for all images in the project.

2.7.1 The Structure of a Single Image

To process a single image in order to find the structure, do the following:-

1. Select the image file and right click to open the pop up menu, Figure 5b.
2. Choose *Find Structure*

During processing the panicle image, a progress bar in the *status panel* of the main window shows the current situation, Figure 10a. When the processing finished the system will notify you by a message as shown in Figure 10b.

When the processing finished, a structure file will be added to the *result/images* folder, Figure 11.

2.7.2 The Structure of Multiple Images

1. Select the project
2. Click on 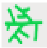 button.

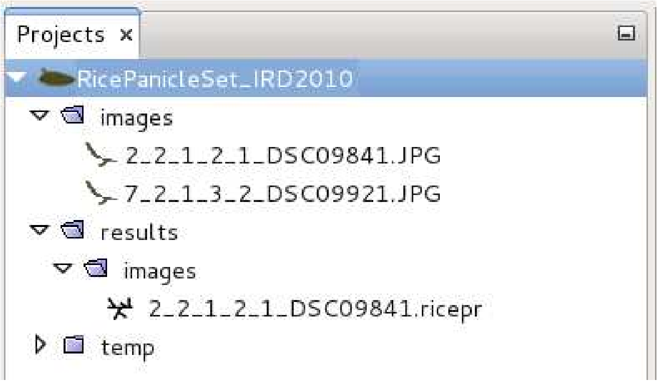

Figure 11: A structure file generated after the image processed

2.7.3 View and Edit the Structure

This is a very important task because it allows the user to manipulate the structure. To view the structure of a given image do the following:-

1. Double click on the structure file in the project, or
2. Right click on the image, a context pop-up menu appears, choose *View/edit results file* menu item.

Use the key-mouse *ctrl+ mouse wheel* combination to zoom in/out the scene. To move the entire scene hold the *mouse wheel* down and move the mouse.

To edit the structure, Table 2.1 shows the required key/mouse combinations:-

Table 2.1: Structure editing key/mouse combinations

Functionality	Command
Add a circle	Double click
Delete a circle	Right click then choose *Delete*, Figure 12
Connect two circles	Hold *ctrl* key down then while pointing on one circle drag the mouse
Remove a link	Click on the link, then move one of its ends to an empty place
To change the type of a circle	Right click then choose the type you want, see Figure 12

Due to the variation from one panicle to another, **the user has to define the start and end generating points (yellow-colored circles)**. This is described in details in Figure 12:-

During post processing the results, the application will show hint if some circles need to be connected, Figure 13.

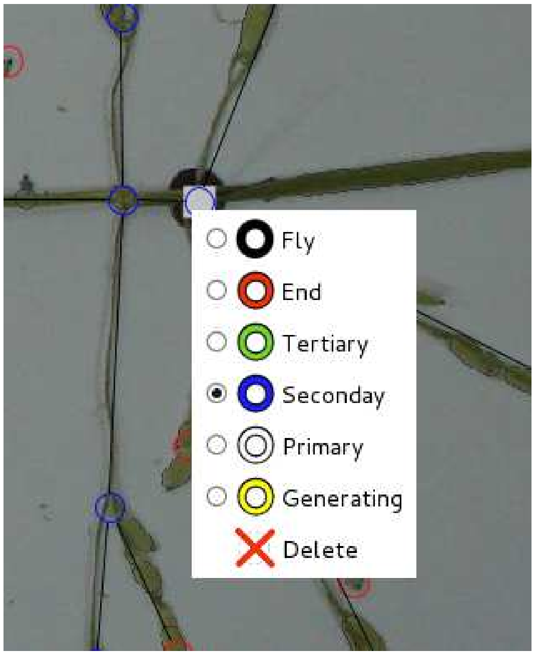

Figure 12: Circles context menu

Once you finished the editing and specifying the start and end generating circles, click 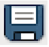 to save the changes you have made.

2.8 Working with the Grains

There are two methods to detect the grains. The first one deals with the clustered grains and is able to detect the grains on the branches. The other method deals with separated grains. This section deals with both cases. In the beginning we explain how to find the grains of a single image, then the post-processing is explained.

2.8.1 Detecting the Grains in a Single Image

To process a single image in order to find the grains, do the following:-

1. Select the image file and right click to open the pop up menu, (Chapter 1, Figure 5b).

2. Choose *Find Grains* if the image has clustered grains, otherwise choose *Find Grains' Traits*

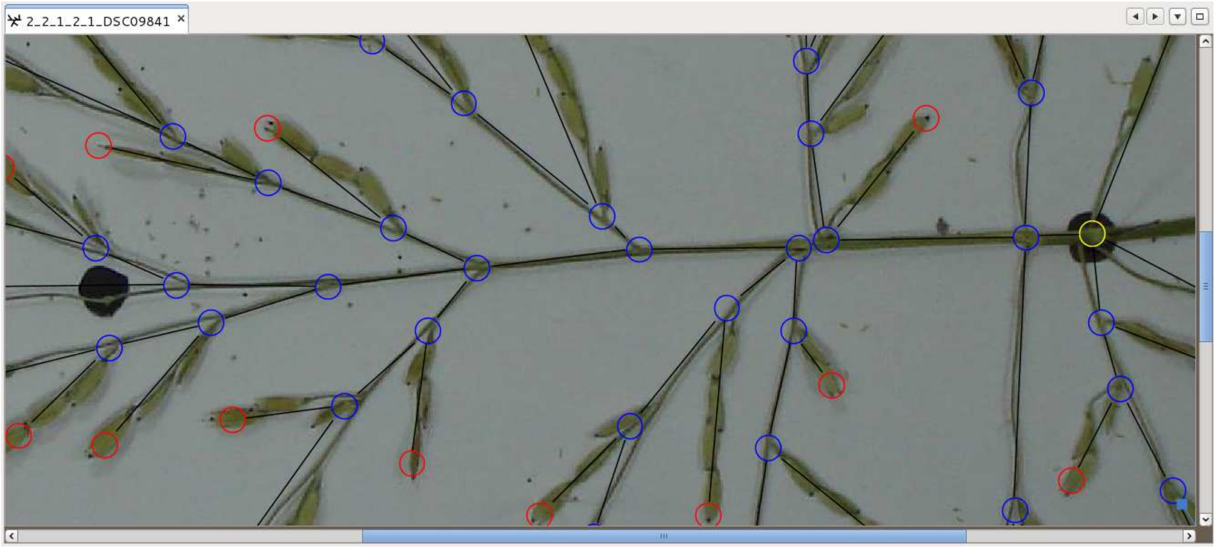

(a) Select the start generating circle, right click, and then choose *Generating* from the menu

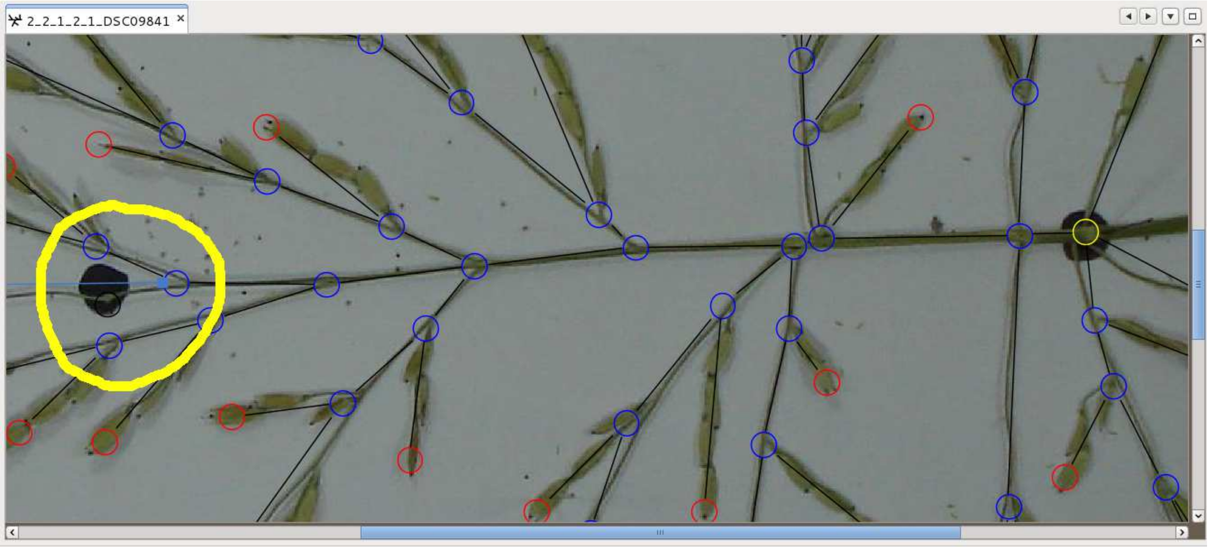

(b) If the end generating point is not detected, double click to create a *flying* circle (*i.e* black)

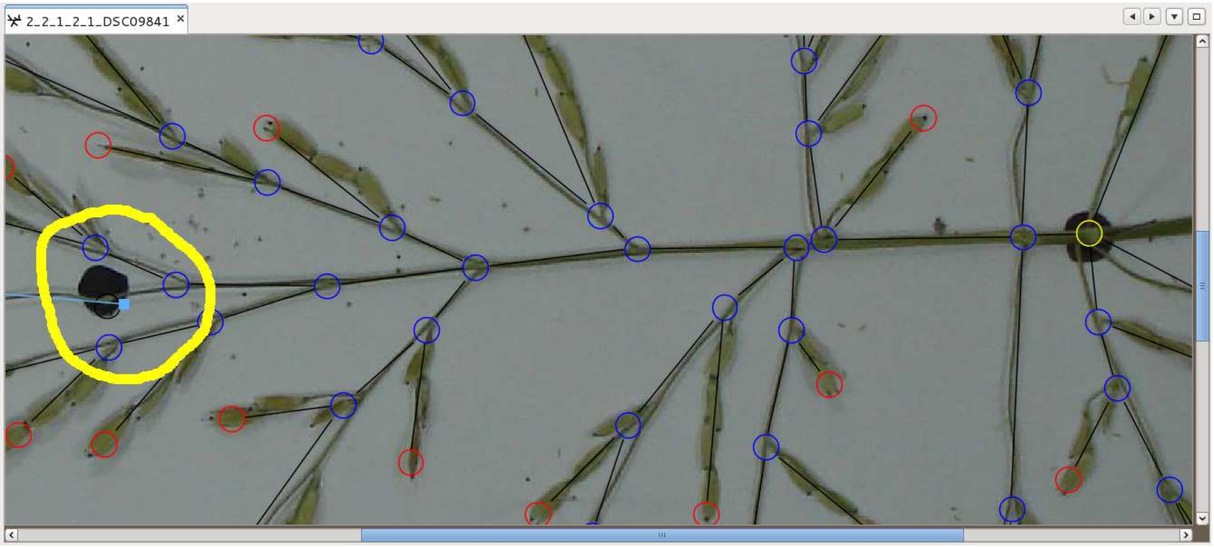

(c) Connect the new circle

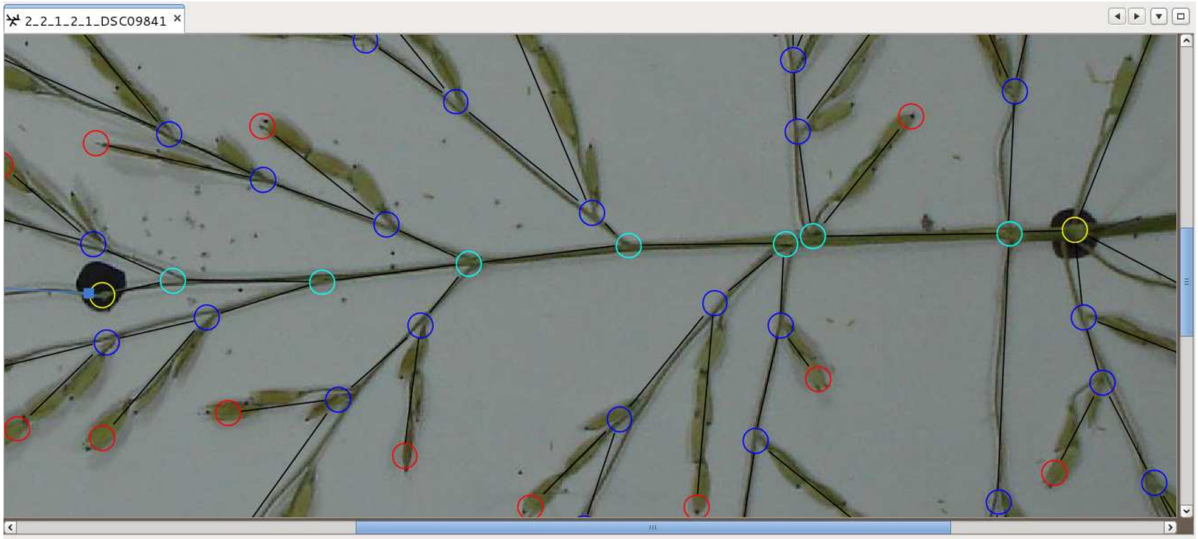

(d) Right click on this new “connected” circle and choose *Generating* from the menu

Figure 12: Defining the start and end generating circles

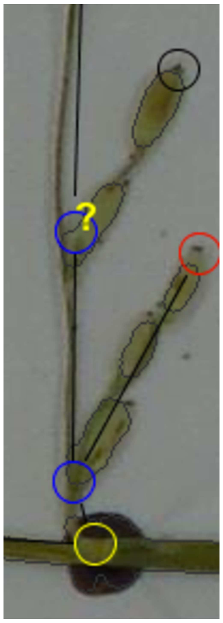

Figure 13: An error hint generated by the application to allow the user locate the error easily.

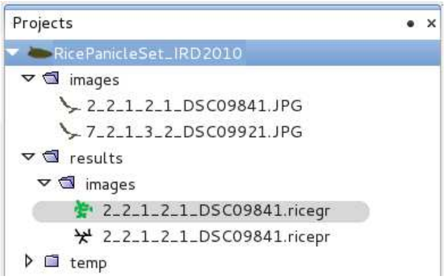

Figure 14: A grains file generated from processing a panicle image.

Once the application finished finding the grains, it will notify the user by a message. As a result, a grain file will be generated as shown in Figure 14.

2.8.2 Detecting the Grains in Multiple Images

1. Select the project.
2. Click 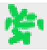 button, if the grains are clustered. Otherwise click 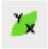 button.

A progress bar will appear in the right bottom of the main window to show the percentage of images being processed. Once it finished, it will show a notification message.

2.8.3 Color-Segmentaion for grains detection

In some cases, the main automatic segmentaton method may have problems finding the acurate bounds of the grains. For this porpose a manual-guided method is available. In this method, the user has to specify the grain and background colors. To define these colors:

- Open the image in the *image editor*.
- *Right-click* to open a pop up menu as shown in Figure 8.
- select the colors.

To run in color-segmentation mode, toggle ON the *color segemention*, 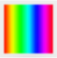

2.8.4 View/Edit the Grains Files

To view a grain file do one of the following:

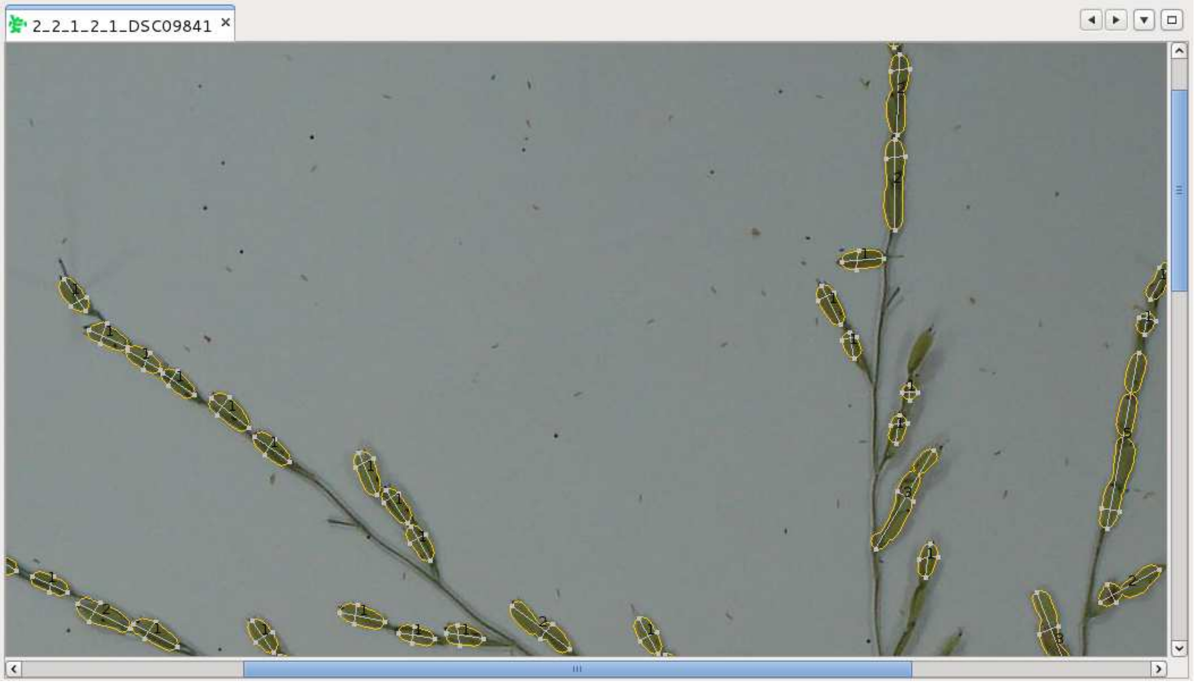

Figure 15: The anchors of the grains.

1. Double click on the grain file in the project, or

2. Right click on the image, a context pop-up menu appears, choose *View/edit grains file*.

To view the the grains major and minor axes, Figure 15, toggle the *View Anchors* 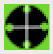 button *ON*.

Editing the grains file includes adding, removing, and correcting the number of grains. To add a grain, right click in the place you want to add a grain and then choose *add grain*. To remove a grain or a grains' cluster, right click on the grain and then choose *remove grains*. For modifying the number of grains, double click on the grain then write the correct number of grains, Figure 16.

Once you finish post-processing the grains, click the save button 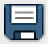 to save the changes you have made.

2.9 Collecting the Reports

As mentioned before, P-TRAP generates three reports in Comma Separated Values (CSV) format. Once the processing and post-processing tasks are performed, the final reports can be

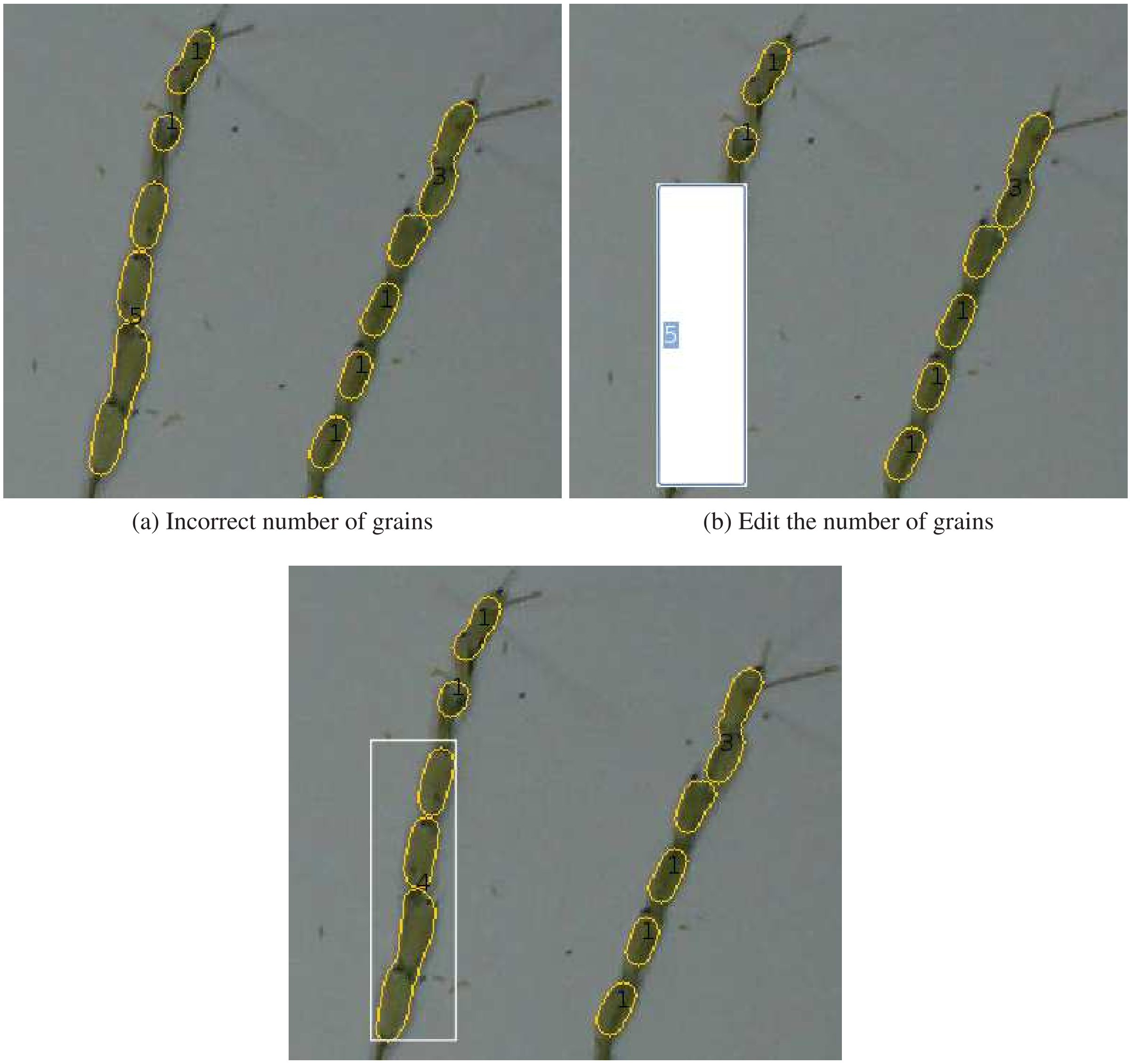

(a) Incorrect number of grains

(b) Edit the number of grains

(c) Corrected number of grains

Figure 16: Grains number correction.

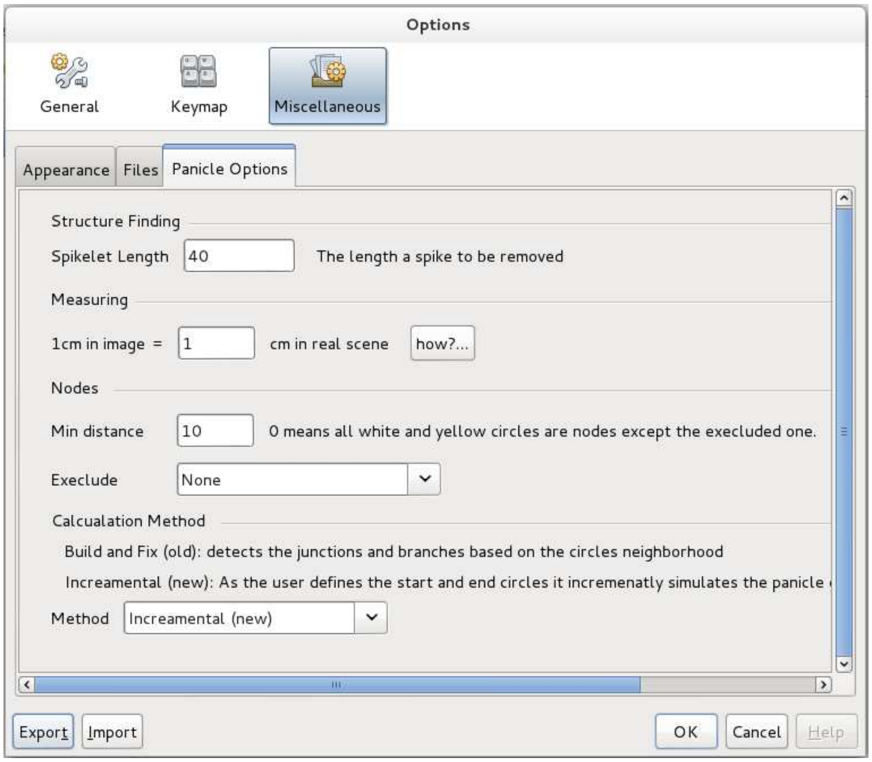

Figure 17: The panicle options dialog

generated by:

1. Select the project
2. Click 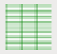 button.

When the application finished collecting the results of all images, it will notify the user by a message. The reports files are shown in Figure 10.

2.10 Options

P-TRAP offers a set of options for the graphical interface and the core process as well. This sections describes the processing options. The panicle options can be found in: *Tools* ⟶ *Options* menu, then choose *Panicle Options*, Figure 17. These options are explained in Table 2.2.

Table 2.2: P-TRAP panicle options

Option	Description
**Structure Finding**	
Spikelet Length	Controls the minimum length of the branch, any branch that has length less than this value will be mark as a noise and removed.
1cm	This option defines the ratio between the cm in the image and in th real panicle.
**Nodes**	
Min distance	Controls the minimum distance between two nodes in the main axis in the panicle. If the distance between two nodes is less than this value they are counted as a single node.
Exclude	Which nodes has to be excluded from counting?
**Calculation Method**	
Method	Which method that applications has to use in order to detect the panicle structure? Usually the incremental method works better.

2.10.1 Defining the Scale

The scale label is widely used in many biological work. P-TRAP allows the user to define the measuring scale in the image. The following steps explain how to define the measuring scale.

1. Open the image that contains the scale by double-clicking on the image.

2. Drag from the one end of the scale, Figure 18a, and hold until you reach the other end then right click, Figure 18b.

3. The scale dialog appear, Figure 18c define how many unite in the scale and click *OK*.

The application will then use the ratio between the real panicle size and the panicle size in the image to make accurate calculations.

2.10.2 Image's Background

In some cases, the images backgrounds are darker than the objects. Therefore, P-TRAP offers an option such that the user defines the type of the image's background. The default option is white, you can change it to dark by toggling the *Dark Background* button *ON*.

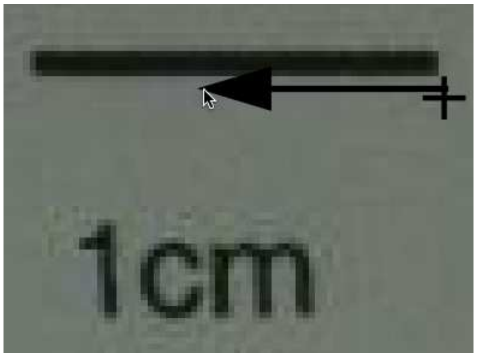

(a) Drage on one end of the scale

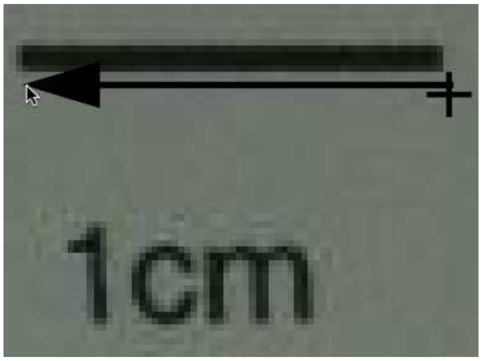

(b) Righ-click once the other end is reached

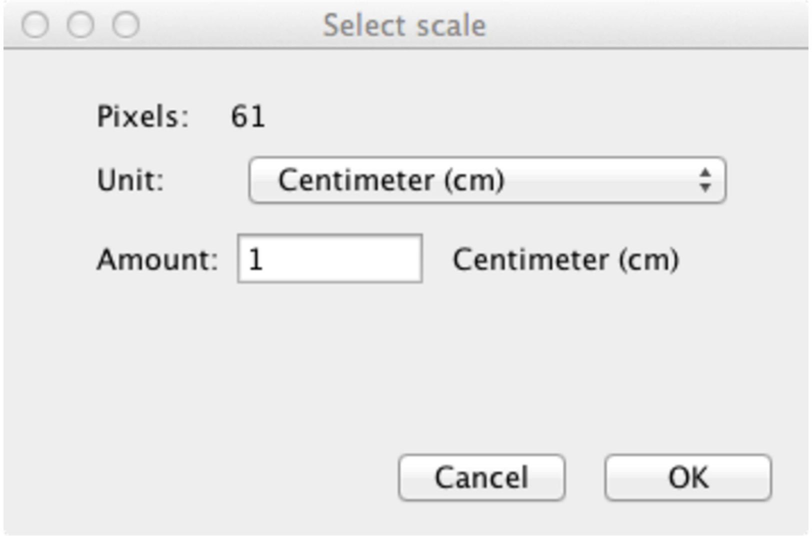

(c) Define how many unites in the scale

Figure 18: Defining the scale in image.

3 Troubleshooting

- *Not very good detection.*

  1- Try to scale down the images if they are very large or crop the unwanted background or artifacts at the borders.

  2- If the background is dark try to use the **dark background** 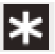 option.

- *Unable to save the detected structure.* Try to remove all flying circles (black colors) you can use the right-click menu to remove them all. Also make sure you have determined the start and end generating points.

- *The report is empty.* Make sure you have defined the start and end generating points in

the structure.

Appendix A

XML Files

1 Structure XML File

The structure XML file is compactly describing the structure of the panicle as a mathematical graph composed of vertices and edges. What is needed to use this file is any XML parser plugged in your application to convert this file into your graph data structure, by tackling only the <graph> entity and its components. The entire structure of the file is described as the following:

- **<result signature="STRUCTUE" imagepath="18_2_2_1_2_DSC09873.JPG">**

  The is the main tag, which means this is a structure result file identified by the signature, "STRUCTURE".

- <**graph**> Describes a graph of vertices and edges, this graph represents the panicle structure.

- <**vertices**> contains a set of vertices (these are converted to cycles as you can see them in the application). They describ the junctions and the ends of the panicle structure.

- **<vertex id="java.awt.Point[x=994,y=1282" x="994" y="1282" type="End" fixed="false"/>** A single vertex. Its attributes are:-

  - **id**: an identifier of the vertex,

  - **x and y**: the xy-coordinates of the vertices,
  - **type**: the type of the vertex,
  - **isfixed**: true if the vertex has been edited by the user.

- <**edges**> The edges that connects the graph vertices
- **<edge vertex1="java.awt.Point[x=1047,y=1053]" vertex2="java.awt.Point[x=1149,y=1015]"/>** an edge that connects two vertices given their identifiers "vertex1" and "vertex2".
