# Supplementary figures and images for: P-TRAP: a Panicle Trait Phenotyping tool
Source: BMC Plant Biol. 2013 Aug 29;13:122. doi: 10.1186/1471-2229-13-122 (PMC3848748; doi:10.1186/1471-2229-13-122)

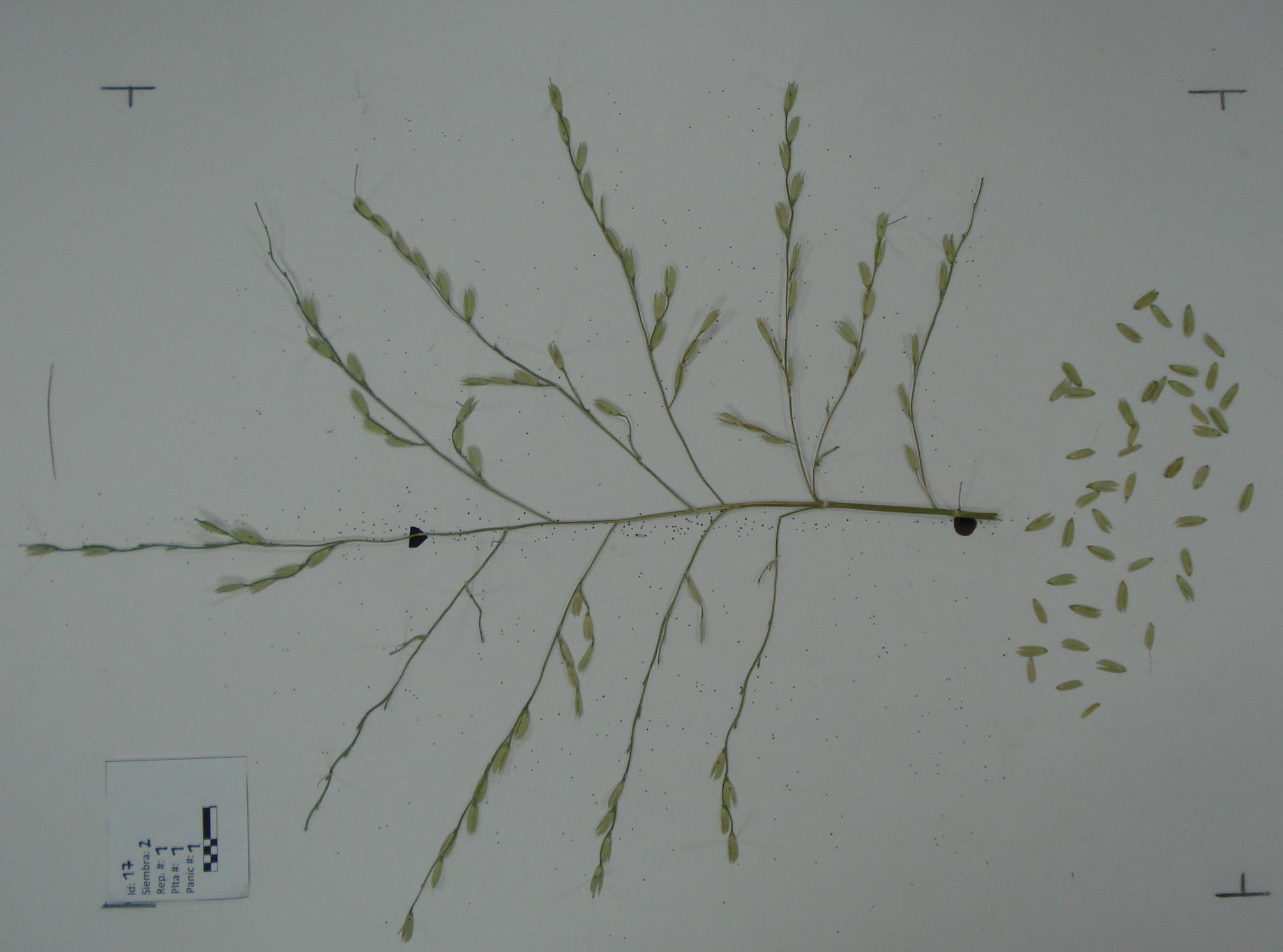

Supplement: Additional file 3 — 26 images of spread out panicles. A set of images of spread out panicles used to test the application for the detection of the structure, counting the grains or spikelets and for the detection of grain traits. [file 1471-2229-13-122-S3.zip › Additional file 3/17_2_1_1_1_DSC09942.JPG]

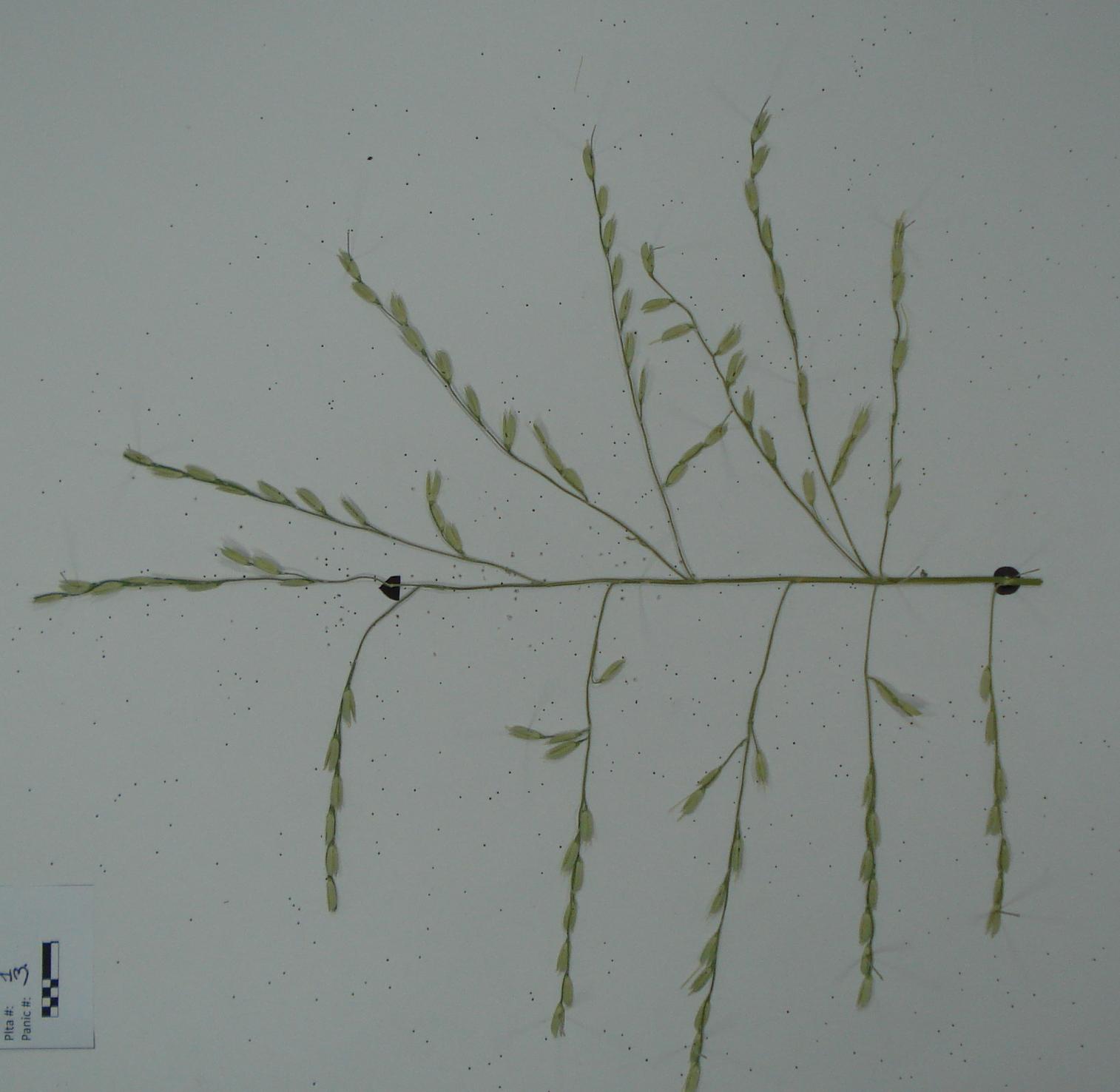

Supplement: Additional file 3 — 26 images of spread out panicles. A set of images of spread out panicles used to test the application for the detection of the structure, counting the grains or spikelets and for the detection of grain traits. [file 1471-2229-13-122-S3.zip › Additional file 3/17_2_1_1_3_DSC09940.JPG]

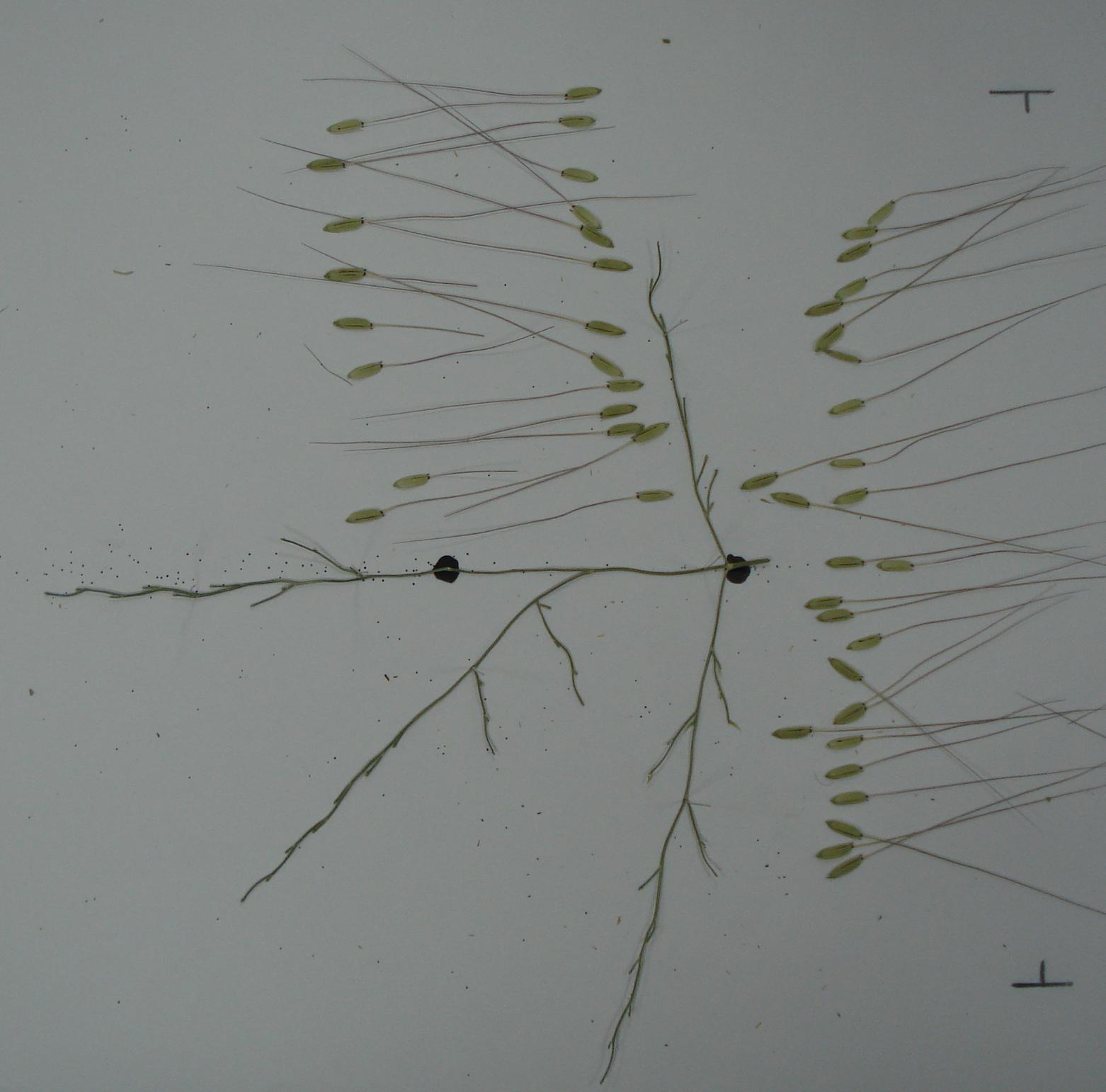

Supplement: Additional file 3 — 26 images of spread out panicles. A set of images of spread out panicles used to test the application for the detection of the structure, counting the grains or spikelets and for the detection of grain traits. [file 1471-2229-13-122-S3.zip › Additional file 3/18_2_1_2_1_DSC09864.JPG]

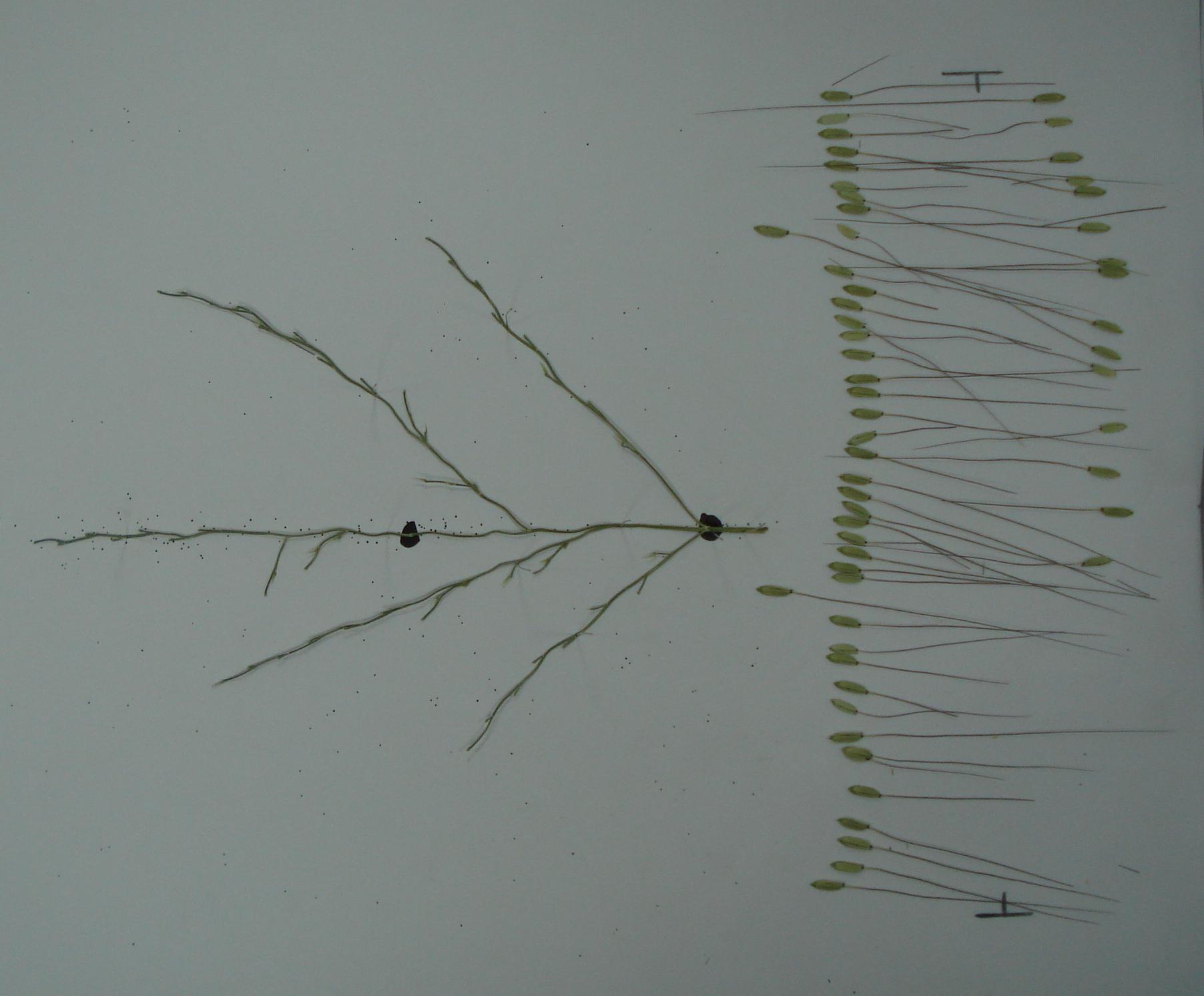

Supplement: Additional file 3 — 26 images of spread out panicles. A set of images of spread out panicles used to test the application for the detection of the structure, counting the grains or spikelets and for the detection of grain traits. [file 1471-2229-13-122-S3.zip › Additional file 3/18_2_2_3_2_DSC09880.JPG]

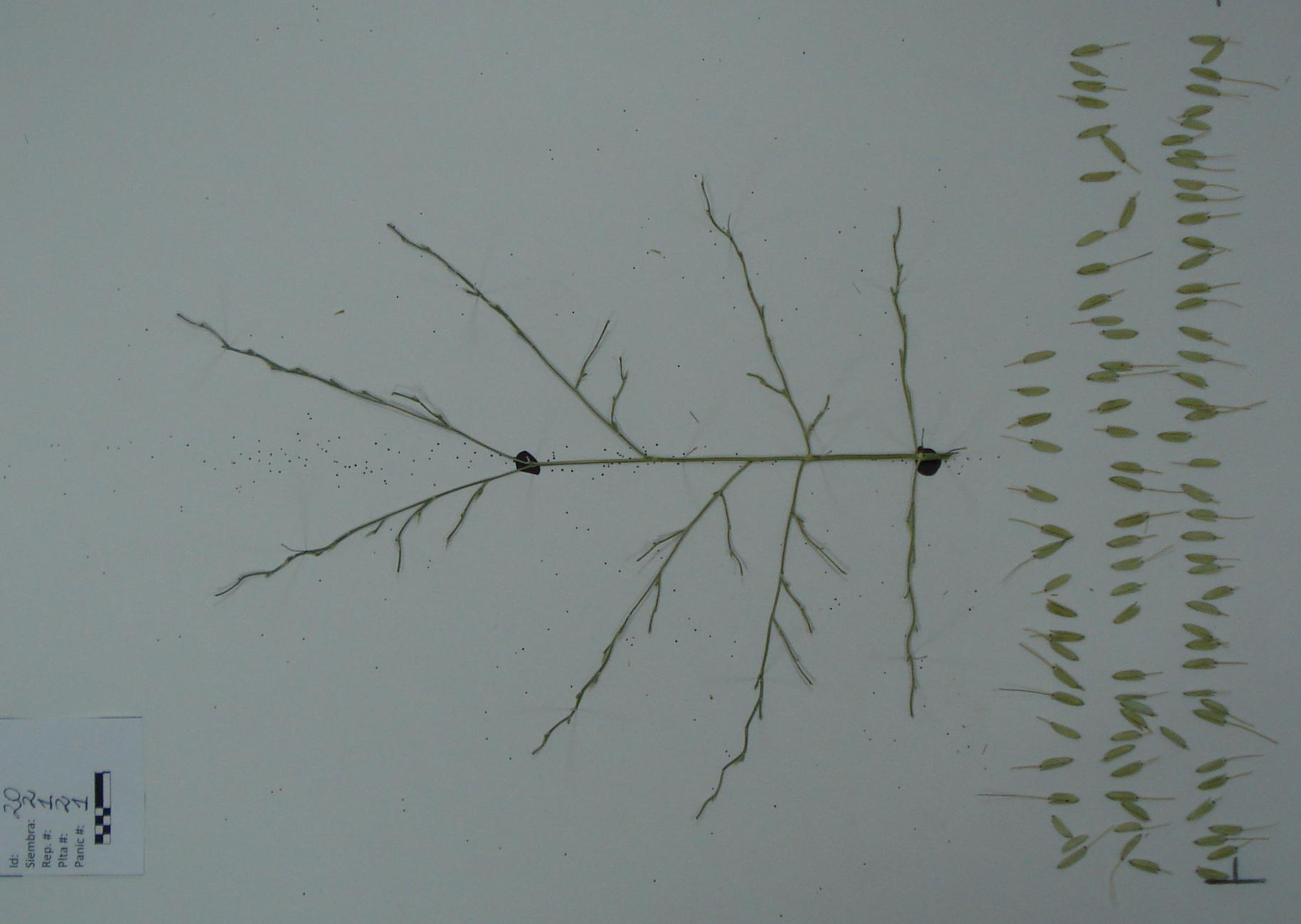

Supplement: Additional file 3 — 26 images of spread out panicles. A set of images of spread out panicles used to test the application for the detection of the structure, counting the grains or spikelets and for the detection of grain traits. [file 1471-2229-13-122-S3.zip › Additional file 3/20_2_1_2_1_DSC09887.JPG]

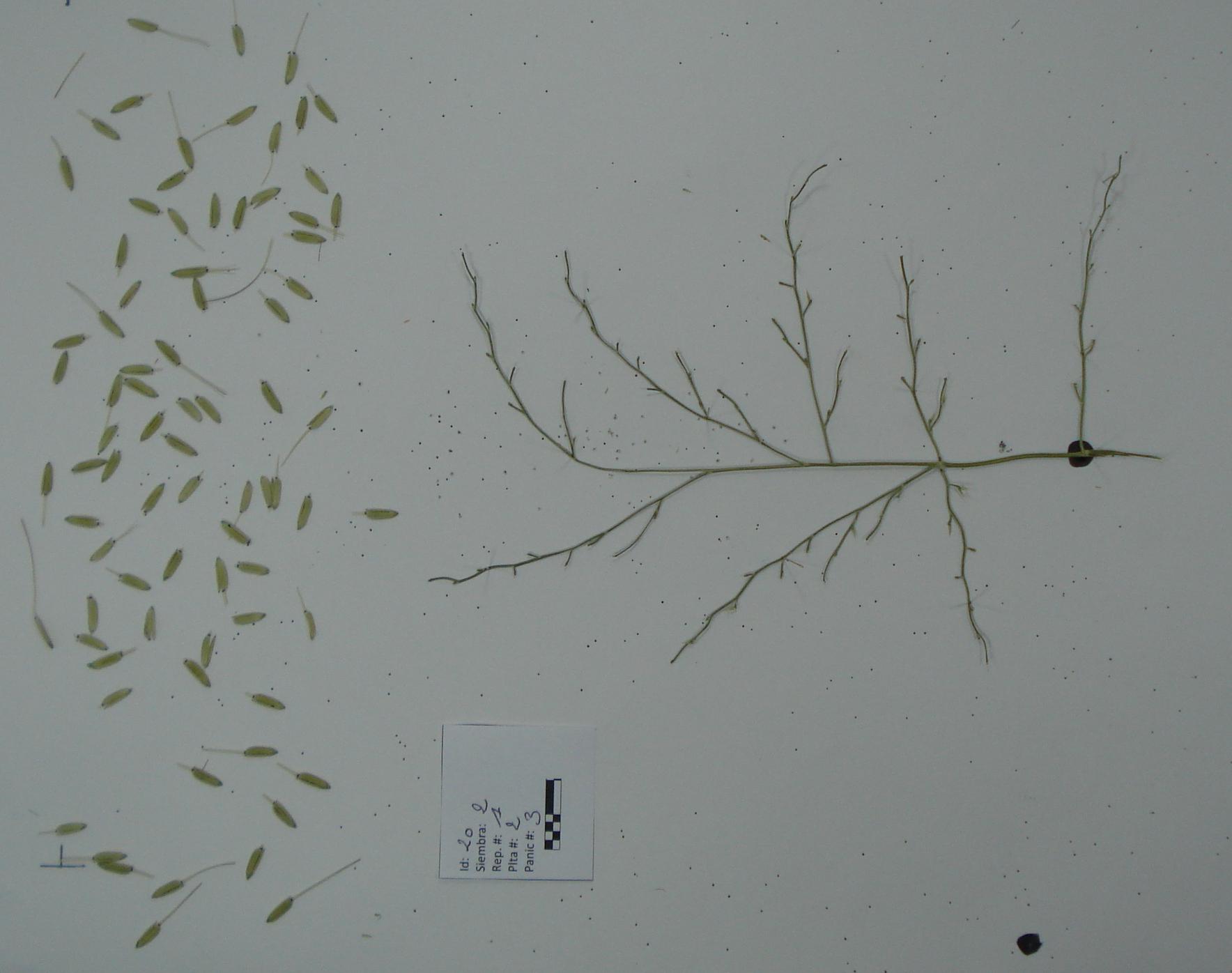

Supplement: Additional file 3 — 26 images of spread out panicles. A set of images of spread out panicles used to test the application for the detection of the structure, counting the grains or spikelets and for the detection of grain traits. [file 1471-2229-13-122-S3.zip › Additional file 3/20_2_1_2_3_DSC09888.JPG]

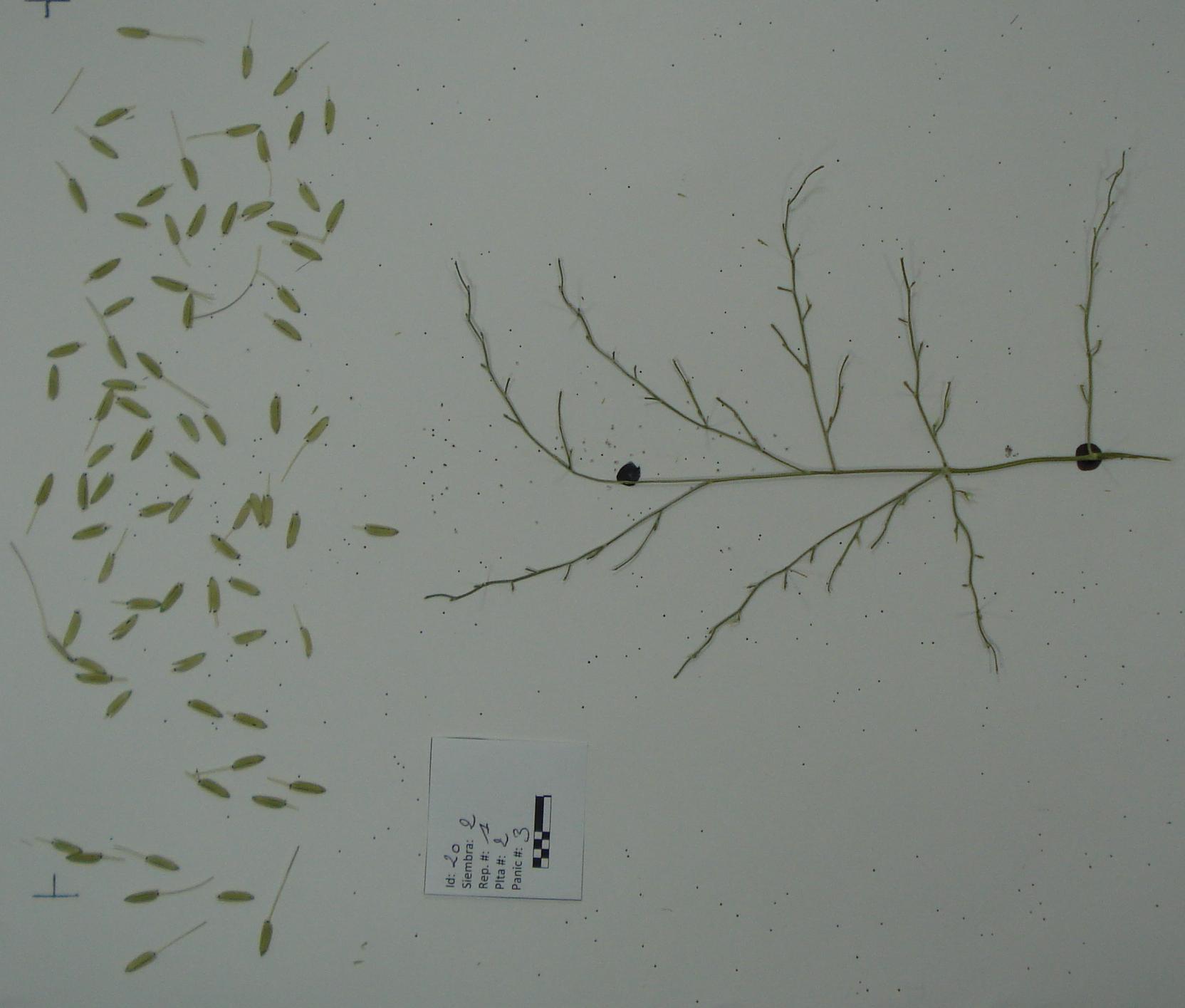

Supplement: Additional file 3 — 26 images of spread out panicles. A set of images of spread out panicles used to test the application for the detection of the structure, counting the grains or spikelets and for the detection of grain traits. [file 1471-2229-13-122-S3.zip › Additional file 3/20_2_1_2_3_DSC09889.JPG]

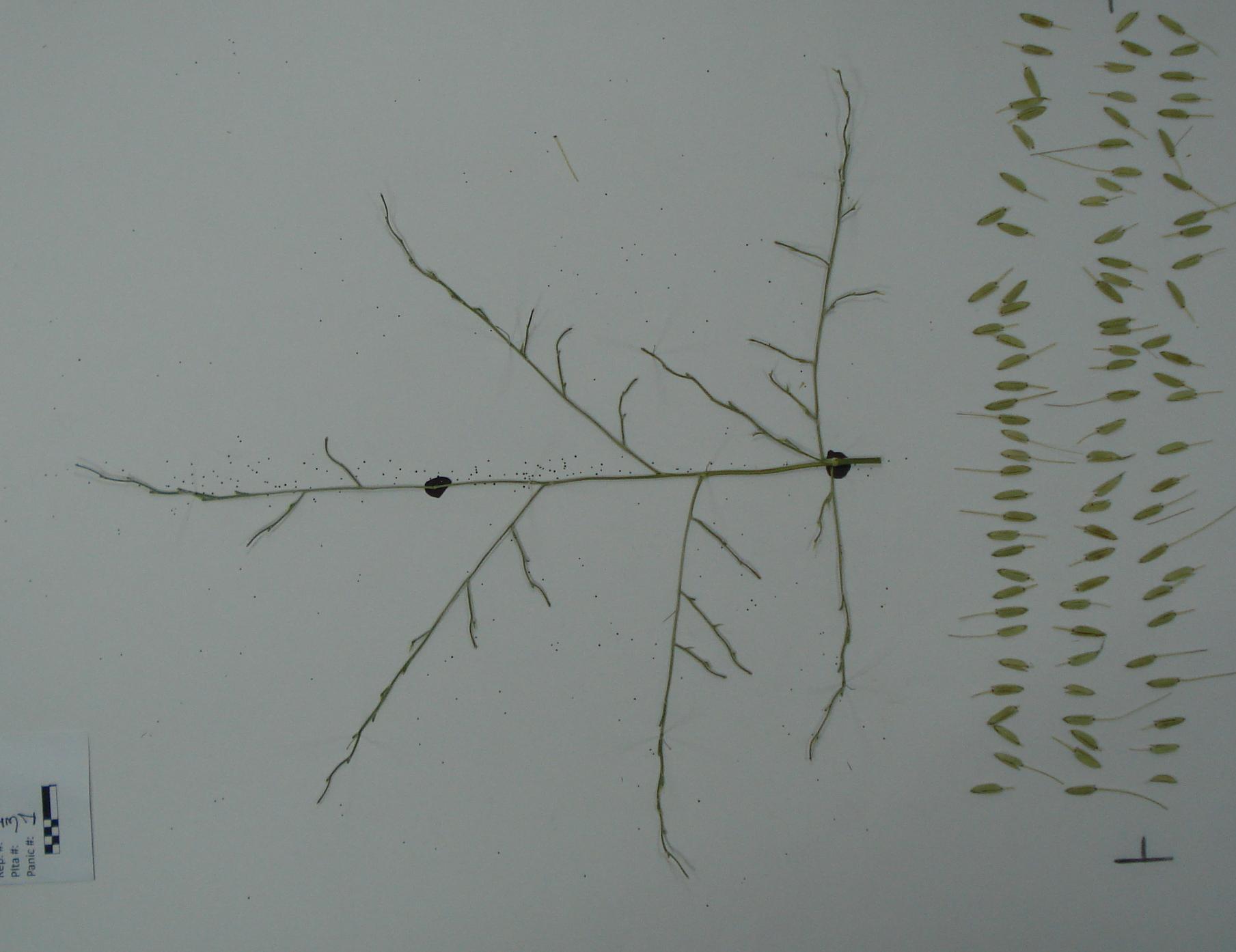

Supplement: Additional file 3 — 26 images of spread out panicles. A set of images of spread out panicles used to test the application for the detection of the structure, counting the grains or spikelets and for the detection of grain traits. [file 1471-2229-13-122-S3.zip › Additional file 3/20_2_1_3_1_DSC09891.JPG]

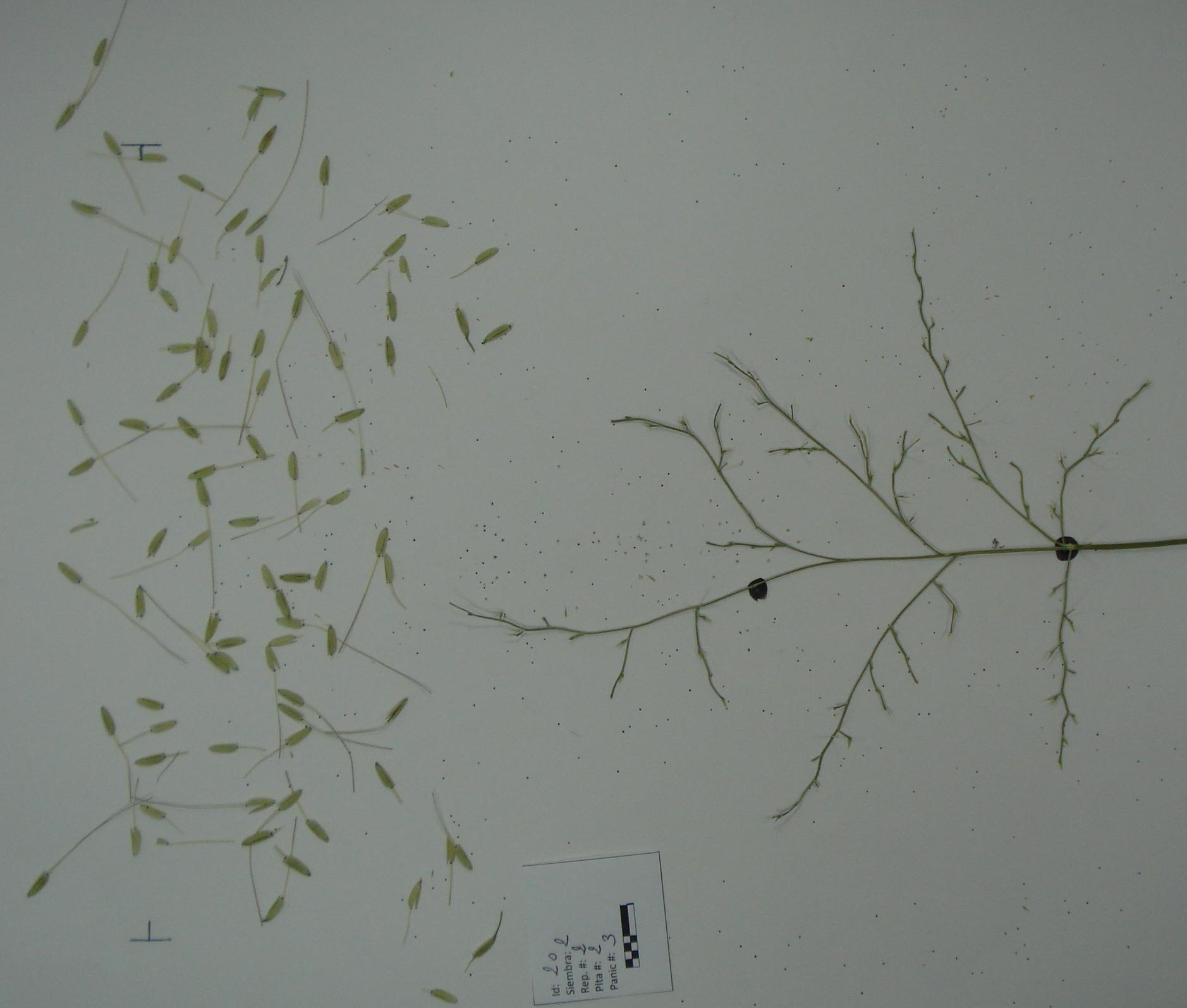

Supplement: Additional file 3 — 26 images of spread out panicles. A set of images of spread out panicles used to test the application for the detection of the structure, counting the grains or spikelets and for the detection of grain traits. [file 1471-2229-13-122-S3.zip › Additional file 3/20_2_2_2_3_DSC09898.JPG]

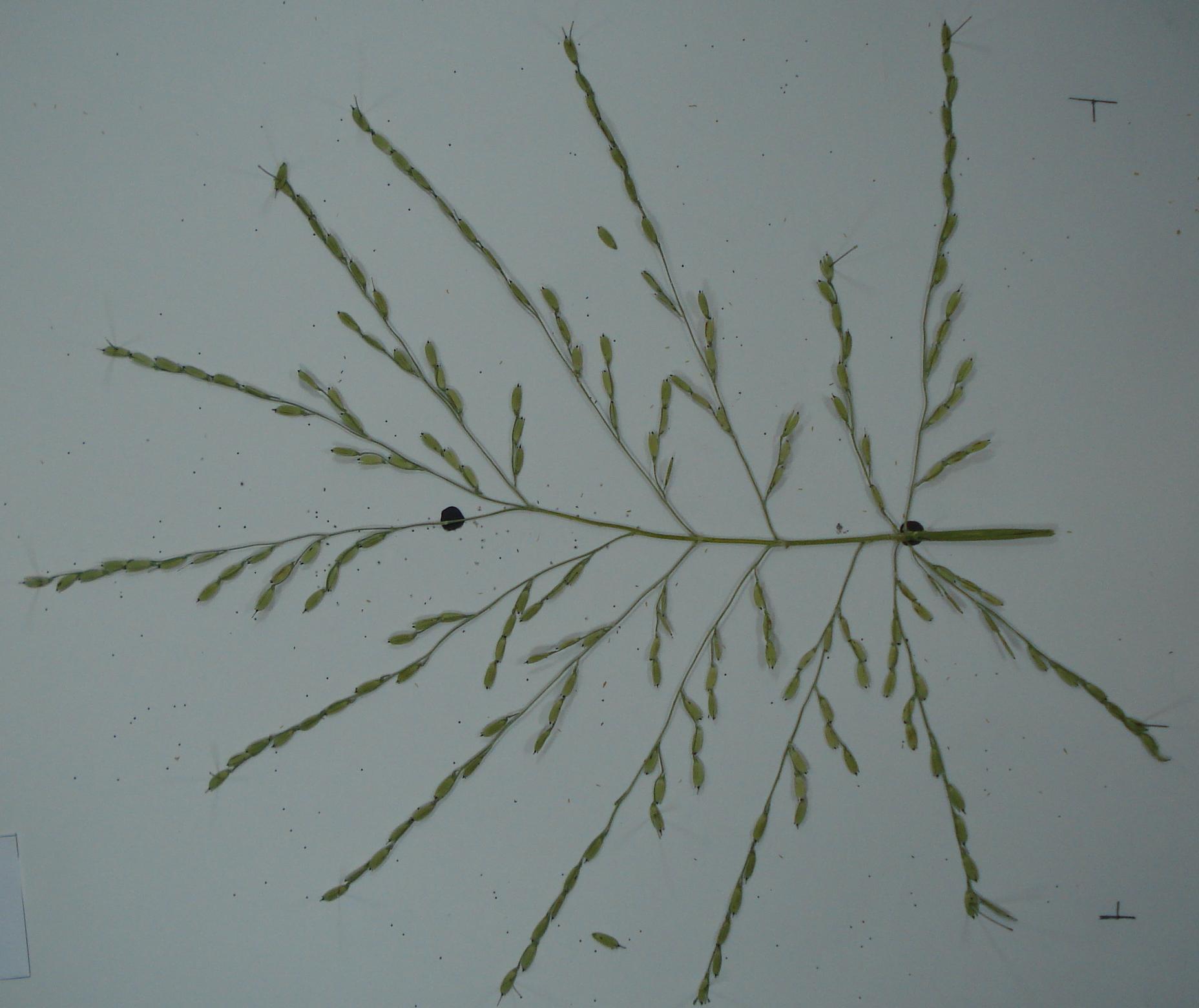

Supplement: Additional file 3 — 26 images of spread out panicles. A set of images of spread out panicles used to test the application for the detection of the structure, counting the grains or spikelets and for the detection of grain traits. [file 1471-2229-13-122-S3.zip › Additional file 3/2_2_2_1_1_DSC09848_.JPG]

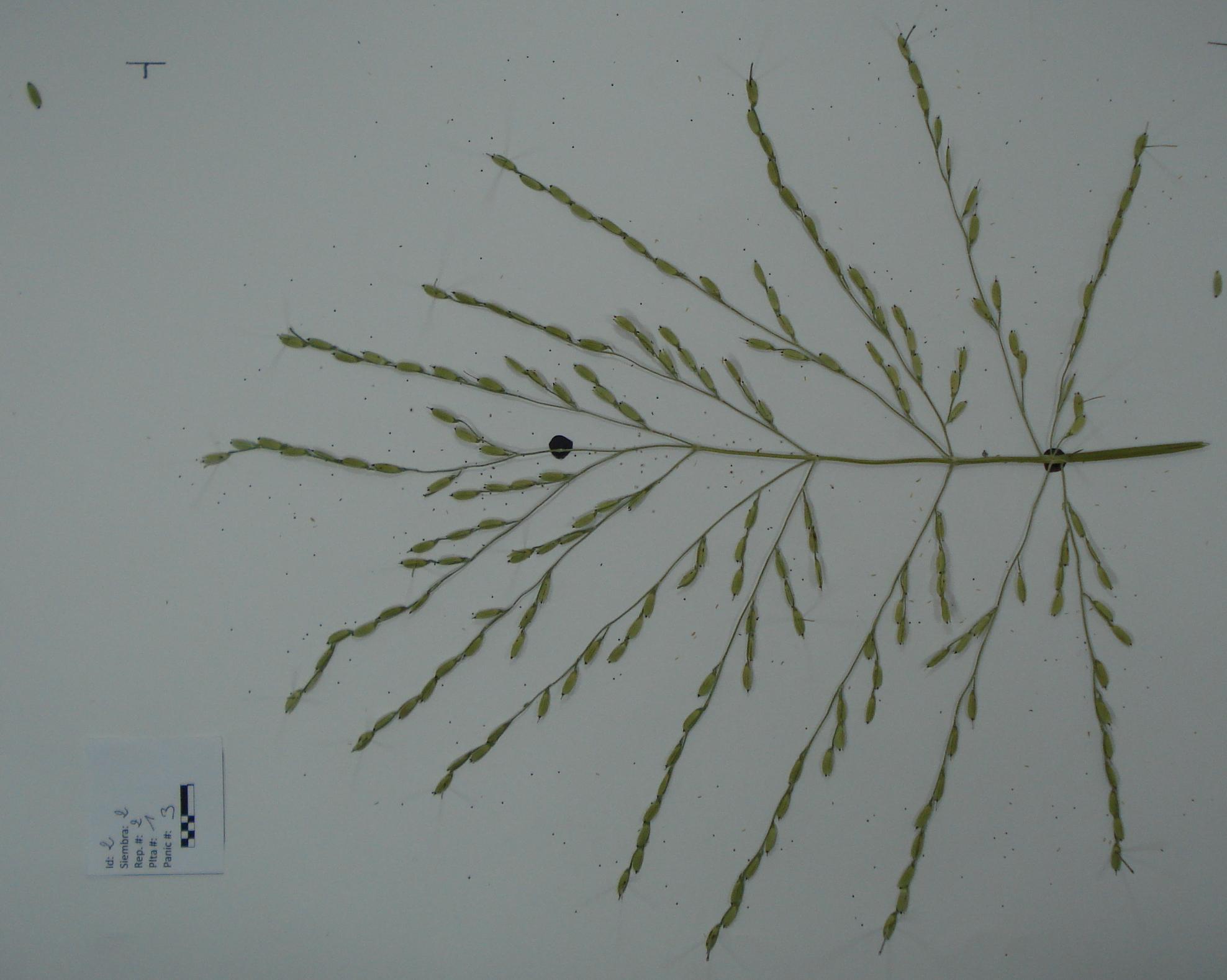

Supplement: Additional file 3 — 26 images of spread out panicles. A set of images of spread out panicles used to test the application for the detection of the structure, counting the grains or spikelets and for the detection of grain traits. [file 1471-2229-13-122-S3.zip › Additional file 3/2_2_2_1_3_DSC09851_.JPG]

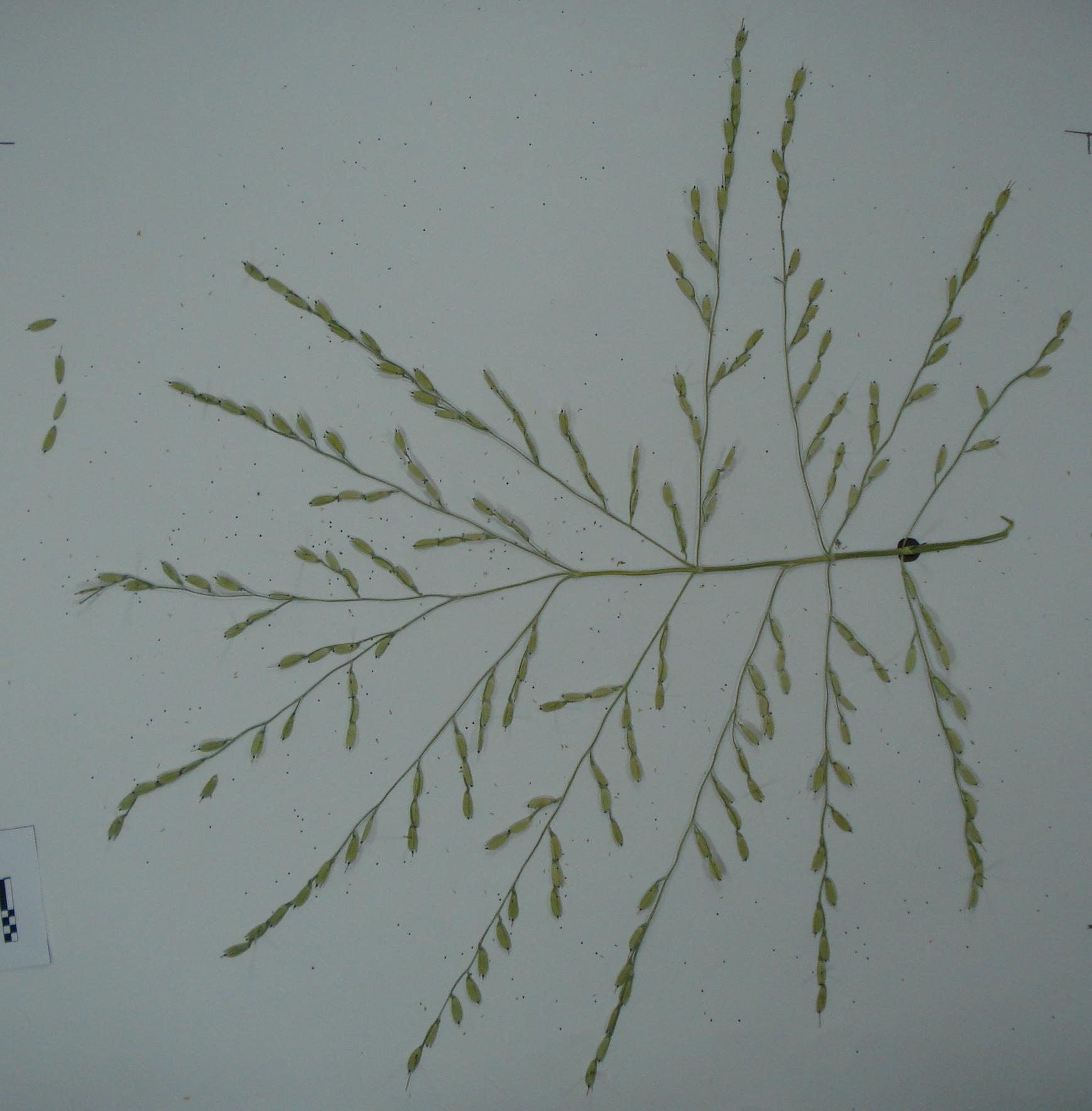

Supplement: Additional file 3 — 26 images of spread out panicles. A set of images of spread out panicles used to test the application for the detection of the structure, counting the grains or spikelets and for the detection of grain traits. [file 1471-2229-13-122-S3.zip › Additional file 3/2_2_2_2_3_DSC09854_.JPG]

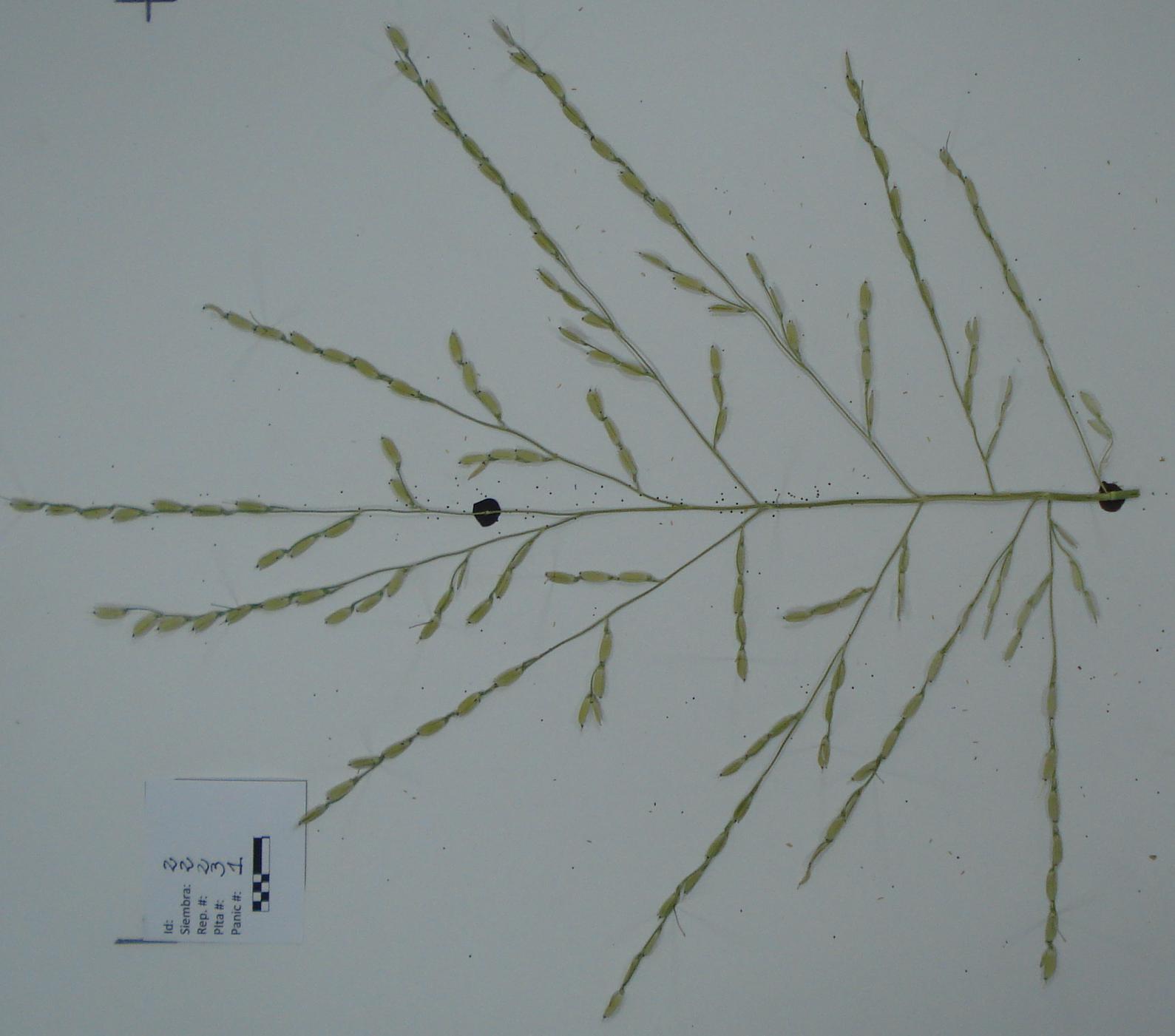

Supplement: Additional file 3 — 26 images of spread out panicles. A set of images of spread out panicles used to test the application for the detection of the structure, counting the grains or spikelets and for the detection of grain traits. [file 1471-2229-13-122-S3.zip › Additional file 3/2_2_2_3_1_DSC09856_.JPG]

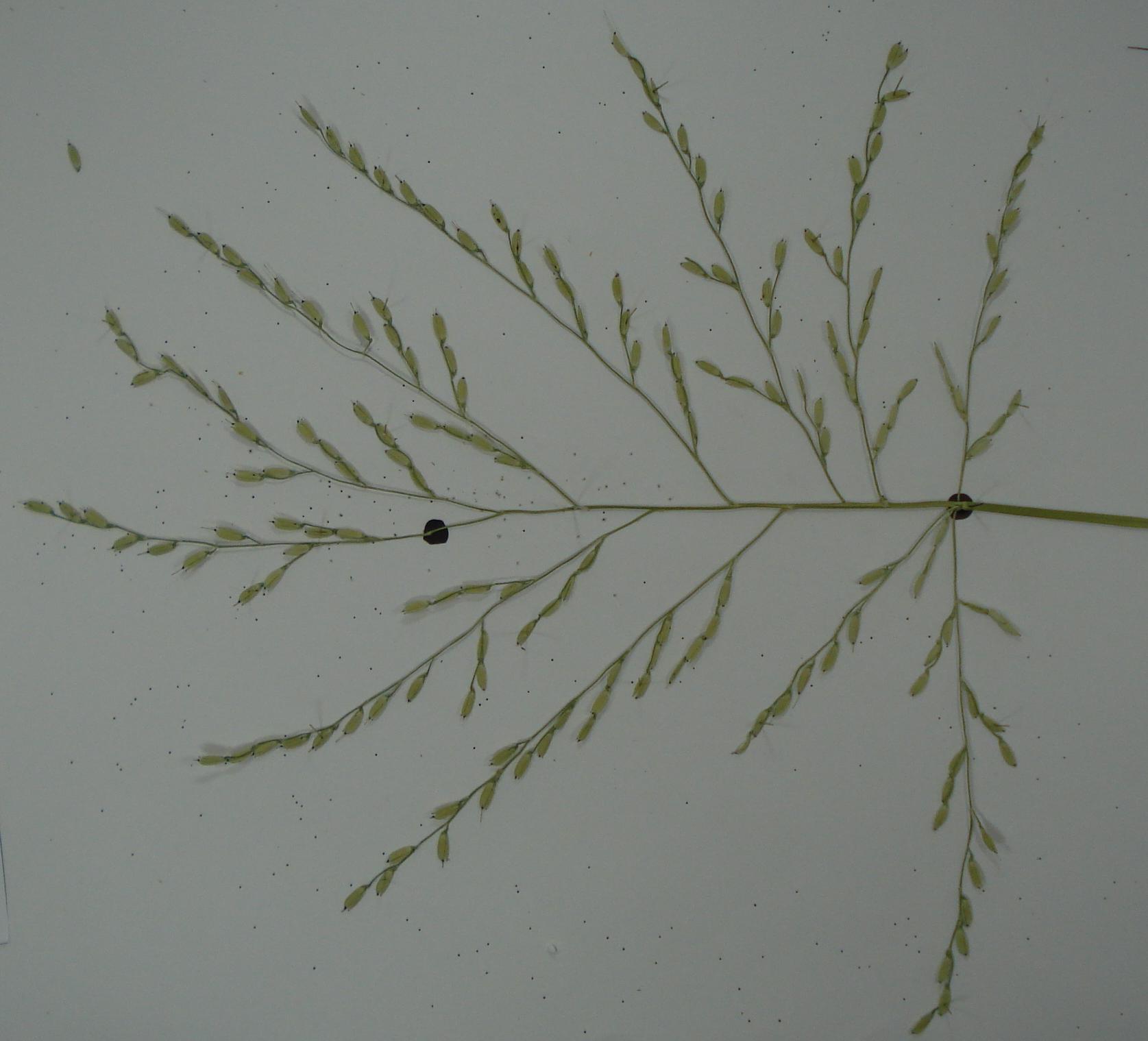

Supplement: Additional file 3 — 26 images of spread out panicles. A set of images of spread out panicles used to test the application for the detection of the structure, counting the grains or spikelets and for the detection of grain traits. [file 1471-2229-13-122-S3.zip › Additional file 3/2_2_2_3_2_DSC09857_.JPG]

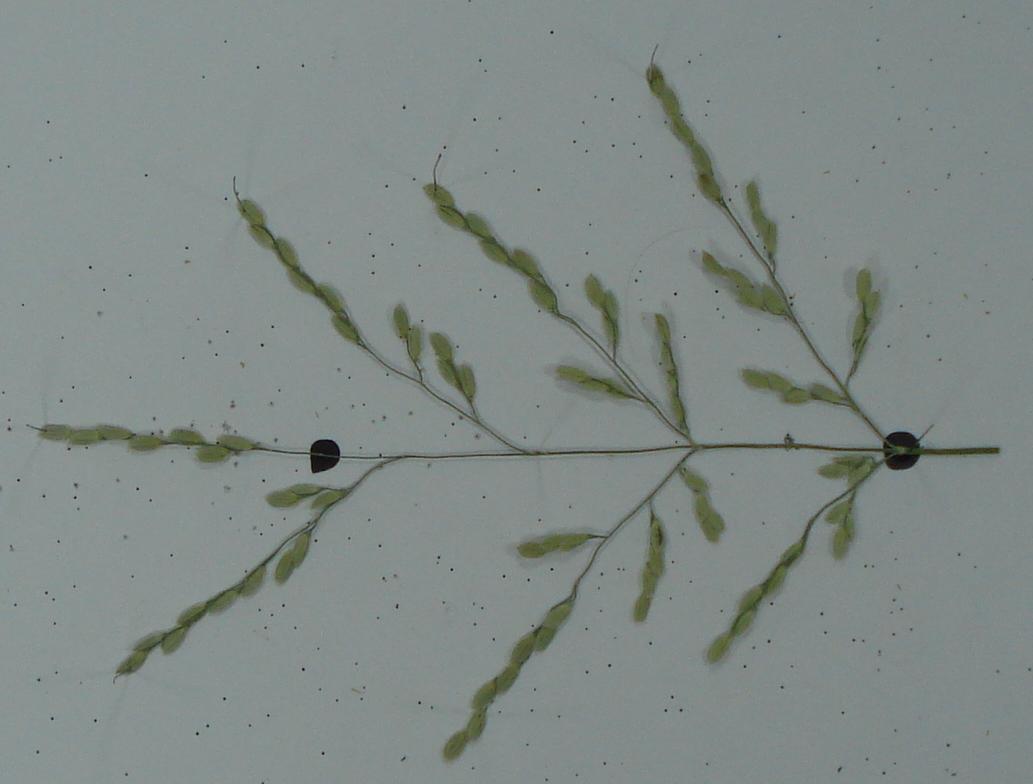

Supplement: Additional file 3 — 26 images of spread out panicles. A set of images of spread out panicles used to test the application for the detection of the structure, counting the grains or spikelets and for the detection of grain traits. [file 1471-2229-13-122-S3.zip › Additional file 3/38_2_2_1_2_DSC09928.JPG]

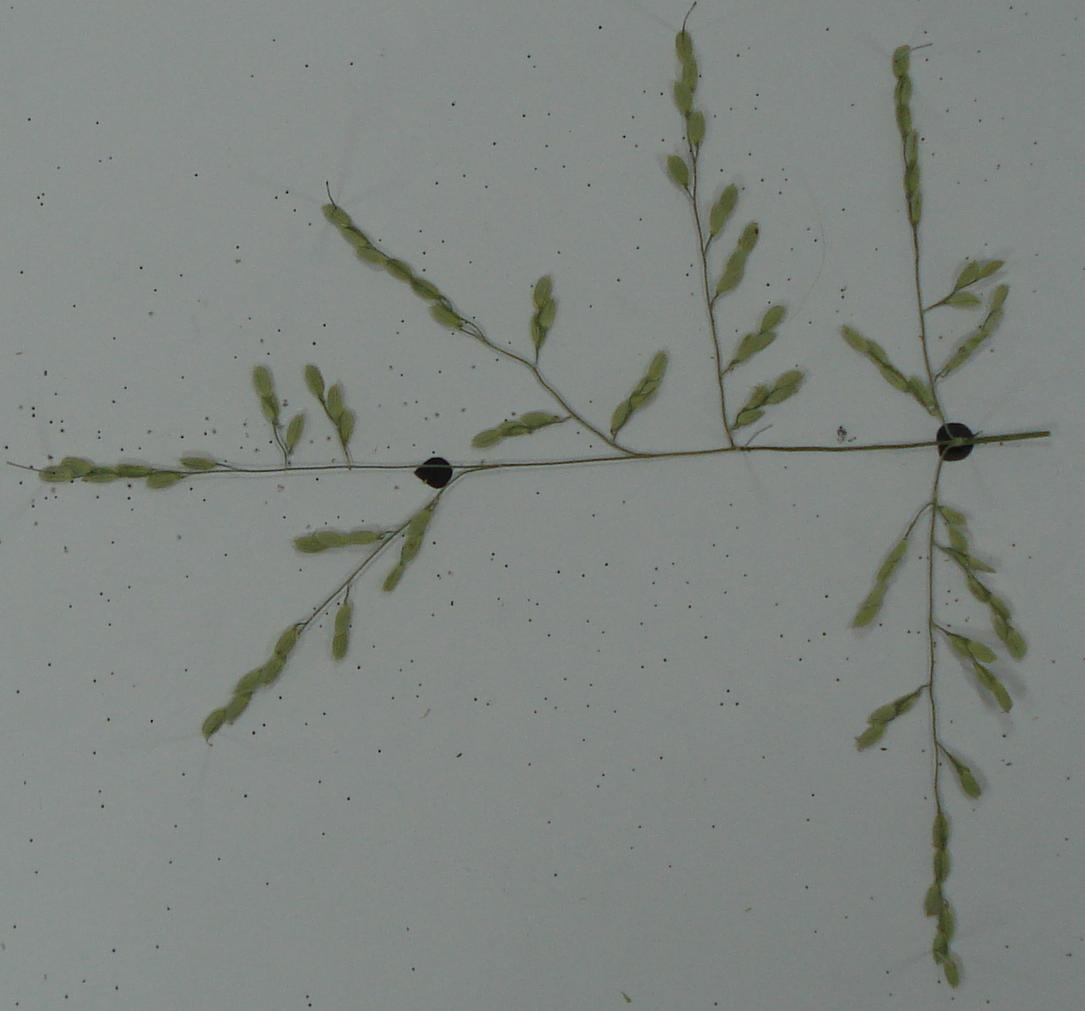

Supplement: Additional file 3 — 26 images of spread out panicles. A set of images of spread out panicles used to test the application for the detection of the structure, counting the grains or spikelets and for the detection of grain traits. [file 1471-2229-13-122-S3.zip › Additional file 3/38_2_2_2_1_DSC09930.JPG]

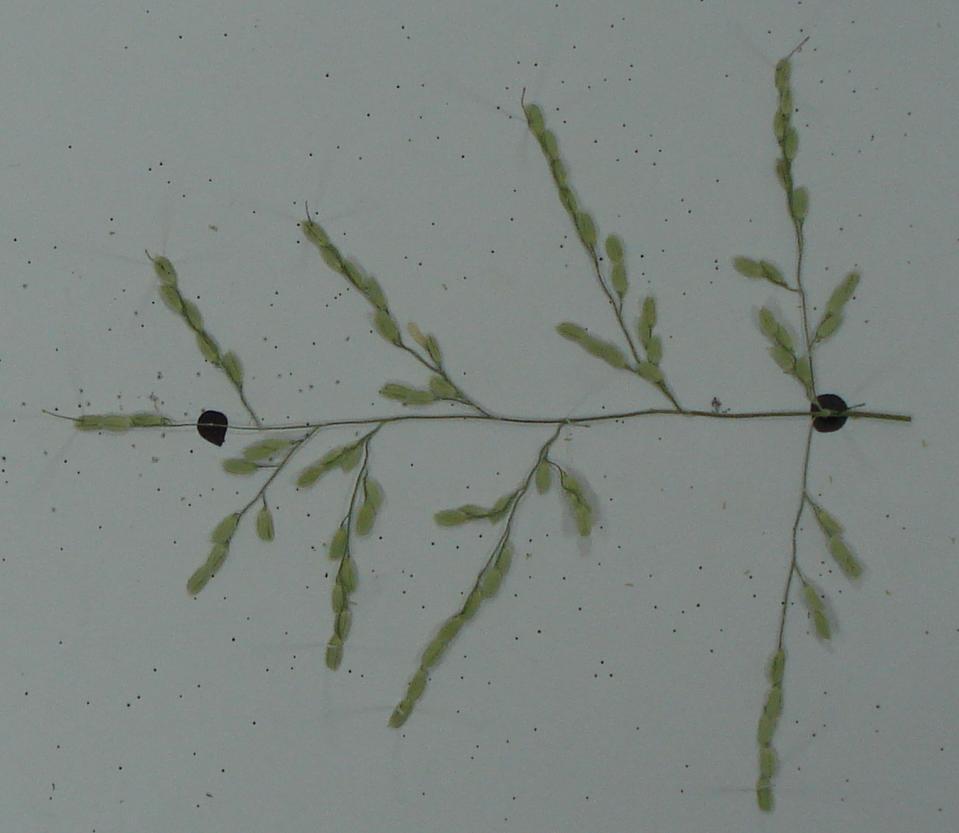

Supplement: Additional file 3 — 26 images of spread out panicles. A set of images of spread out panicles used to test the application for the detection of the structure, counting the grains or spikelets and for the detection of grain traits. [file 1471-2229-13-122-S3.zip › Additional file 3/38_2_2_2_2_DSC09932.JPG]

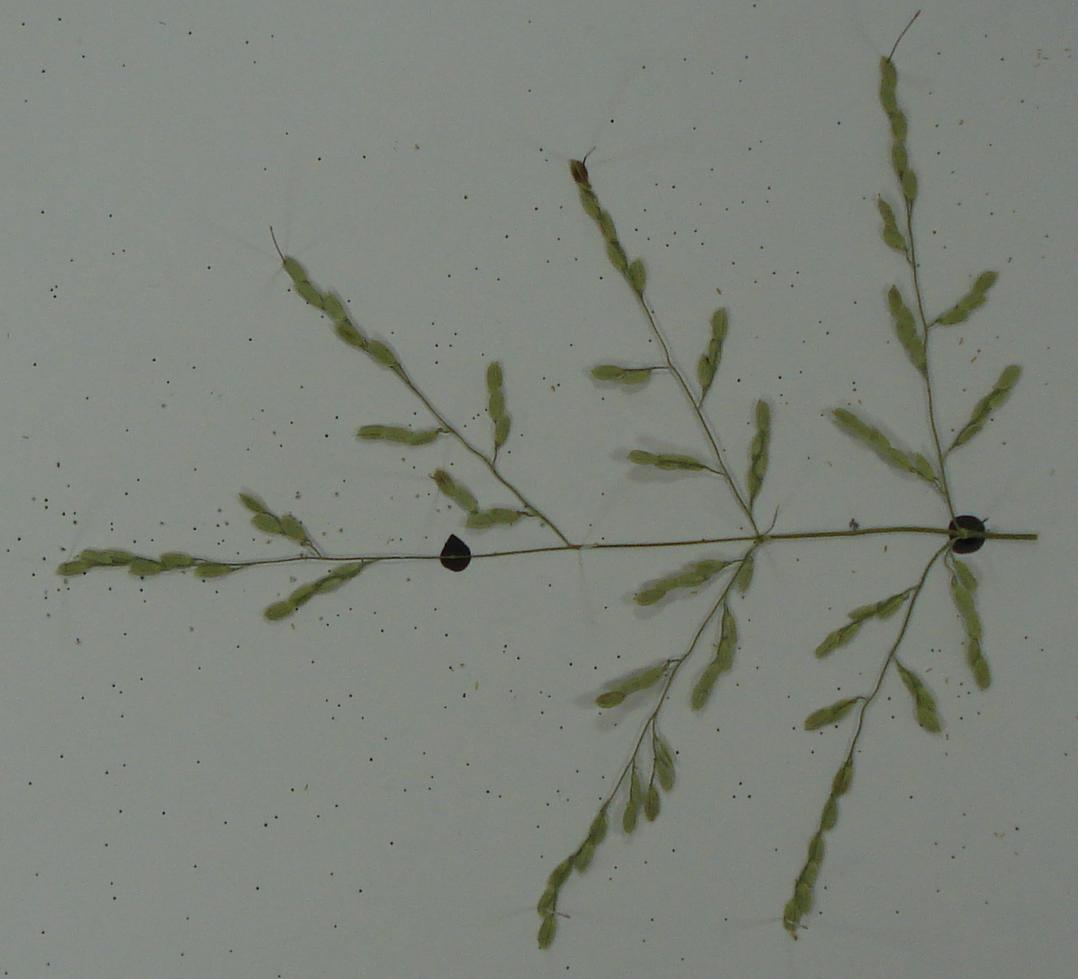

Supplement: Additional file 3 — 26 images of spread out panicles. A set of images of spread out panicles used to test the application for the detection of the structure, counting the grains or spikelets and for the detection of grain traits. [file 1471-2229-13-122-S3.zip › Additional file 3/38_2_2_3_2_DSC09934.JPG]

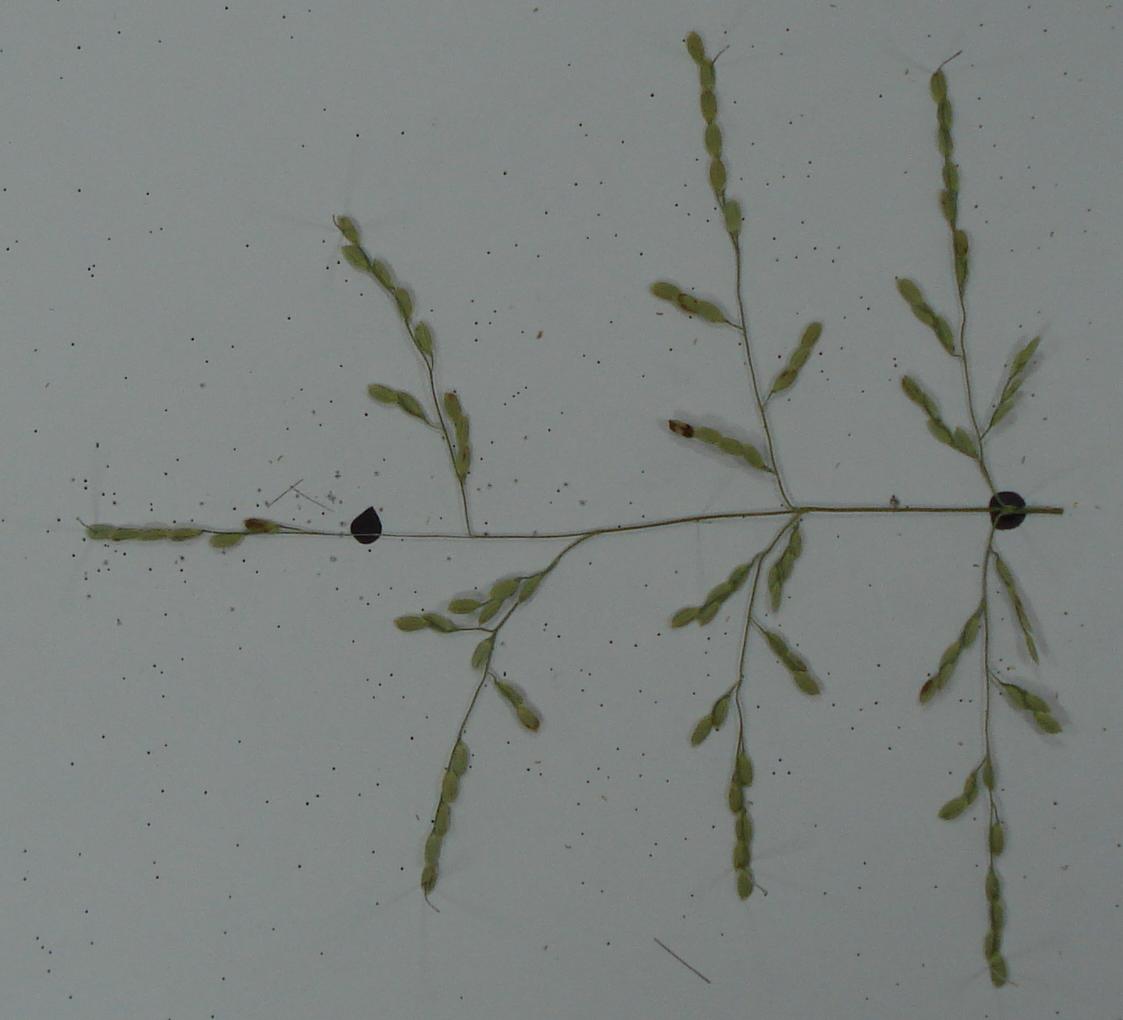

Supplement: Additional file 3 — 26 images of spread out panicles. A set of images of spread out panicles used to test the application for the detection of the structure, counting the grains or spikelets and for the detection of grain traits. [file 1471-2229-13-122-S3.zip › Additional file 3/38_2_2_3_3_DSC09935.JPG]

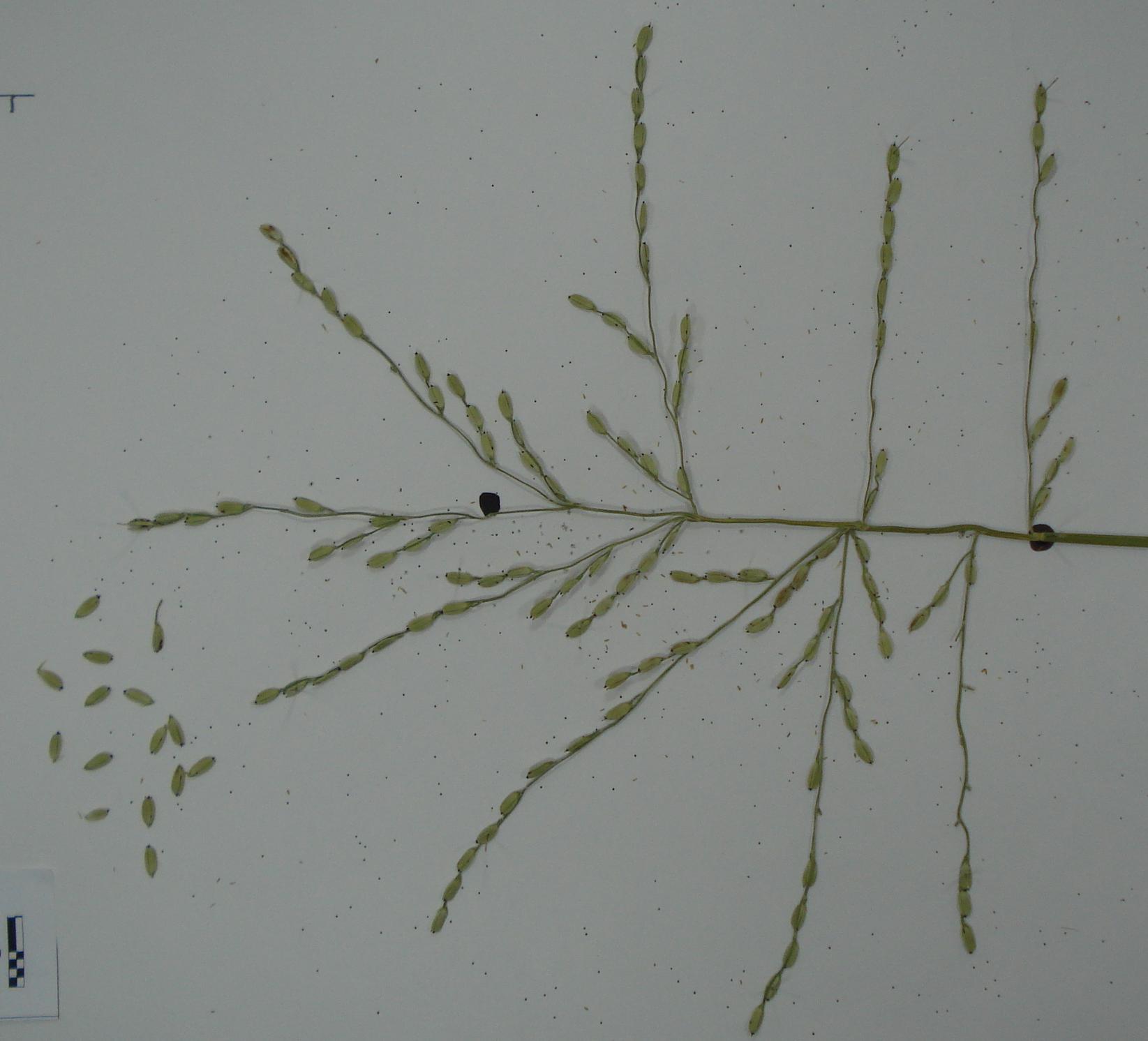

Supplement: Additional file 3 — 26 images of spread out panicles. A set of images of spread out panicles used to test the application for the detection of the structure, counting the grains or spikelets and for the detection of grain traits. [file 1471-2229-13-122-S3.zip › Additional file 3/7_2_1_1_2_DSC09916.JPG]

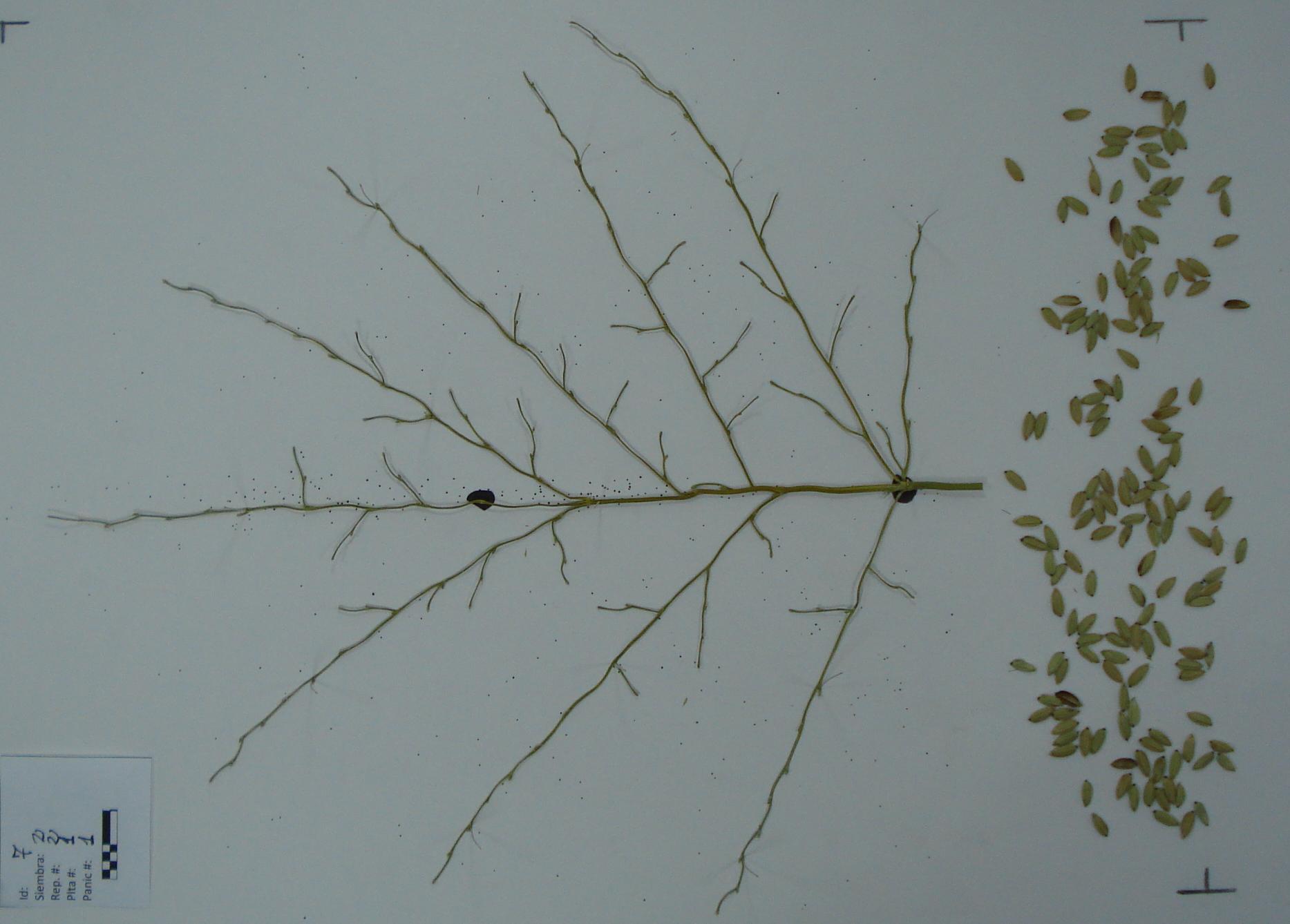

Supplement: Additional file 3 — 26 images of spread out panicles. A set of images of spread out panicles used to test the application for the detection of the structure, counting the grains or spikelets and for the detection of grain traits. [file 1471-2229-13-122-S3.zip › Additional file 3/7_2_2_1_1_DSC09906.JPG]

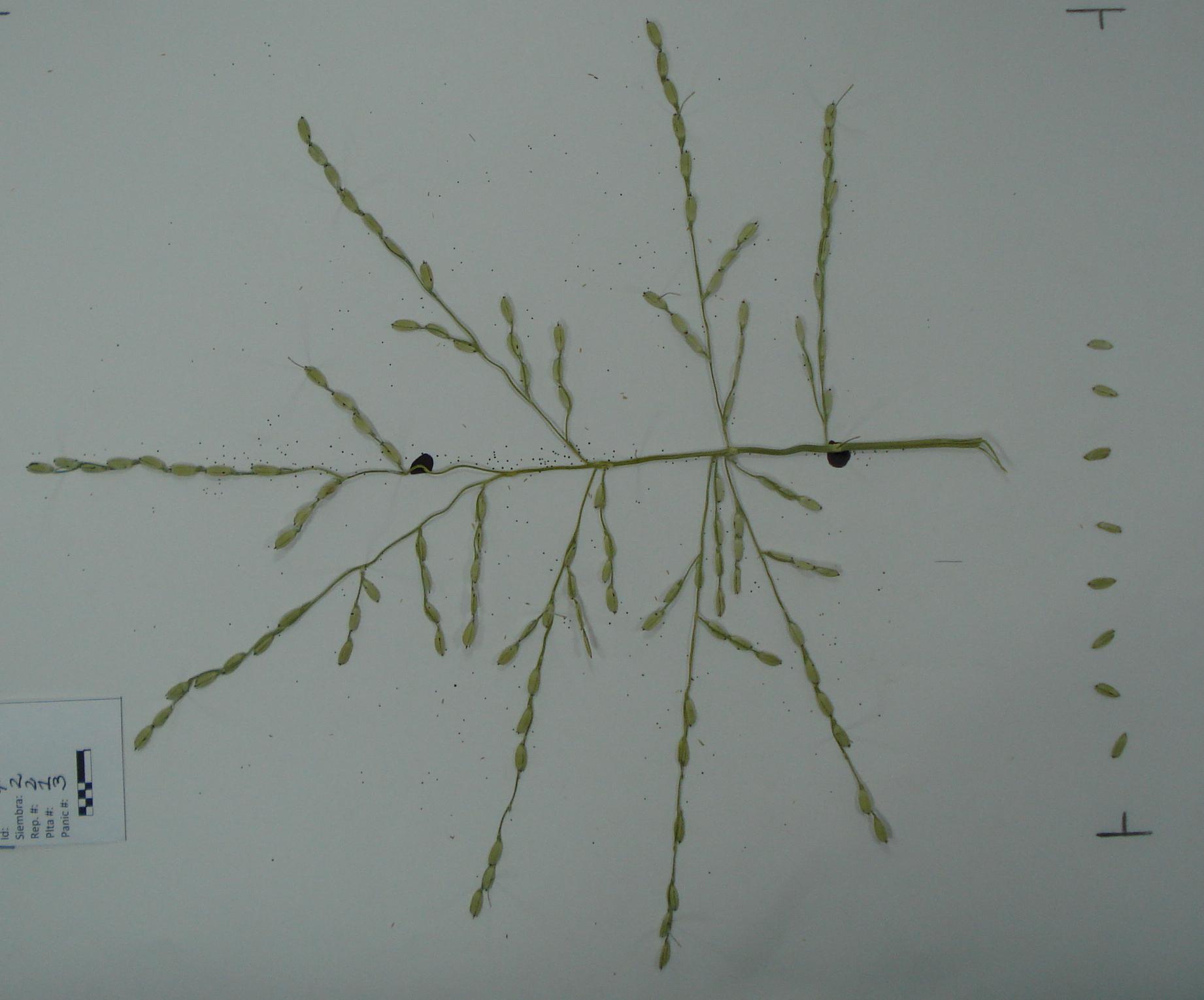

Supplement: Additional file 3 — 26 images of spread out panicles. A set of images of spread out panicles used to test the application for the detection of the structure, counting the grains or spikelets and for the detection of grain traits. [file 1471-2229-13-122-S3.zip › Additional file 3/7_2_2_1_3_DSC09907.JPG]

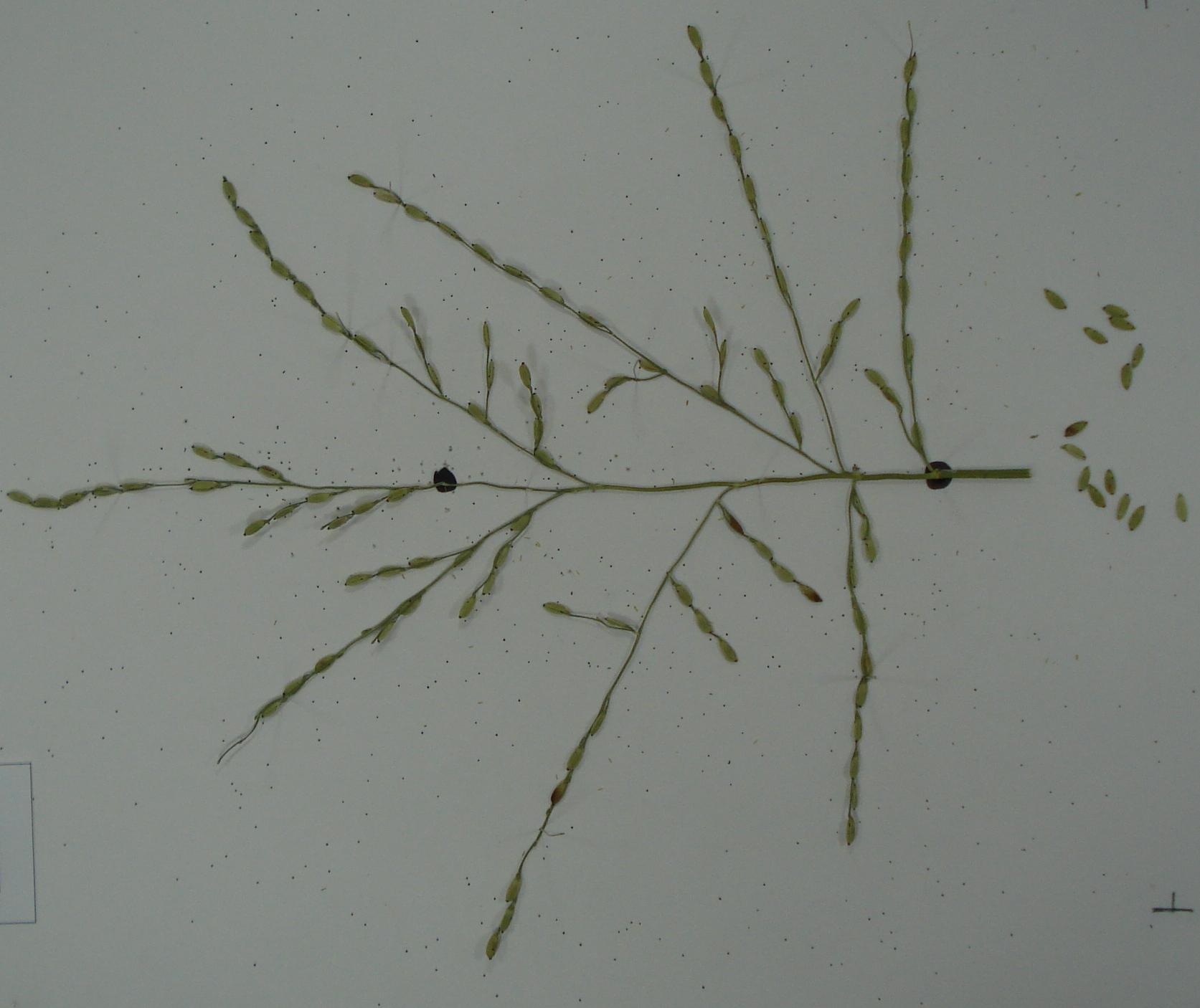

Supplement: Additional file 3 — 26 images of spread out panicles. A set of images of spread out panicles used to test the application for the detection of the structure, counting the grains or spikelets and for the detection of grain traits. [file 1471-2229-13-122-S3.zip › Additional file 3/8_2_1_1_1_DSC09947.JPG]

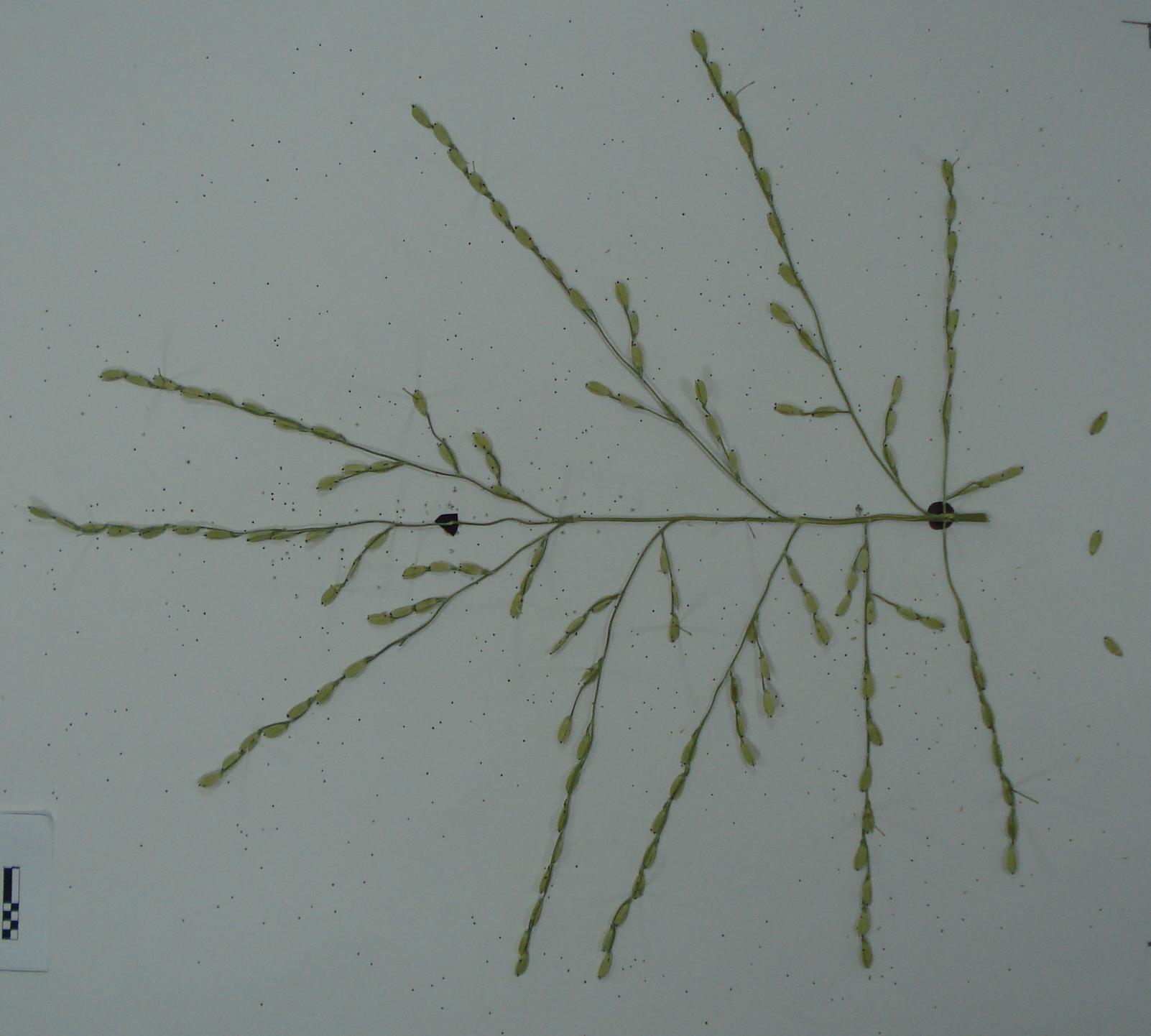

Supplement: Additional file 3 — 26 images of spread out panicles. A set of images of spread out panicles used to test the application for the detection of the structure, counting the grains or spikelets and for the detection of grain traits. [file 1471-2229-13-122-S3.zip › Additional file 3/8_2_1_2_1_DSC09953.JPG]

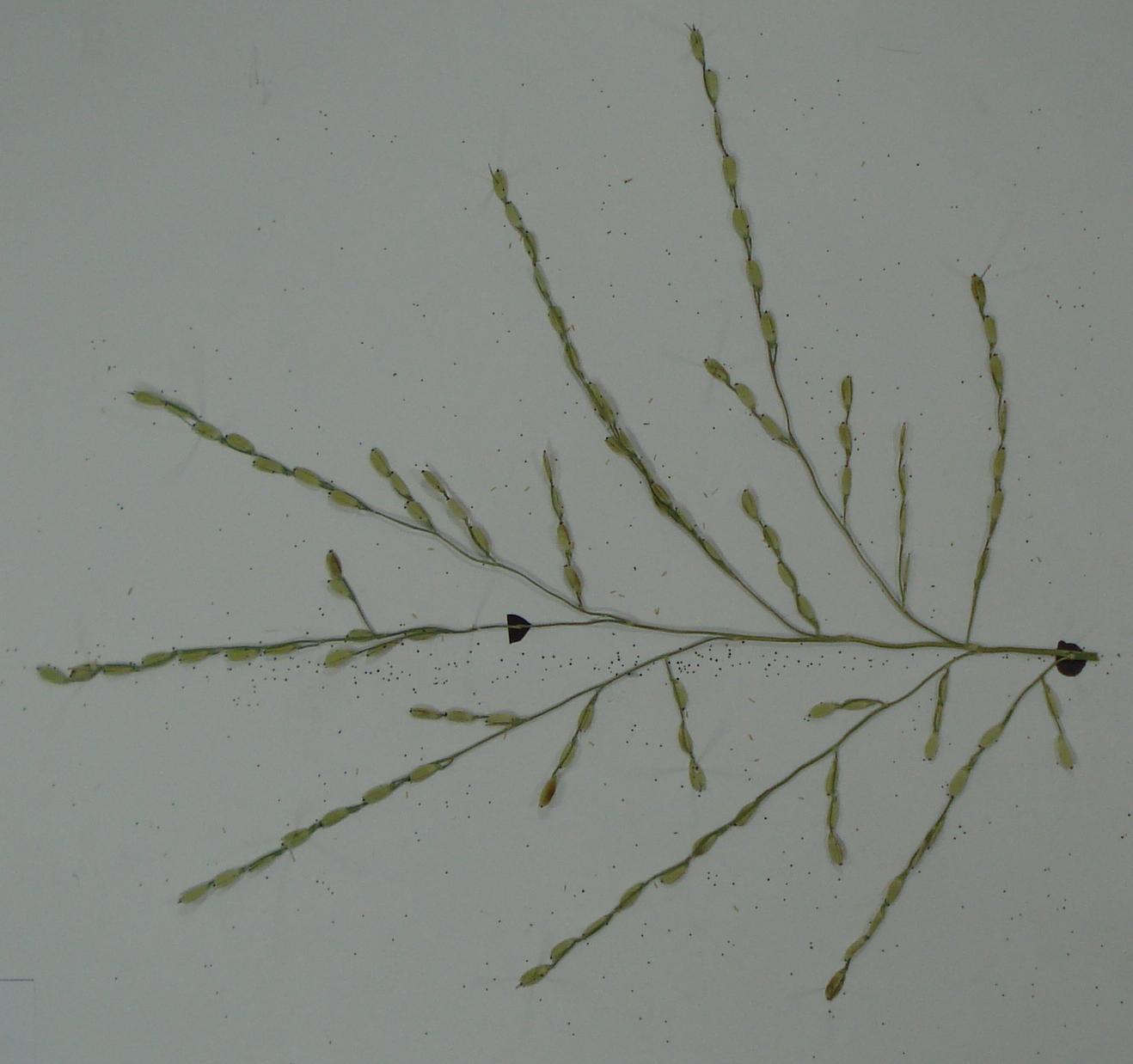

Supplement: Additional file 3 — 26 images of spread out panicles. A set of images of spread out panicles used to test the application for the detection of the structure, counting the grains or spikelets and for the detection of grain traits. [file 1471-2229-13-122-S3.zip › Additional file 3/8_2_1_3_1_DSC09945.JPG]

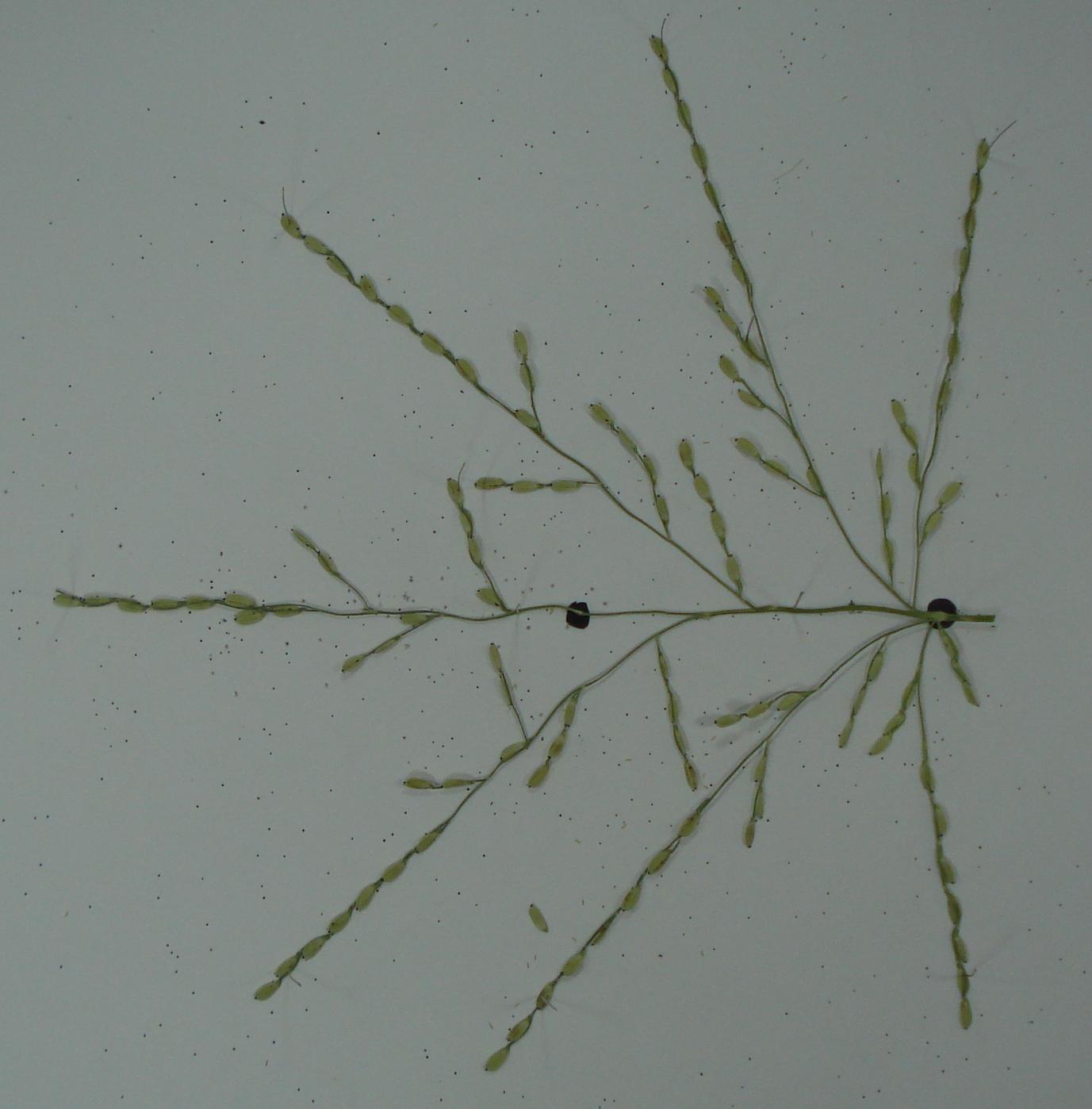

Supplement: Additional file 3 — 26 images of spread out panicles. A set of images of spread out panicles used to test the application for the detection of the structure, counting the grains or spikelets and for the detection of grain traits. [file 1471-2229-13-122-S3.zip › Additional file 3/8_2_1_3_3_DSC09946.JPG]

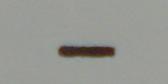

Supplement: Additional file 3 — 26 images of spread out panicles. A set of images of spread out panicles used to test the application for the detection of the structure, counting the grains or spikelets and for the detection of grain traits. [file 1471-2229-13-122-S3.zip › Additional file 3/scale.jpg]

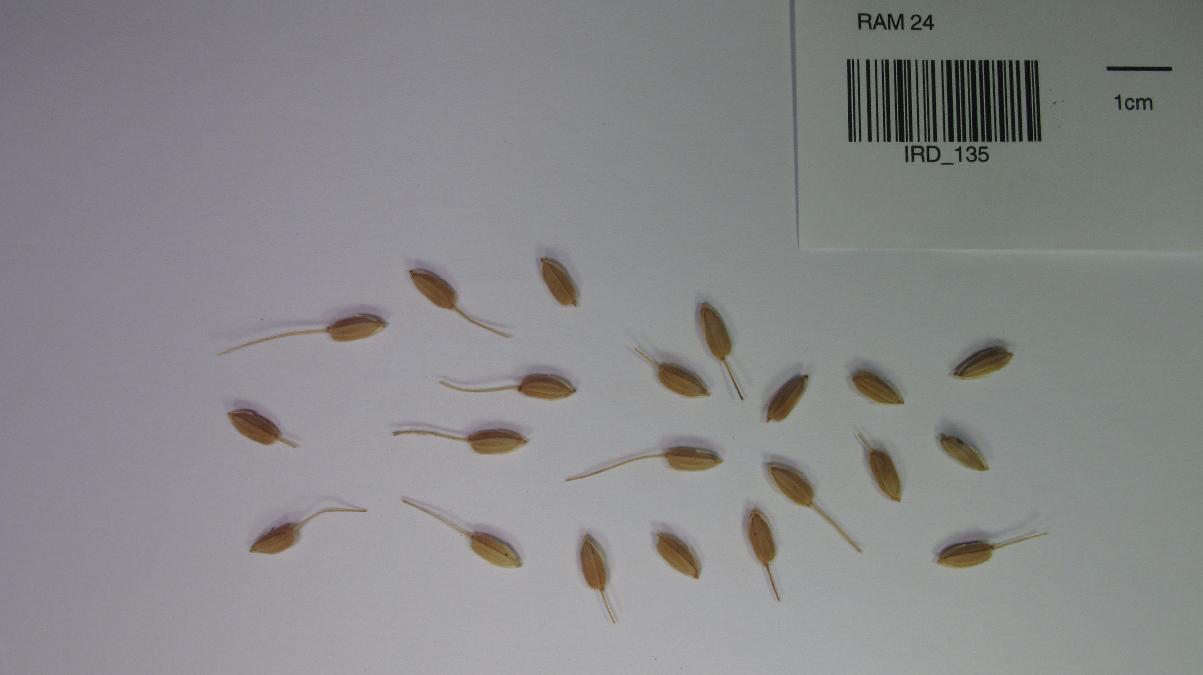

Supplement: Additional file 4 — 21 images of spread out seeds. A set of images of spread out grains used to test the application for the detection of grain traits. [file 1471-2229-13-122-S4.zip › Additional file 4/id 135e.jpg]

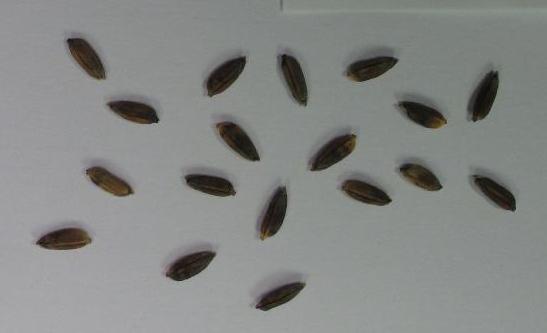

Supplement: Additional file 4 — 21 images of spread out seeds. A set of images of spread out grains used to test the application for the detection of grain traits. [file 1471-2229-13-122-S4.zip › Additional file 4/id 137e.jpg]

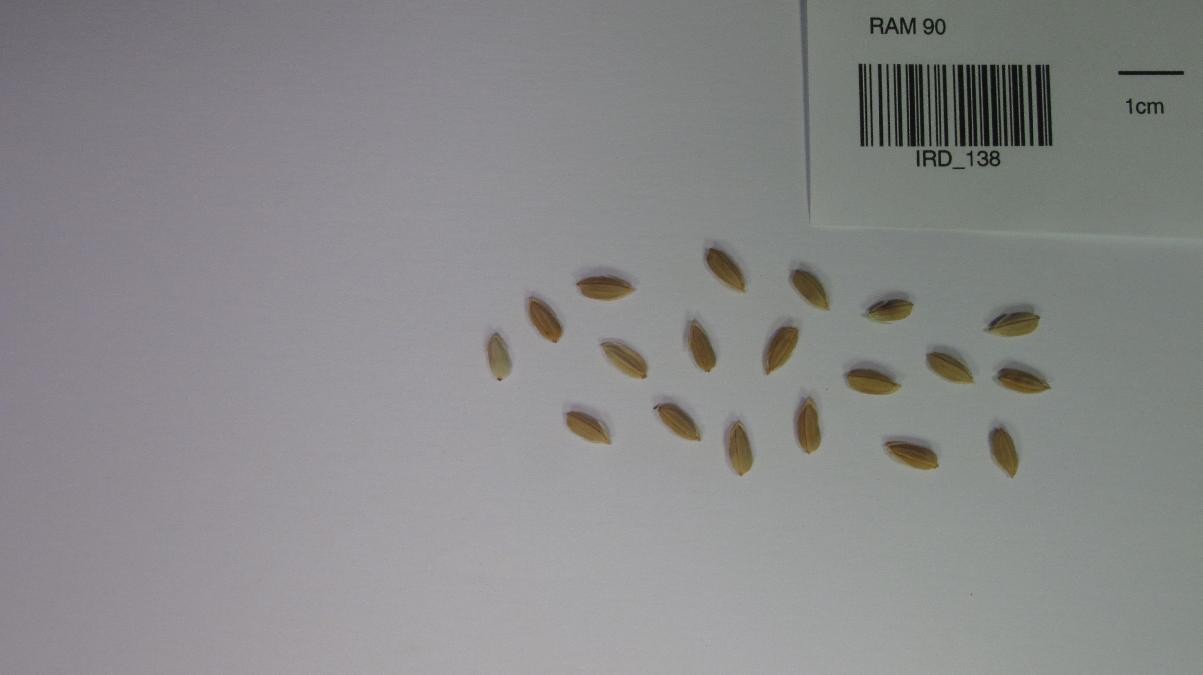

Supplement: Additional file 4 — 21 images of spread out seeds. A set of images of spread out grains used to test the application for the detection of grain traits. [file 1471-2229-13-122-S4.zip › Additional file 4/id 138e.jpg]

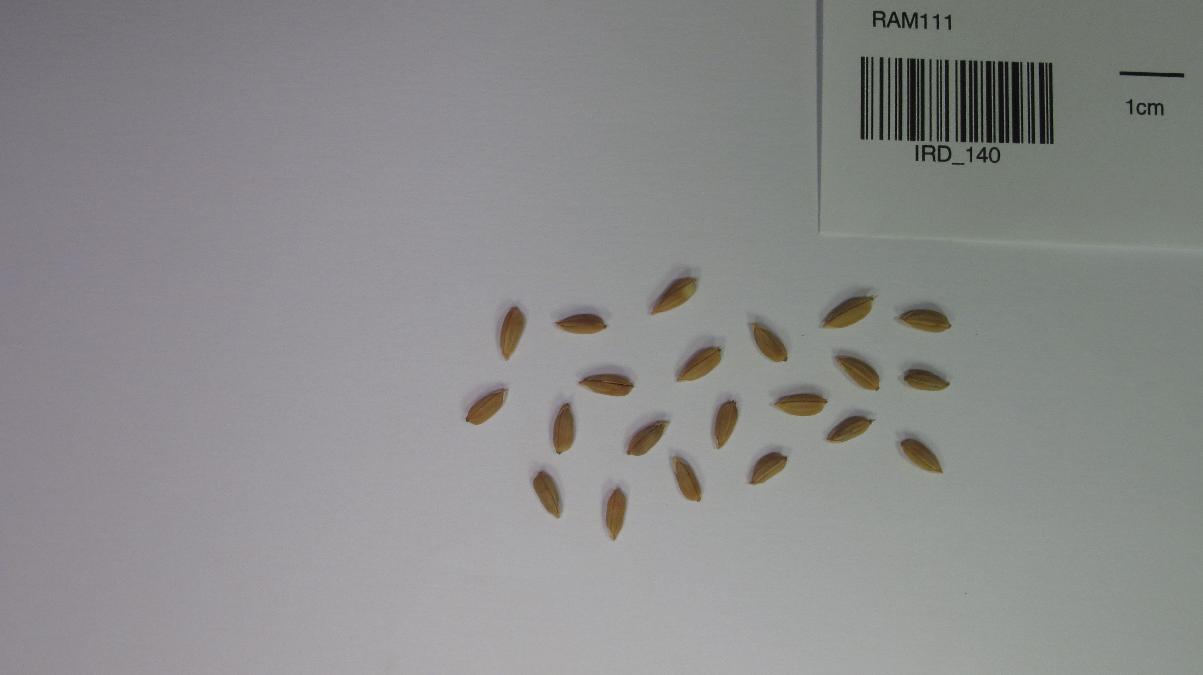

Supplement: Additional file 4 — 21 images of spread out seeds. A set of images of spread out grains used to test the application for the detection of grain traits. [file 1471-2229-13-122-S4.zip › Additional file 4/id 140e.jpg]

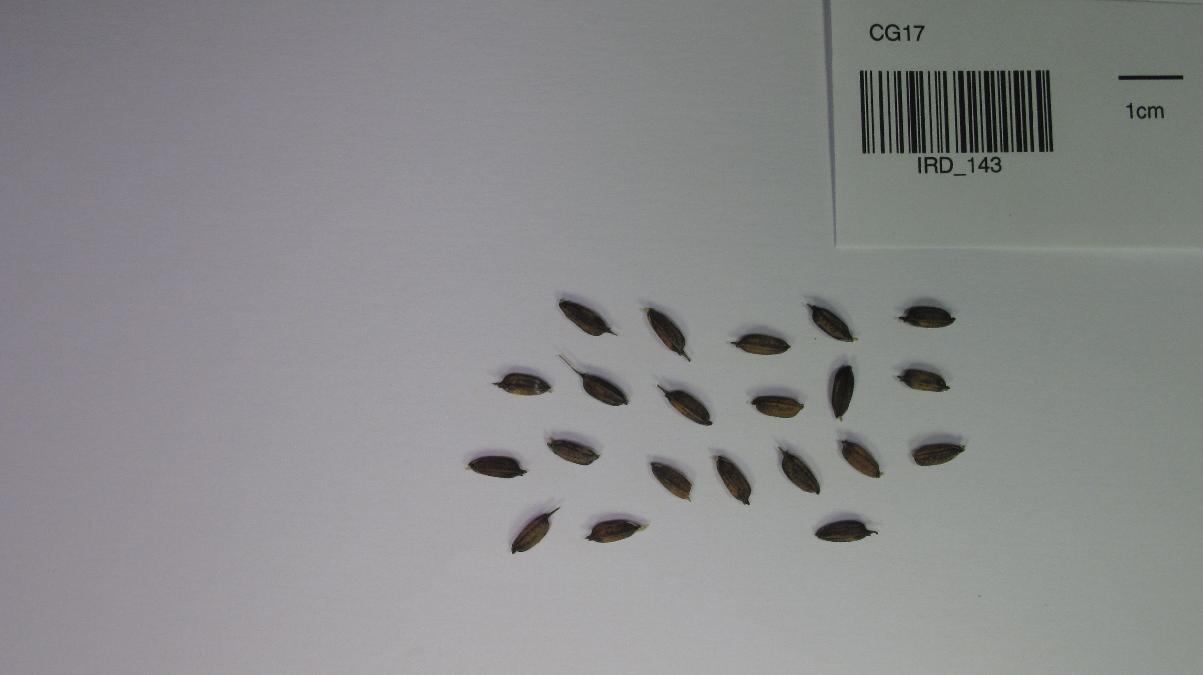

Supplement: Additional file 4 — 21 images of spread out seeds. A set of images of spread out grains used to test the application for the detection of grain traits. [file 1471-2229-13-122-S4.zip › Additional file 4/id 143e.jpg]

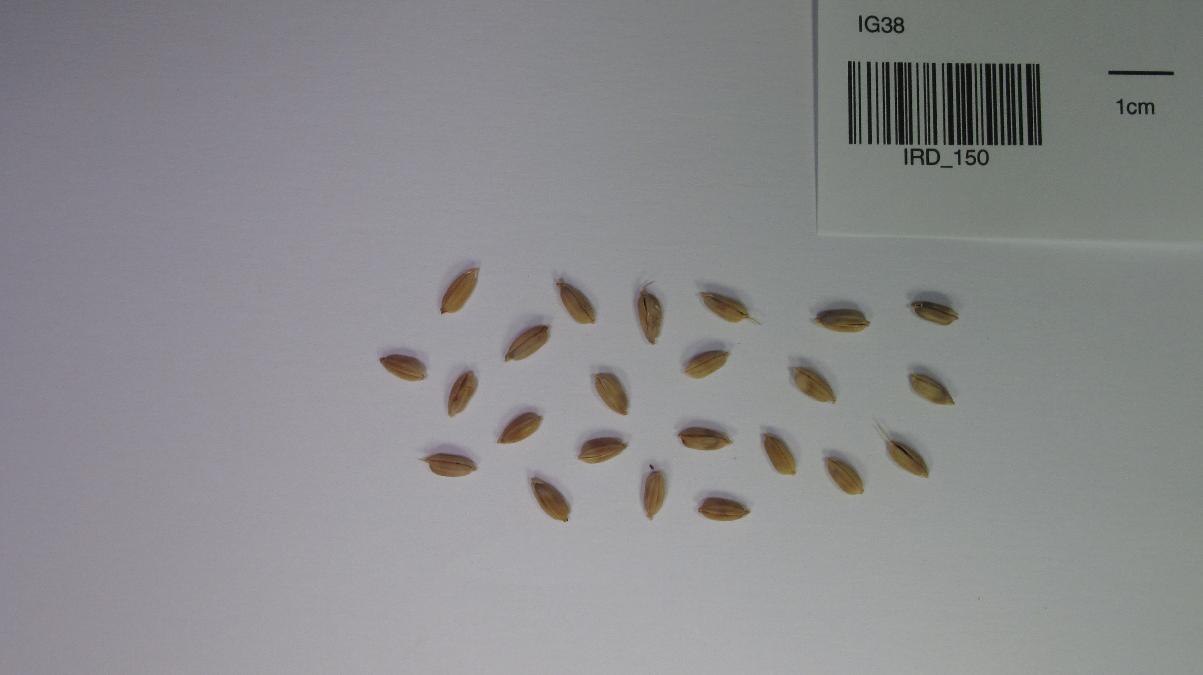

Supplement: Additional file 4 — 21 images of spread out seeds. A set of images of spread out grains used to test the application for the detection of grain traits. [file 1471-2229-13-122-S4.zip › Additional file 4/id 150e.jpg]

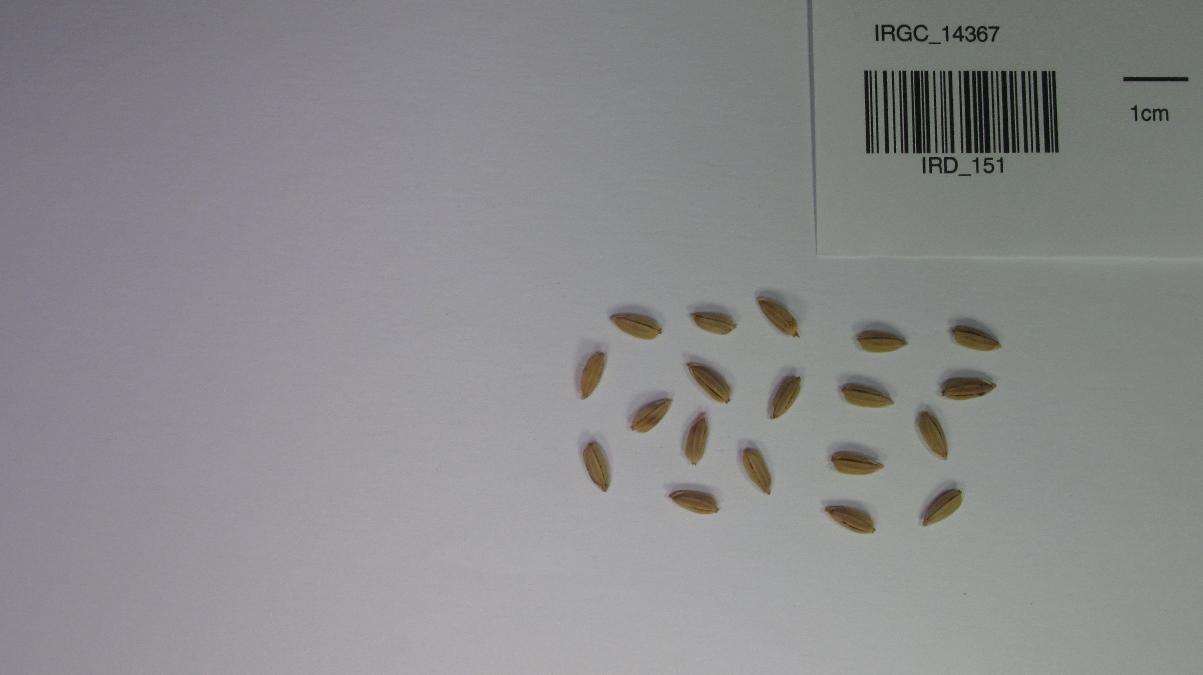

Supplement: Additional file 4 — 21 images of spread out seeds. A set of images of spread out grains used to test the application for the detection of grain traits. [file 1471-2229-13-122-S4.zip › Additional file 4/id 151e.jpg]

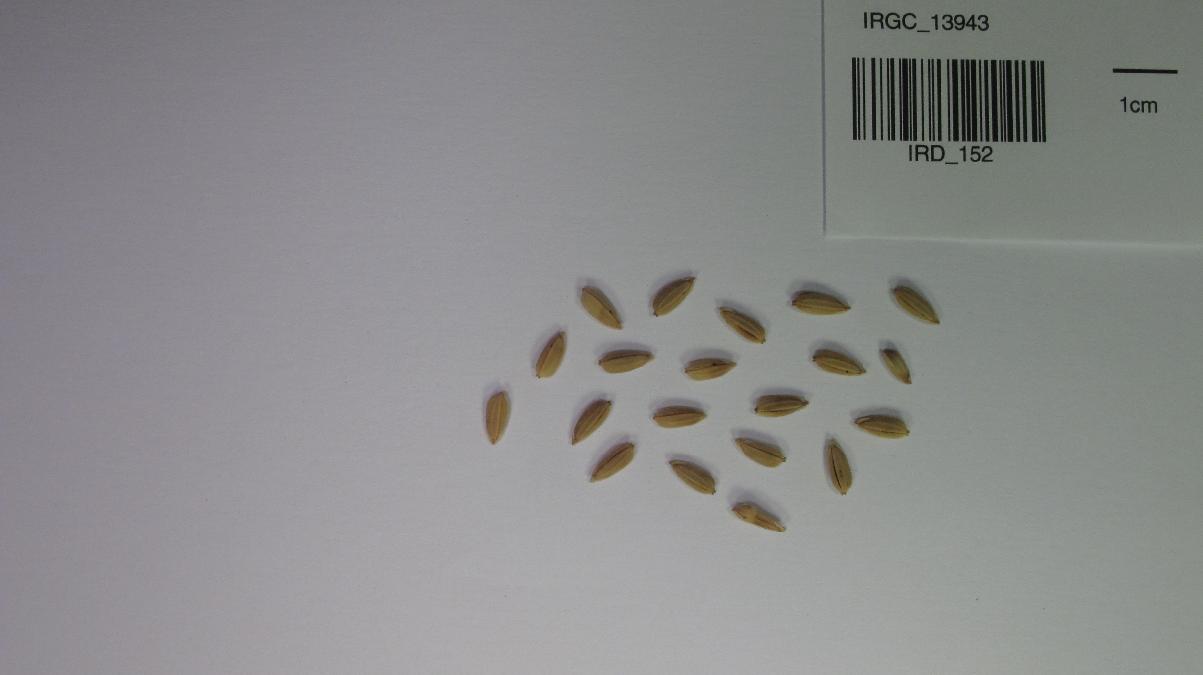

Supplement: Additional file 4 — 21 images of spread out seeds. A set of images of spread out grains used to test the application for the detection of grain traits. [file 1471-2229-13-122-S4.zip › Additional file 4/id 152e.jpg]

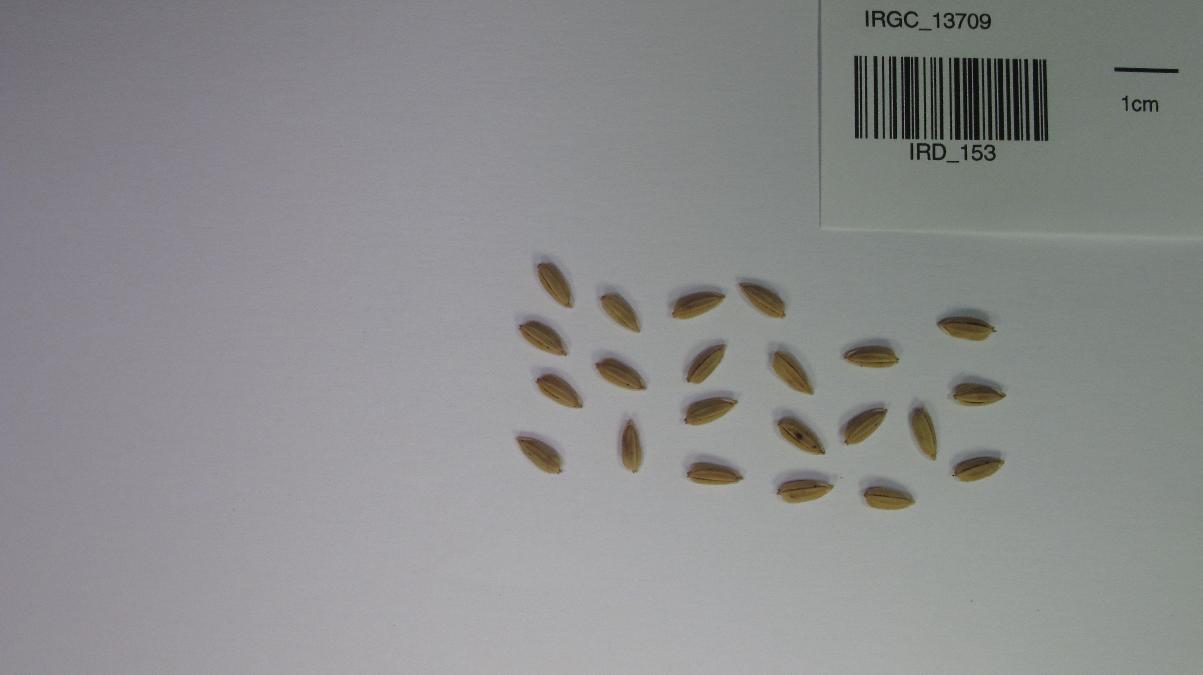

Supplement: Additional file 4 — 21 images of spread out seeds. A set of images of spread out grains used to test the application for the detection of grain traits. [file 1471-2229-13-122-S4.zip › Additional file 4/id 153e.jpg]

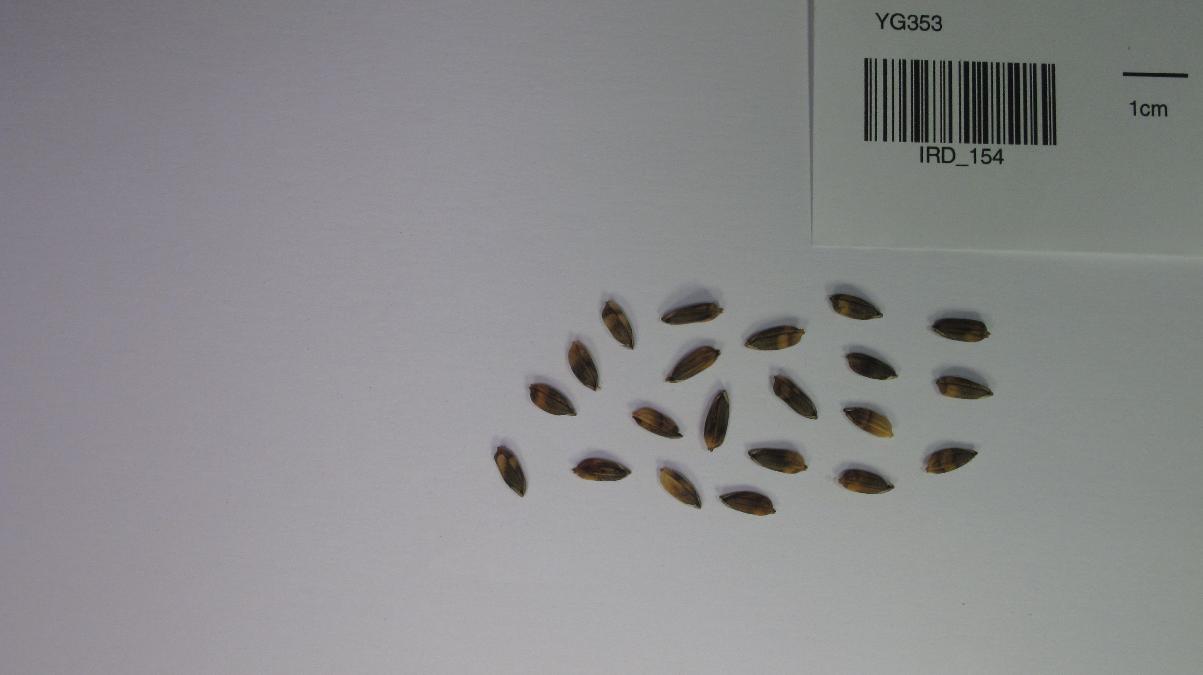

Supplement: Additional file 4 — 21 images of spread out seeds. A set of images of spread out grains used to test the application for the detection of grain traits. [file 1471-2229-13-122-S4.zip › Additional file 4/id 154e.jpg]

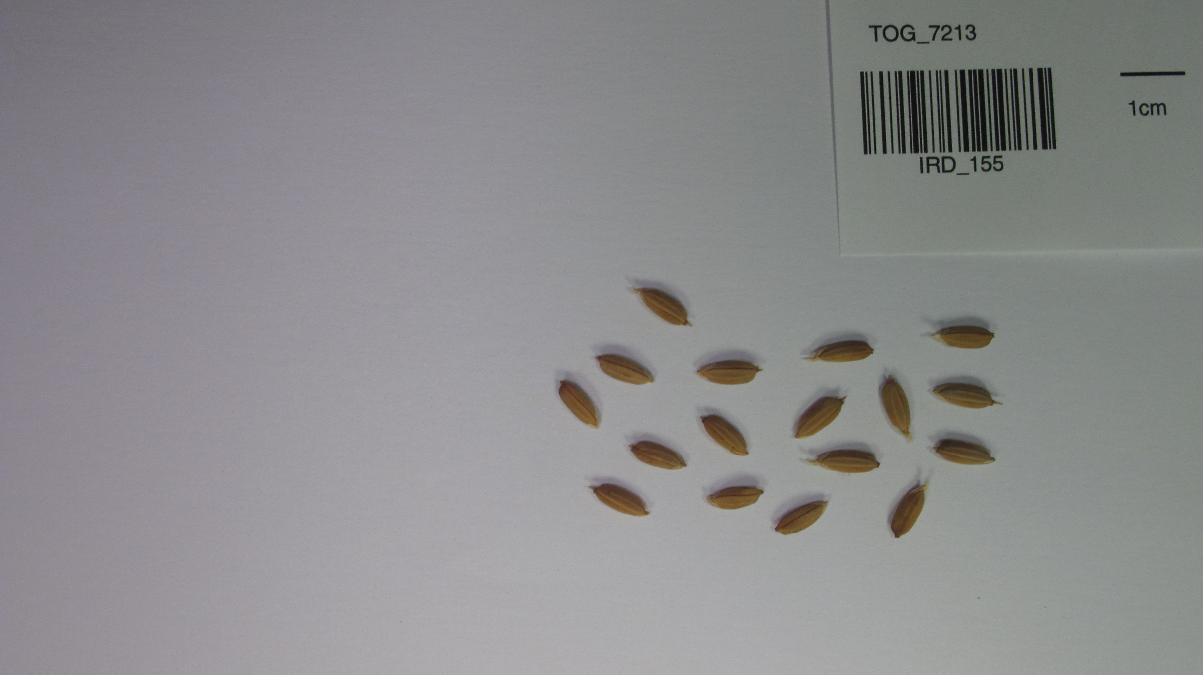

Supplement: Additional file 4 — 21 images of spread out seeds. A set of images of spread out grains used to test the application for the detection of grain traits. [file 1471-2229-13-122-S4.zip › Additional file 4/id 155e.jpg]

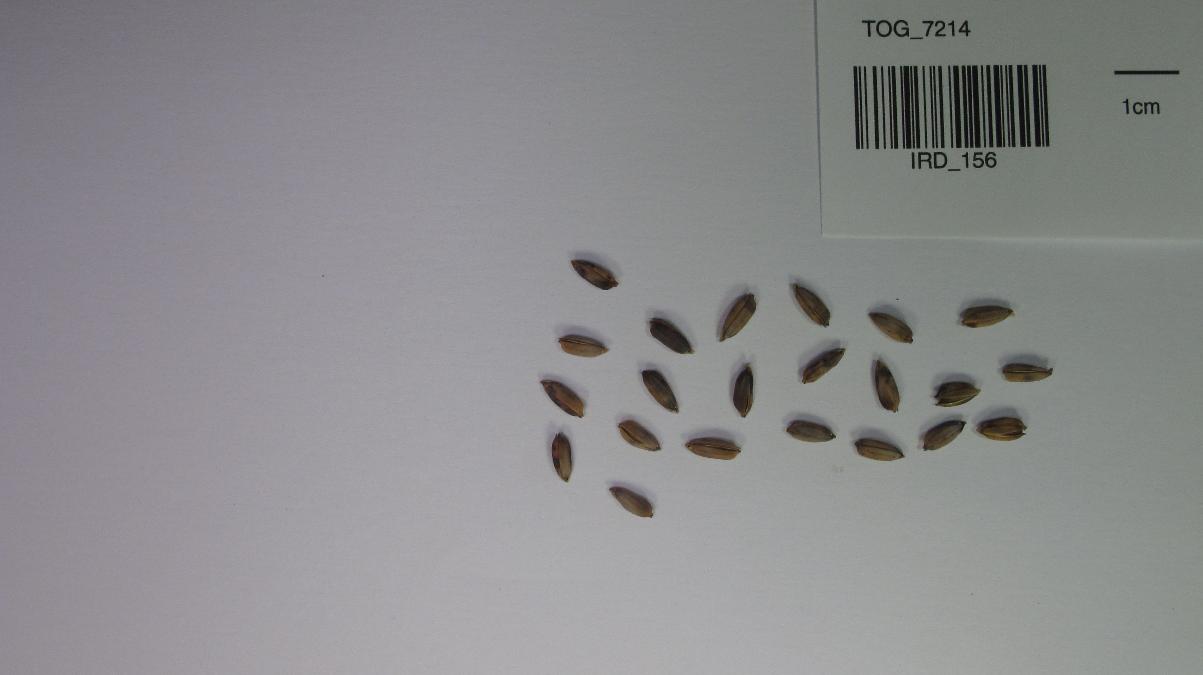

Supplement: Additional file 4 — 21 images of spread out seeds. A set of images of spread out grains used to test the application for the detection of grain traits. [file 1471-2229-13-122-S4.zip › Additional file 4/id 156e.jpg]

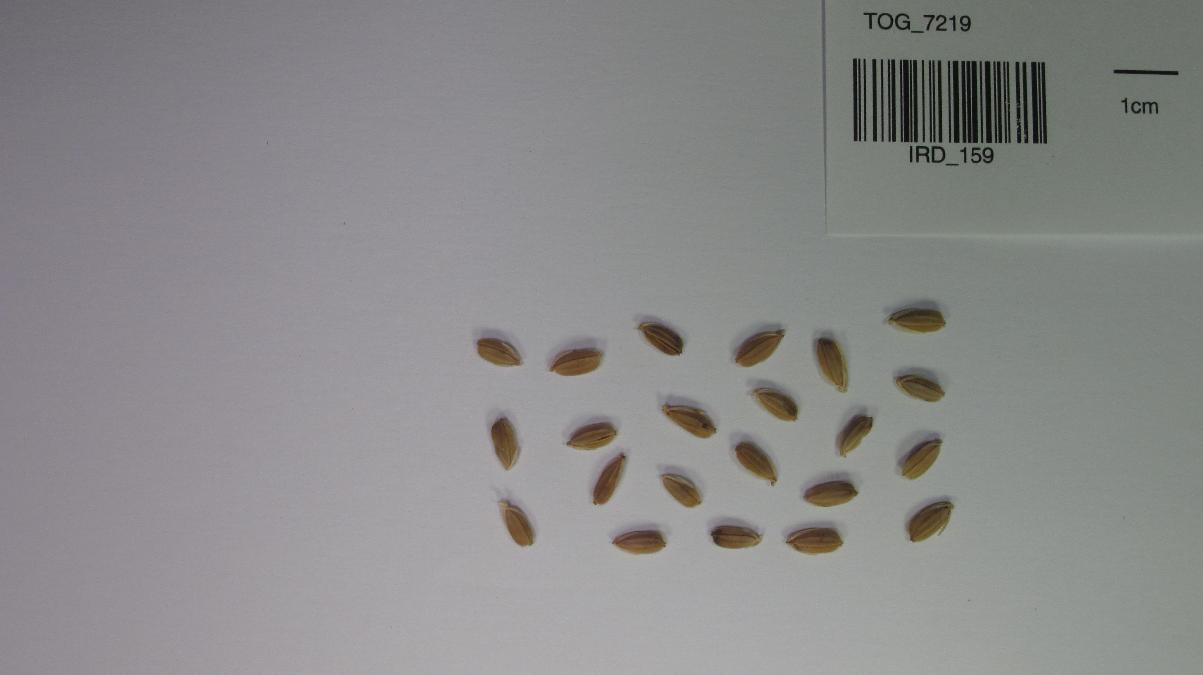

Supplement: Additional file 4 — 21 images of spread out seeds. A set of images of spread out grains used to test the application for the detection of grain traits. [file 1471-2229-13-122-S4.zip › Additional file 4/id 159e.jpg]

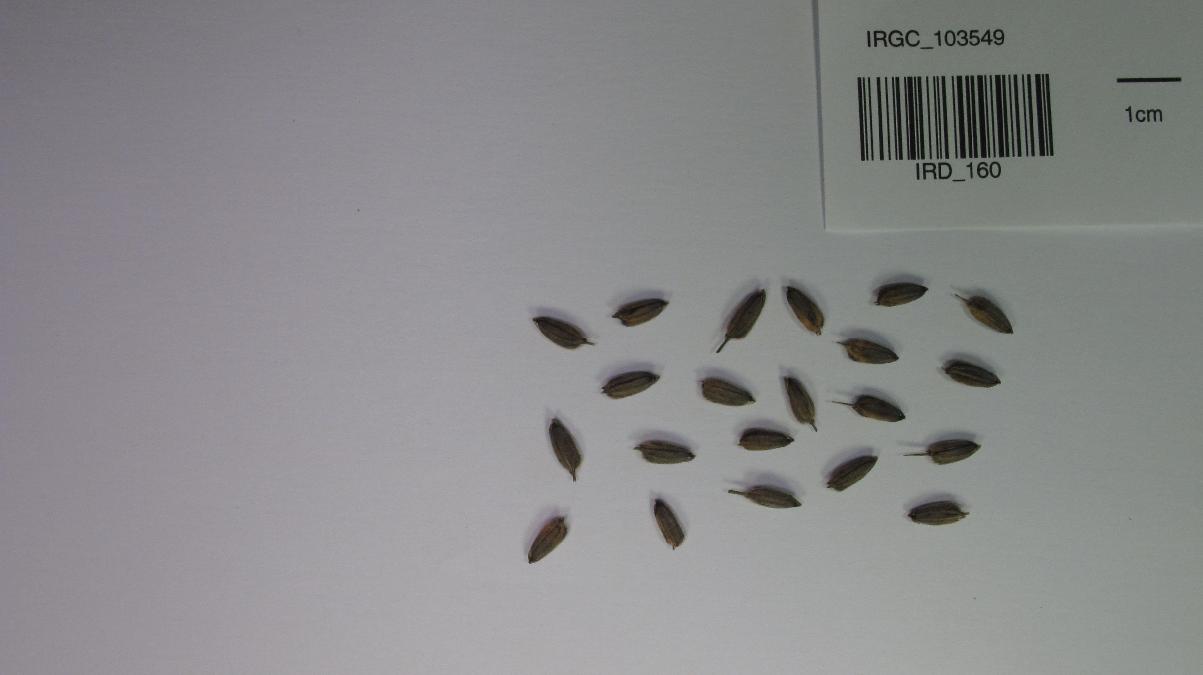

Supplement: Additional file 4 — 21 images of spread out seeds. A set of images of spread out grains used to test the application for the detection of grain traits. [file 1471-2229-13-122-S4.zip › Additional file 4/id 160e.jpg]

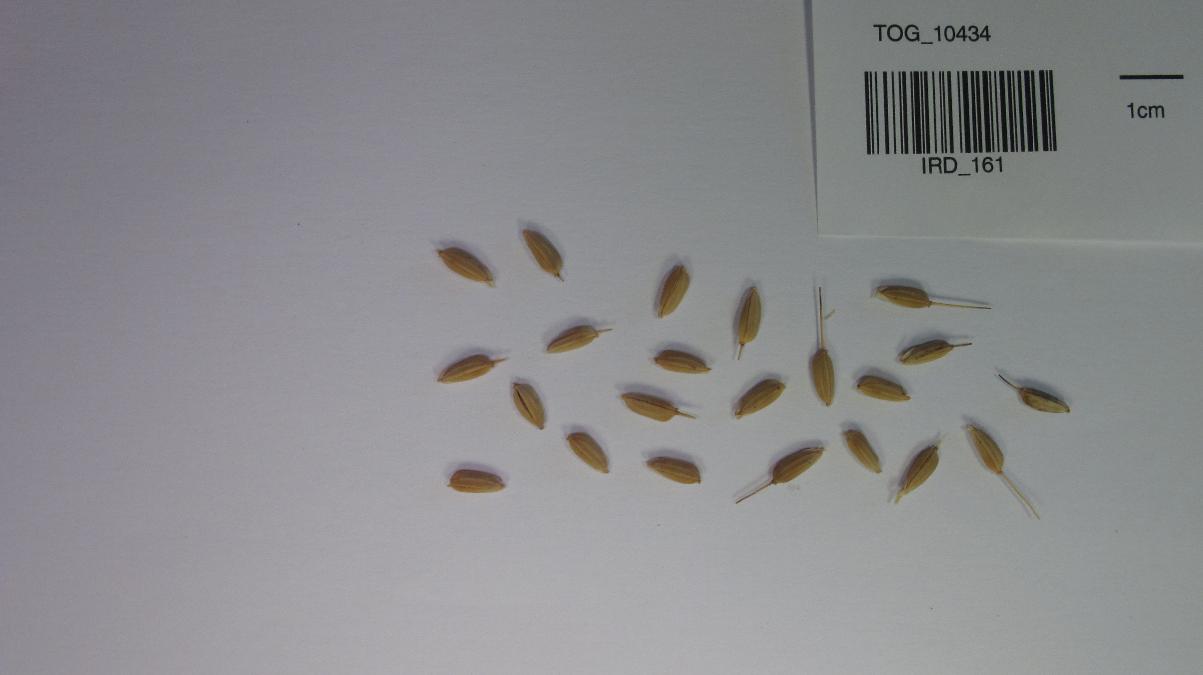

Supplement: Additional file 4 — 21 images of spread out seeds. A set of images of spread out grains used to test the application for the detection of grain traits. [file 1471-2229-13-122-S4.zip › Additional file 4/id 161e.jpg]

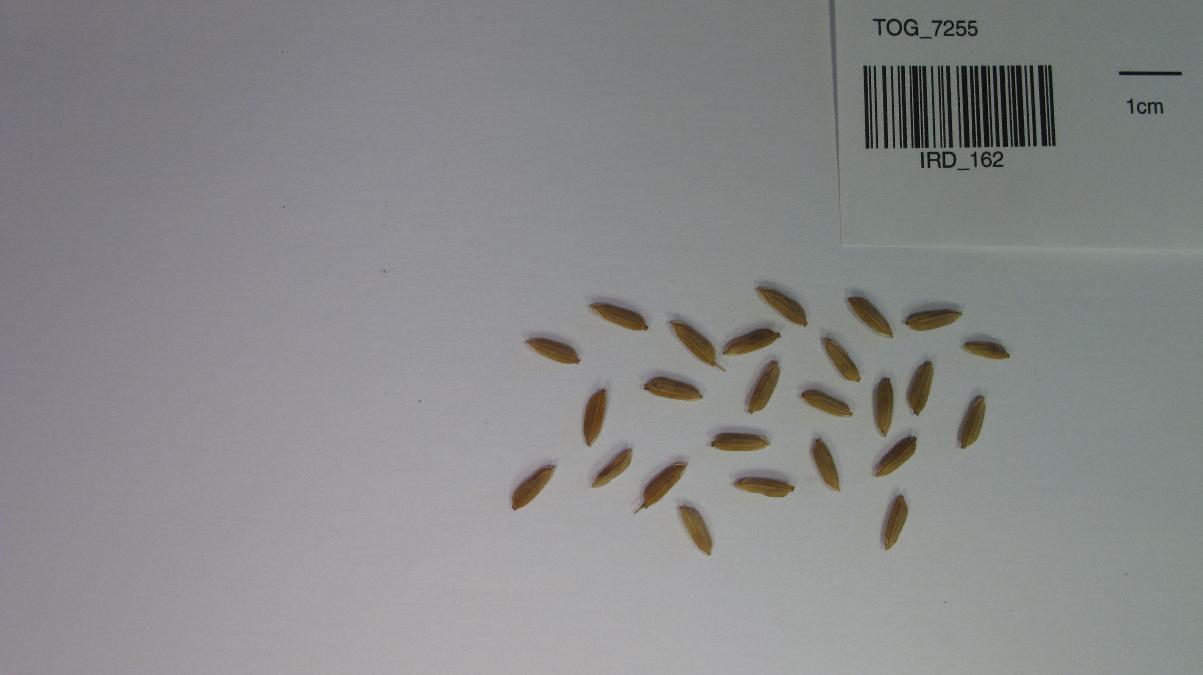

Supplement: Additional file 4 — 21 images of spread out seeds. A set of images of spread out grains used to test the application for the detection of grain traits. [file 1471-2229-13-122-S4.zip › Additional file 4/id 162e.jpg]

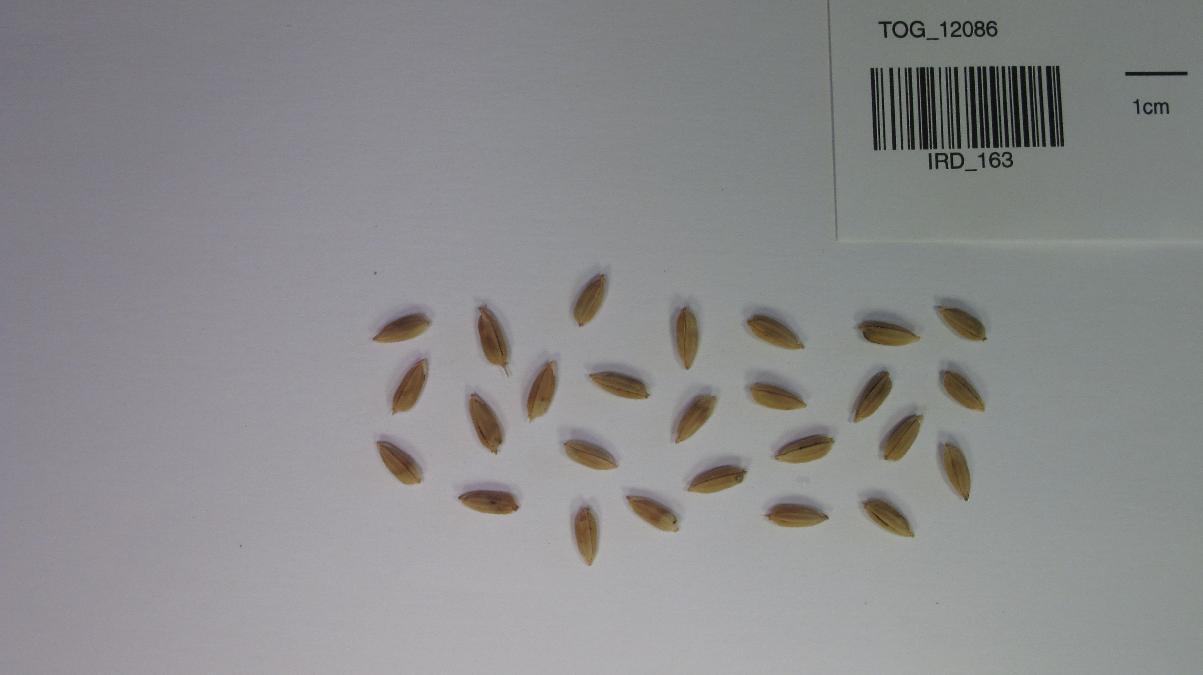

Supplement: Additional file 4 — 21 images of spread out seeds. A set of images of spread out grains used to test the application for the detection of grain traits. [file 1471-2229-13-122-S4.zip › Additional file 4/id 163e.jpg]

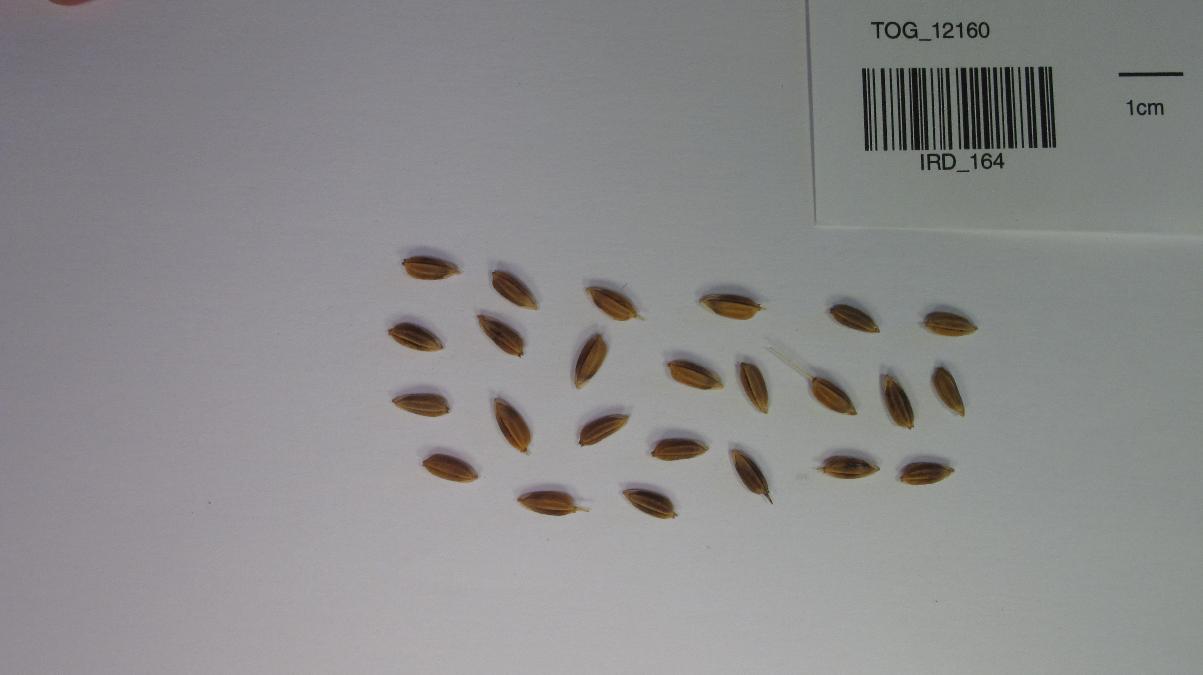

Supplement: Additional file 4 — 21 images of spread out seeds. A set of images of spread out grains used to test the application for the detection of grain traits. [file 1471-2229-13-122-S4.zip › Additional file 4/id 164e.jpg]

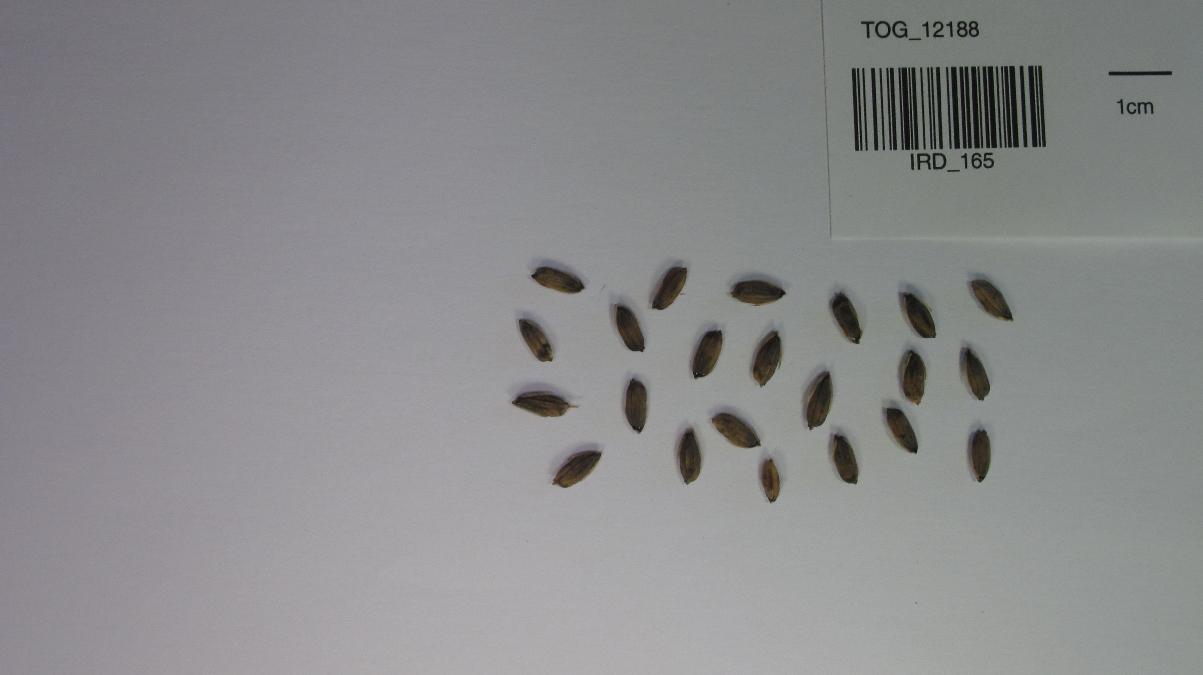

Supplement: Additional file 4 — 21 images of spread out seeds. A set of images of spread out grains used to test the application for the detection of grain traits. [file 1471-2229-13-122-S4.zip › Additional file 4/id 165e.jpg]

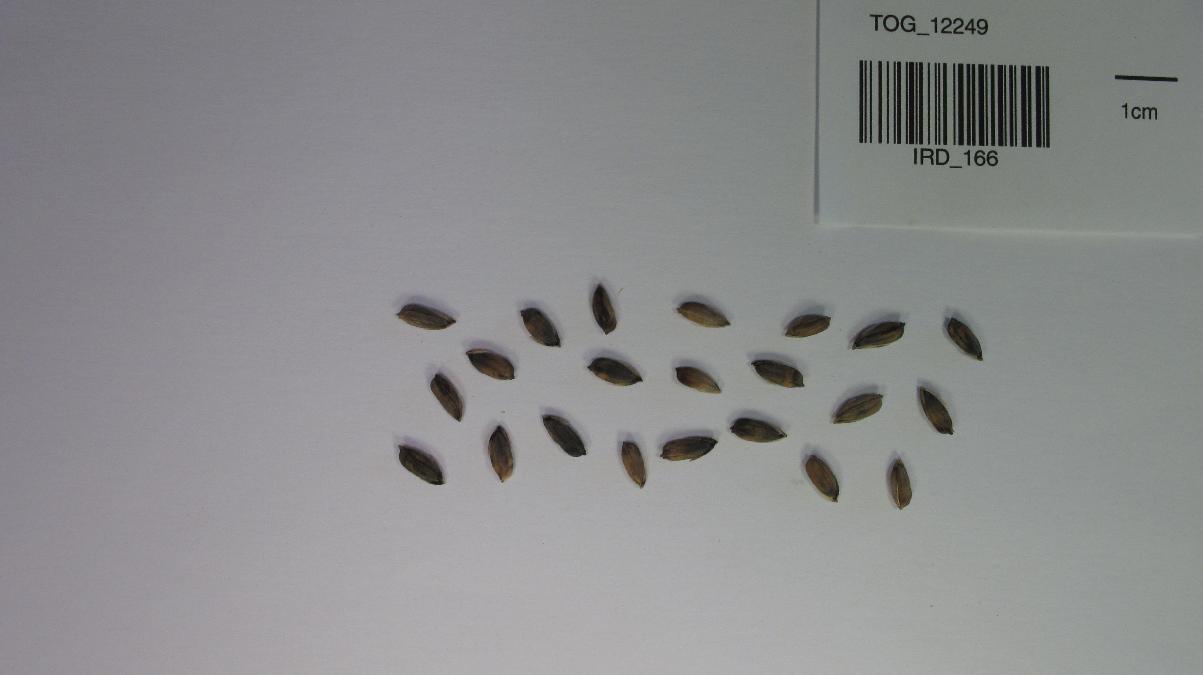

Supplement: Additional file 4 — 21 images of spread out seeds. A set of images of spread out grains used to test the application for the detection of grain traits. [file 1471-2229-13-122-S4.zip › Additional file 4/id 166e.jpg]

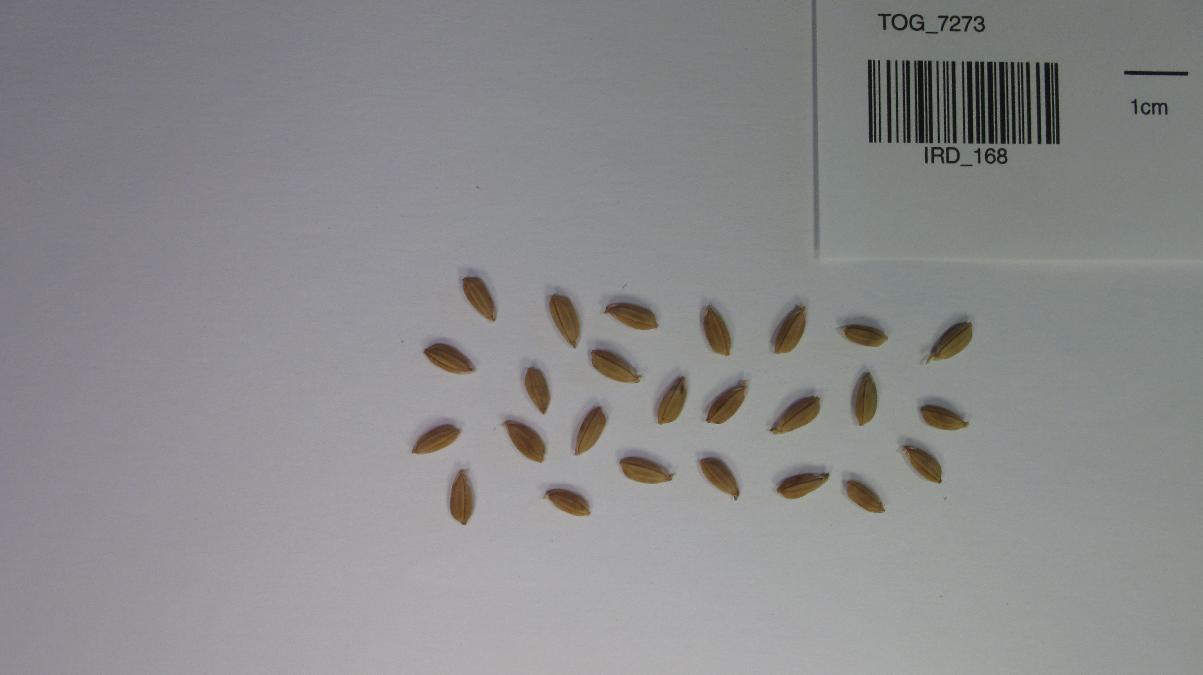

Supplement: Additional file 4 — 21 images of spread out seeds. A set of images of spread out grains used to test the application for the detection of grain traits. [file 1471-2229-13-122-S4.zip › Additional file 4/id 168e.jpg]
