# Supplementary material for: P-TRAP: a Panicle Trait Phenotyping tool
Source: BMC Plant Biol. 2013 Aug 29;13:122. doi: 10.1186/1471-2229-13-122 (PMC3848748; doi:10.1186/1471-2229-13-122)
Supplement: Additional file 5 — Example of overlapping grains. Samples with extremely overlapped grains. [file 1471-2229-13-122-S5.pdf]

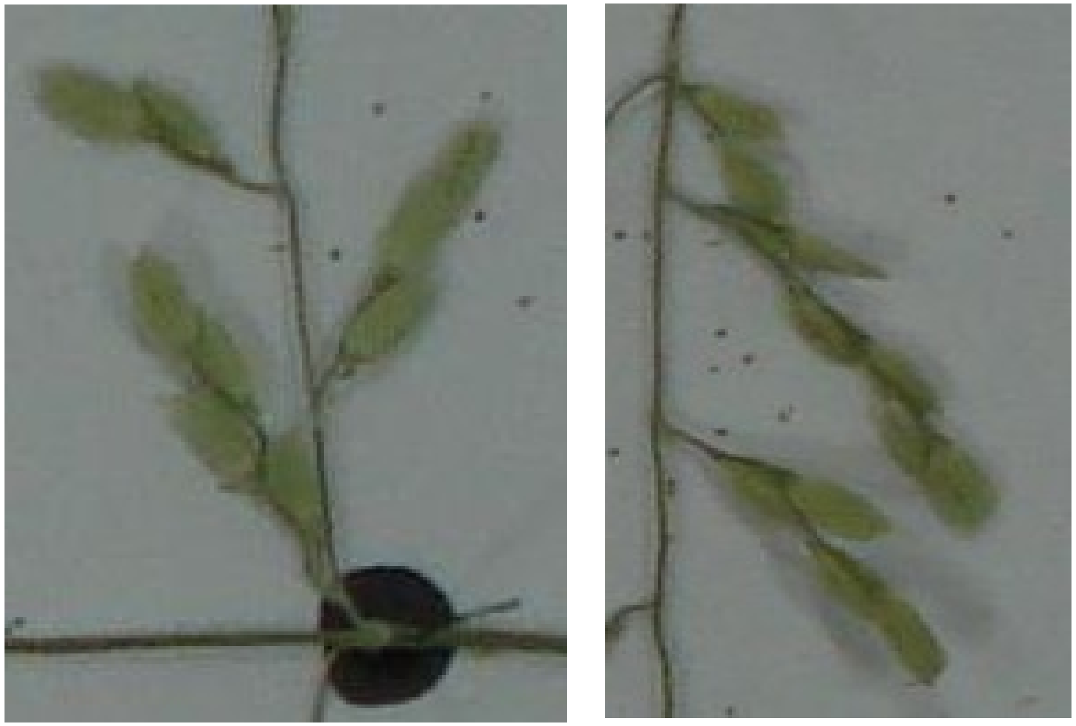

Overlapped branches samples

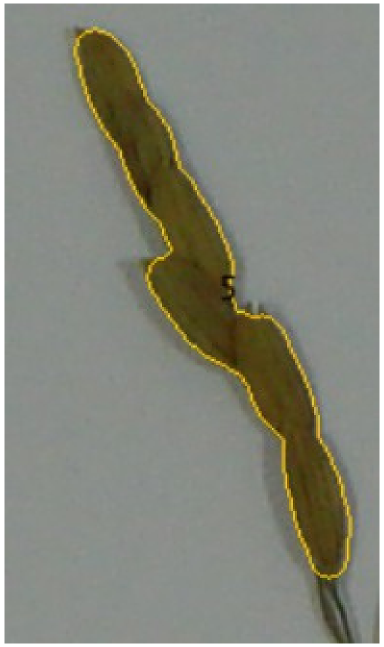

P-TRAP under-estimation of the number of grains due to overlapping
