# Supplementary material for: P-TRAP: a Panicle Trait Phenotyping tool
Source: BMC Plant Biol. 2013 Aug 29;13:122. doi: 10.1186/1471-2229-13-122 (PMC3848748; doi:10.1186/1471-2229-13-122)
Supplement: Additional file 6 — Sample images processed by P-TRAP and by other approaches. Several different images processed by P-TRAP and by other approaches. [file 1471-2229-13-122-S6.pdf]

Sample Images Processed by P-TRAP and Some Other Approaches

1- The Oversegmentation Problem of the Watershed Transform.

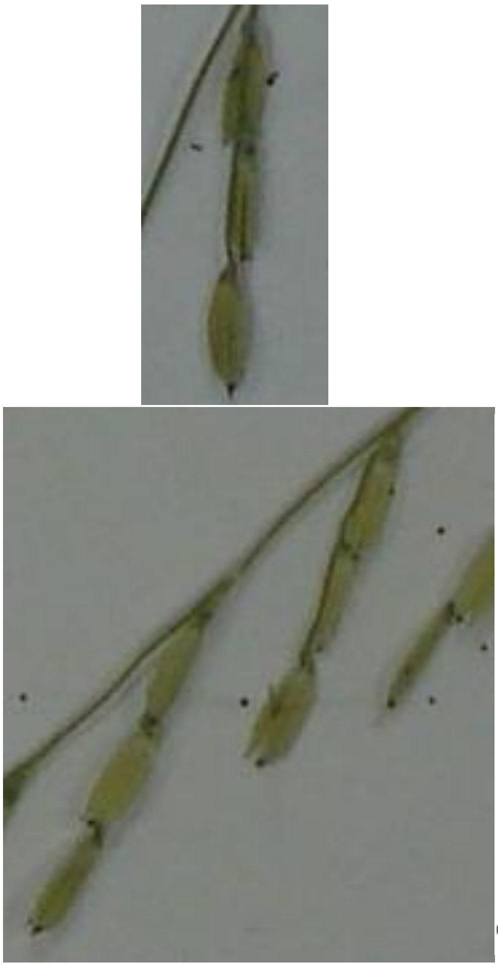

Sample images

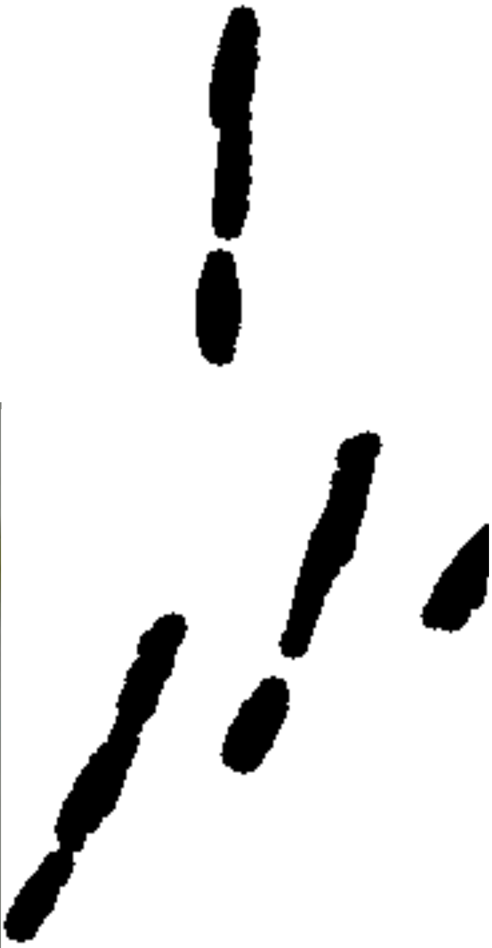

After applying the mathematical opening to the binary version of the sample with a disk of the grain size

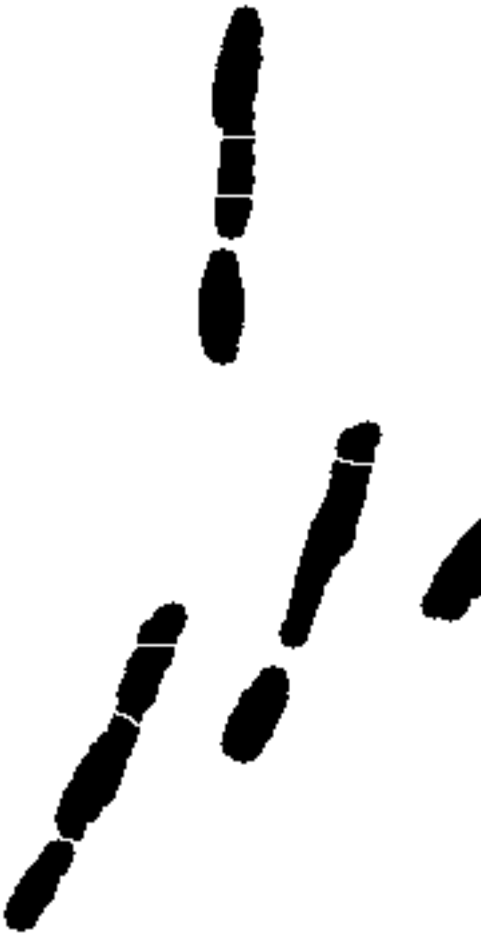

After applying the watershed transform to the openned image.

2- P-TRAP Grains Detection for both On-branch and Spread Grains

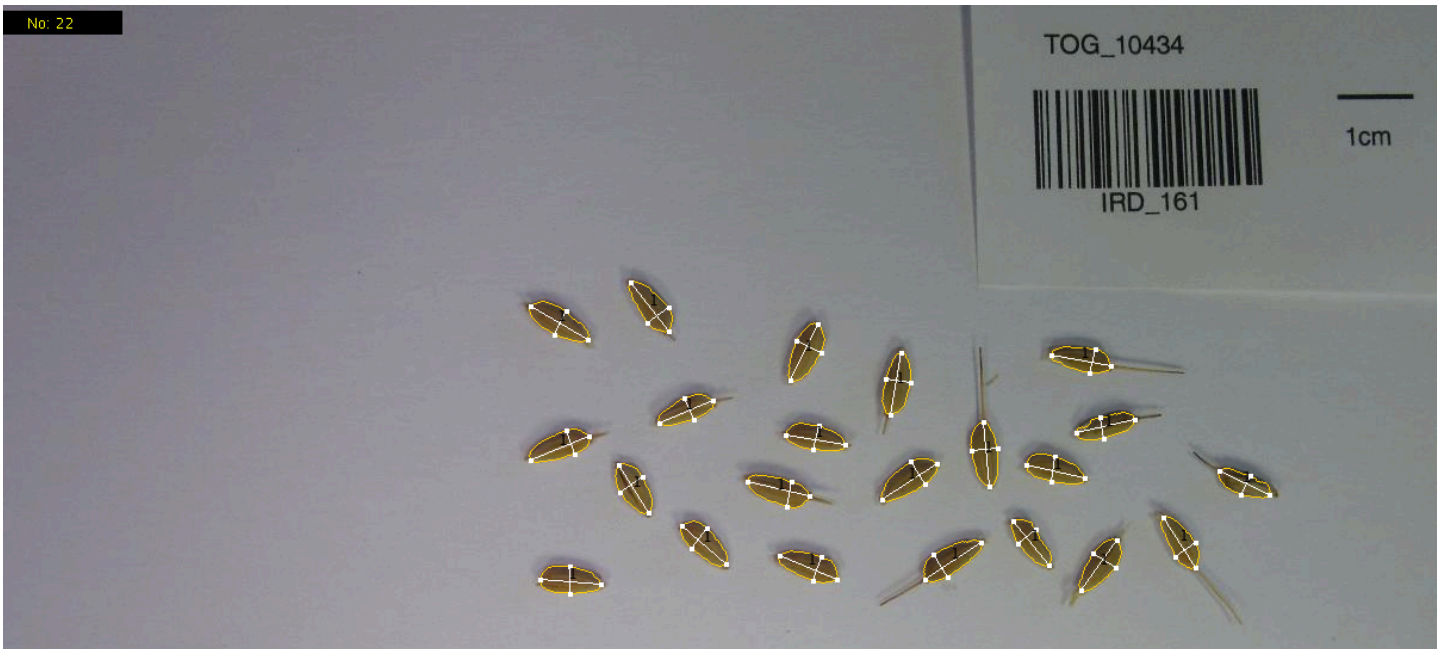

Uniformly spread grains

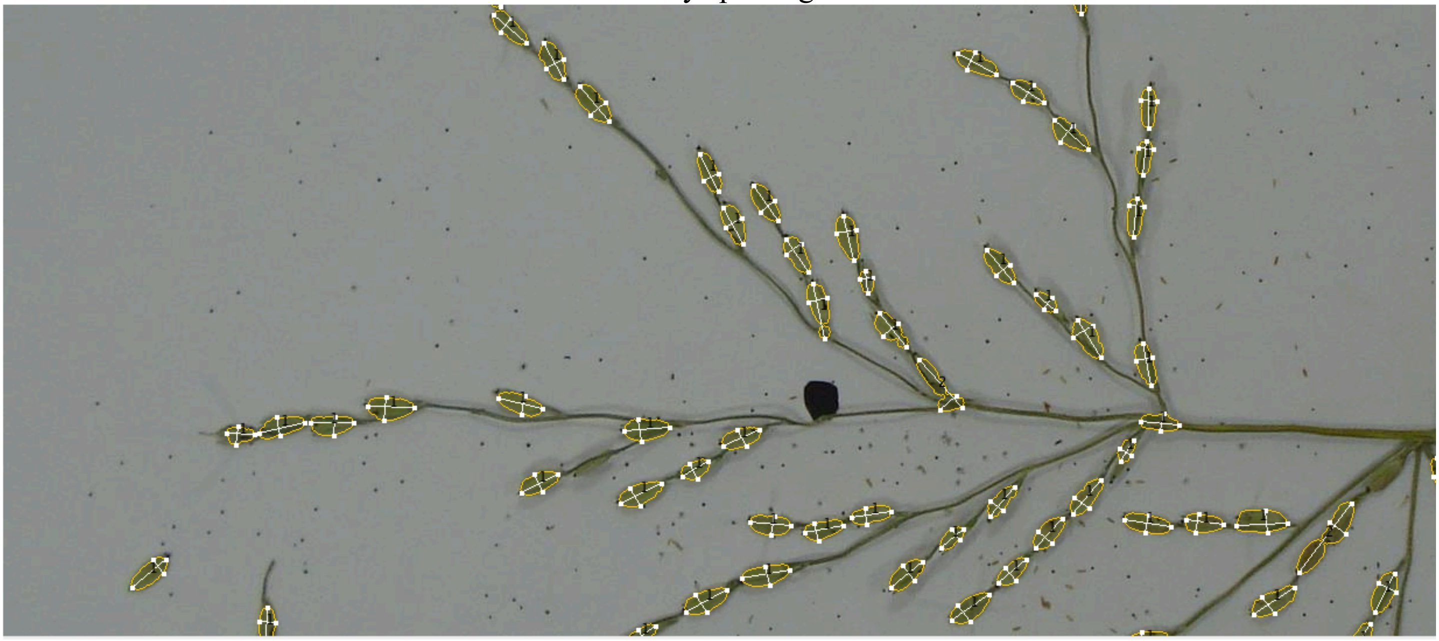

On-branch grains

3- Smartgrain Application Problems.

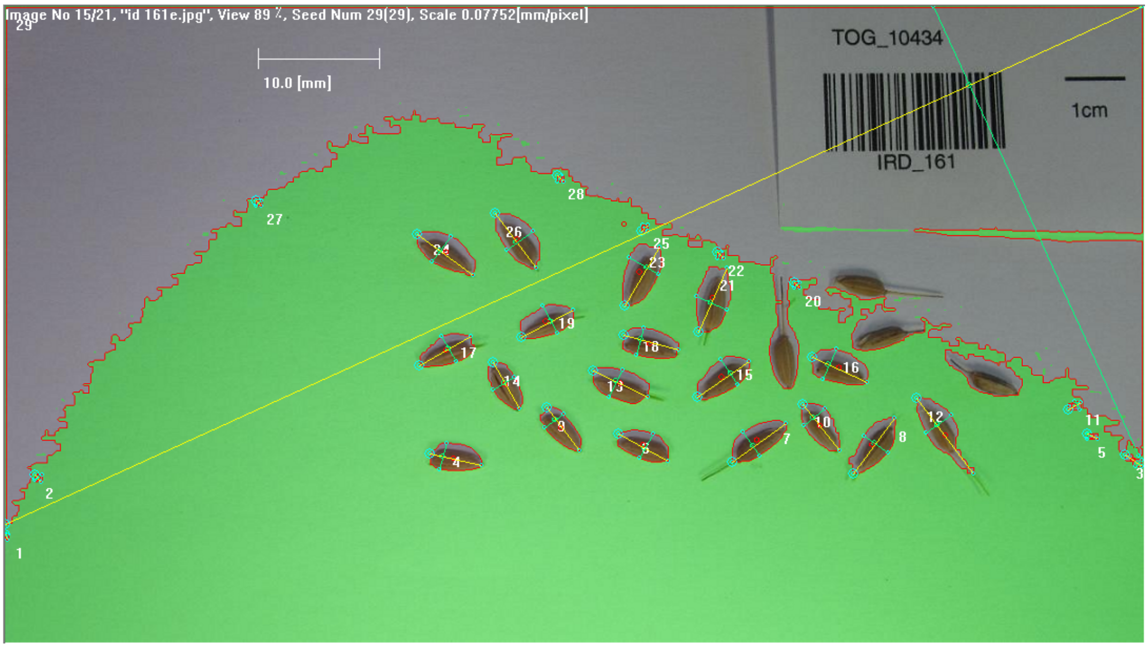

Smartgrain performs quit good in case of colored images when the user has to specify the background and object colors manually. In the case of grayscale mode, it is very sensitive to small illumination variations, as it seems to be using a global thresholding approach.

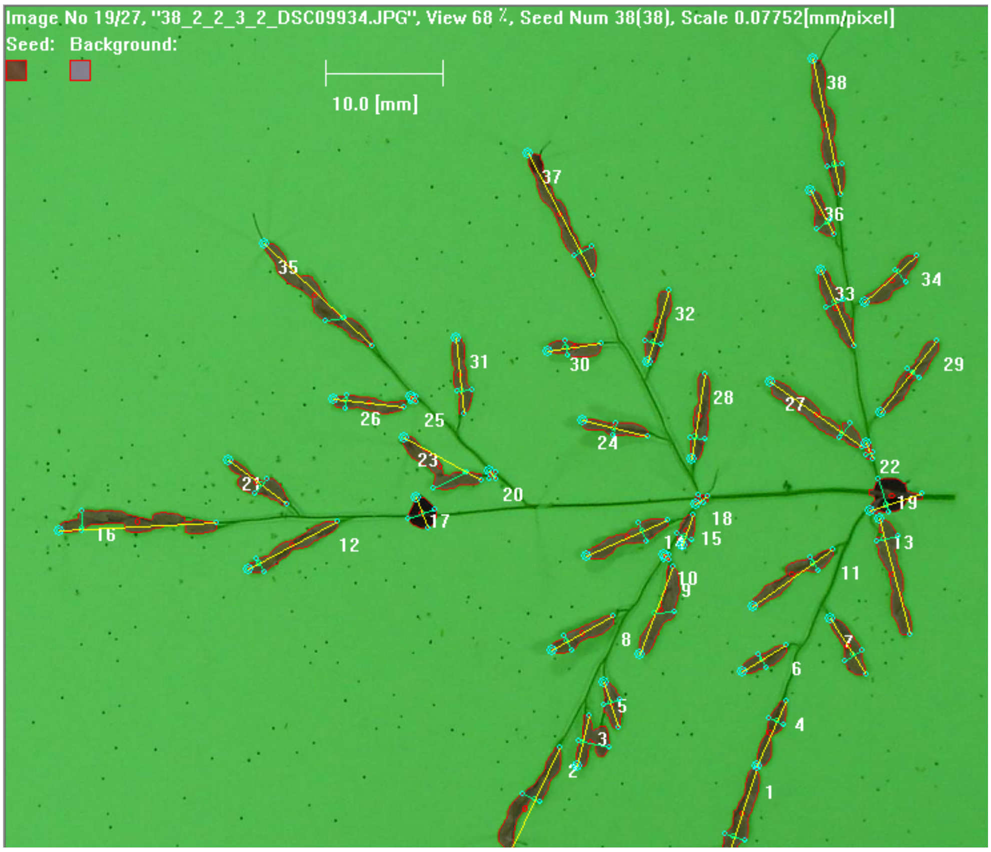

In case of on-branch grains, it is not designed to detect them and/or estimate their number by detecting a reference grain (grain size) and compare the other objects with respect to this reference.
